# Stacked but not Stuck: Unveiling the Role of π→π* Interactions with the Help of the Benzofuran–Formaldehyde Complex

**DOI:** 10.1002/anie.202113737

**Published:** 2021-11-23

**Authors:** Xiaolong Li, Lorenzo Spada, Silvia Alessandrini, Yang Zheng, Kevin Gregor Lengsfeld, Jens‐Uwe Grabow, Gang Feng, Cristina Puzzarini, Vincenzo Barone

**Affiliations:** ^1^ School of Chemistry and Chemical Engineering Chongqing University Daxuecheng South Rd. 55 Chongqing 401331 China; ^2^ Scuola Normale Superiore Piazza dei Cavalieri 7 56126 Pisa Italy; ^3^ Dipartimento di Chimica “Giacomo Ciamician” University of Bologna Via F. Selmi 2 40126 Bologna Italy; ^4^ Institut für Physikalische Chemie and Elektrochemie Gottfried Wilhelm Leibniz Universität Hannover Callinstrasse 3A 30167 Hannover Germany

**Keywords:** bond analysis, non-covalent interaction, quantum chemistry, rotational spectroscopy, structure determination

## Abstract

The 1:1 benzofuran–formaldehyde complex has been chosen as model system for analyzing π→π* interactions in supramolecular organizations involving heteroaromatic rings and carbonyl groups. A joint “rotational spectroscopy–quantum chemistry” strategy unveiled the dominant role of π→π* interactions in tuning the intermolecular interactions of such adduct. The exploration of the intermolecular potential energy surface led to the identification of 14 low‐energy minima, with 4 stacked isomers being more stable than those linked by hydrogen bond or lone‐pair→π interactions. All energy minima are separated by loose transition states, thus suggesting an effective relaxation to the global minimum under the experimental conditions. This expectation has been confirmed by the experimental detection of only one species, which was unambiguously assigned owing to the computation of accurate spectroscopic parameters and the characterization of 11 isotopologues. The large number of isotopic species opened the way to the determination of the first semi‐experimental equilibrium structure for a molecular complex of such a dimension.

## Graphical abstract

A π→π* interaction is discovered to be by far the main stabilizing contribution in the benzofuran–formaldehyde adduct, resulting in an interaction energy of −16.1 kJ mol^−1^. The characterization of eleven singly‐substituted isotopologues allowed for an accurate semi‐experimental equilibrium structure determination, which points out a carbon–carbon distance (3.2257±0.0006 Å) between formaldehyde and benzofuran shorter than the sum of the carbon van der Waals radii.

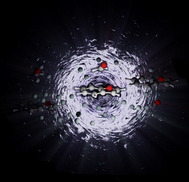

Aromatic compounds play a key role in tuning the structure of biomolecules and influencing several processes related to the origin of life and its evolution (e.g. photosynthesis,^[1]^ storage of information,^[2]^ DNA replication^[3]^). In this context, the non‐covalent interaction (NCI)‐mediated processes ruled by aromatic moieties are of paramount importance^[4]^ for several fields ranging from molecular recognition^[5]^ to catalysis,[^5c^, ^6^] crystal engineering,^[7]^ and drug delivery.^[8]^ Indeed, the versatility of the aromatic groups is reflected by the number, types, and specificity of NCIs they can form.^[9]^ Among aromatic compounds, heteroaromatics are particularly important because, as part of nucleobases, they are building blocks of life and play a central role in medicinal chemistry.^[10]^ In this realm, an intriguing class of molecules is represented by benzofurans (BZF), whose structural unit is the heteroaromatic‐base scaffold of a large series of compounds exhibiting a variety of pharmacological activities.^[11]^ In this molecule, the π‐system and the oxygen sigma lone pair are competing sites in establishing NCIs.^[12]^ As an example, the structure of the complex formed between the human activated blood coagulation factor X (FXa) with (*S*)‐2‐cyano‐1‐(2‐methylbenzofuran‐5‐yl)‐3‐(2‐oxo‐1‐(2‐oxo‐2‐(pyrrolidin‐1yl) ethyl)azepan‐3‐yl)guanidine (PDB:3HPT^[13]^ YET 2.D ligand), reported in the protein data bank (PDB),^[14]^ points out close intermolecular contacts (with three of them showing distances shorter than 3.6 Å) between the BZF and carbonyl moieties (see Figure 1). While intra‐ and intermolecular interactions in biomolecules are of great interest because they mediate and/or are responsible for a specific activity, their accurate characterization is not easy because of the large dimension and, often, even qualitative pictures are difficult to obtain. A possible way‐out from such a limitation is offered by the investigation, at the state of the art, of the interaction of interest in a smaller model system. Taking the example above, as highlighted in Figure 1, a suitable model system might be formed by BZF and formamide. However, the BZF–formaldehyde (BZF–FA) complex could be a more effective choice for a number of reasons: *i*) the competition between (a limited number of) different NCIs is possible, which would be instead inhibited in the presence of strong N−H⋅⋅⋅π^[16]^ or N−H⋅⋅⋅O NCIs as in the case of formamide; *ii*) the dimension of FA allows the exploration of several specific interaction sites; *iii*) the polarity of the molecule could enhance the cooperativity between different effects, which is instead negligible for benzene complexes.[^17^, ^18^]

**Figure 1:**
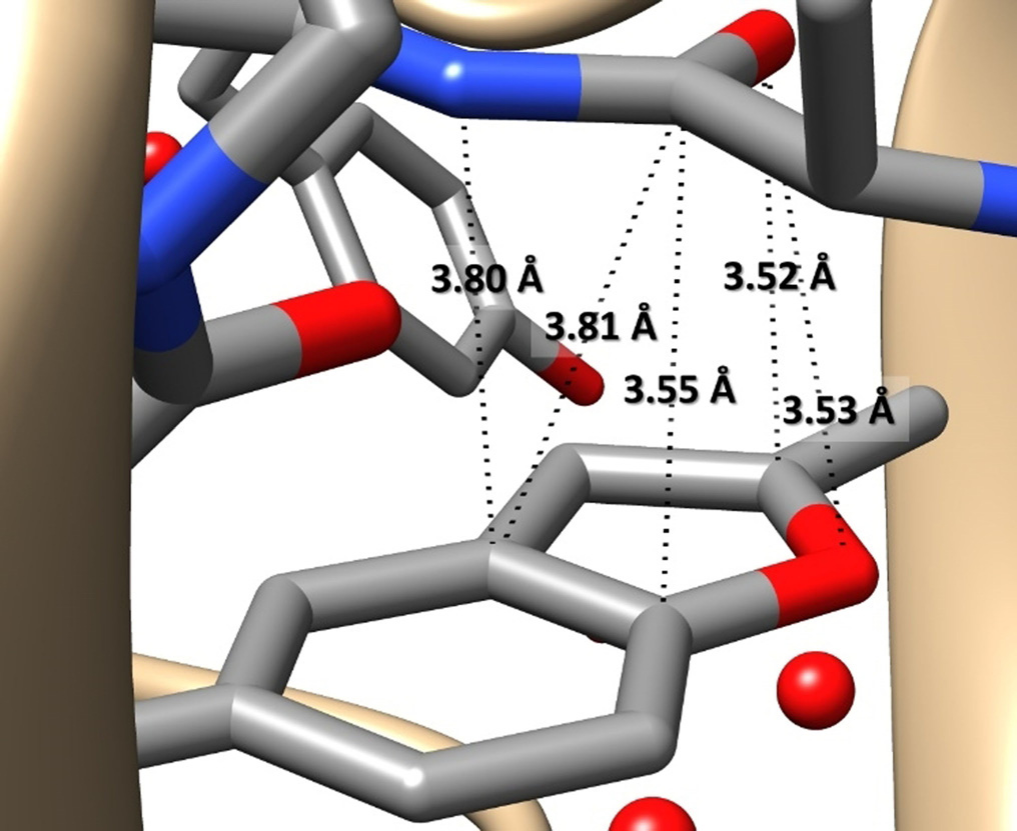
Region of the PDB:3HPT[^13^, ^14^] structure (see text for details) showing close intermolecular contacts between a (2‐methyl)‐BZF moiety of the YET 2.D ligand and a carbonyl group. The structure is visualized using the Chimera 1.14 software.^[15]^

The points addressed above suggest the BZF–FA adduct as a model for a deeper understanding of specific NCIs involving heteroaromatics. In turn, this requires the accurate determination of its structural parameters and interaction energies, which are necessary prerequisites for a number of tasks, ranging from disentangling the different contributions concurring to the stabilization of non‐covalent complexes to the analysis of the role played by cooperative (or anti‐cooperative) effects[^17^, ^18^, ^19^] when larger clusters are considered, not to mention benchmark studies of large systems. To achieve this goal, an integrated experiment‐theory approach has been exploited and, to avoid any perturbation from the environment, the investigation has been carried out in the gas phase. This is based, for the experimental core, on the supersonic expansion of the two interacting species (in view of forming binary complexes) into the high‐Q resonator (a solvent and matrix free environment) of a microwave spectrometer. This rotational coherence technique has proven to be well suited for the characterization of weakly bonded isomers.^[20]^ However, full exploitation of this kind of high‐precision studies relies on the support of high‐accuracy quantum‐chemical computations. Since several nearly‐isoenergetic isomers can be simultaneously present, the help of theory is required^[21]^ not only for the interpretation of the experimental observations, but also for a quantitative characterization of the energetics and structural parameters of the observed isomer(s).

Based on previous studies,[^22^, ^23^] the main stationary points on the potential energy surface (PES) of the BZF–FA adduct, that is, its minima and the transition states ruling their interconversion, have been located employing the double‐hybrid B2PLYP‐D3(BJ) functional in conjunction with the jun‐cc‐pVTZ basis set[^24^, ^25^, ^26^] (hereafter jB2), also incorporating the counterpoise (CP) correction^[27]^ (hereafter CP‐jB2). Improved electronic energies of all the identified stationary points have been computed using the composite scheme denoted as jun‐ChS, whose accuracy has been recently validated.^[22]^ The Gaussian 16 package^[28]^ has been used throughout and a complete account of the computational details is provided in the Supporting Information (SI).

Fourteen low‐energy minima have been found on the PES and their structures are collected in Table S1.1.1 of the SI. For all isomers, the parameters required for the spectroscopic investigation (rotational constants and electric dipole moment components) together with their relative energies are provided in Table S1.1.2 of the SI. The four lowest‐energy minima exhibit a nearly stacked arrangement of the BZF and FA moieties, with the latter lying above the bridging atoms in BZF (see Figure 2). These four minima differ in the orientation of the FA molecule with respect to BZF. Along this series, the dipole moment vector (DM) of the isolated BZF and the projection of the DM of FA on the BZF plane are oriented increasingly antiparallel from isomer ***IV*** to isomer ***I***. The latter has the largest dipole‐dipole energy contribution,^[29]^ as suggested by Figure 2 and then confirmed by a symmetry adapted perturbation theory (SAPT) analysis (vide infra). While the structural description of the other isomers identified on the PES is provided in the SI, Figure 2 (for the four stacked minima) and Table S1.1.2 (for all the other minima) show that the interconversion barriers are low (also including those ruling the relaxation path of the most stable hydrogen‐bonded structure, that is, the isomer ***VII***), thus allowing the collisional relaxation of all isomers to the species ***I*** during the supersonic jet‐expansion.^[30]^

**Figure 2:**
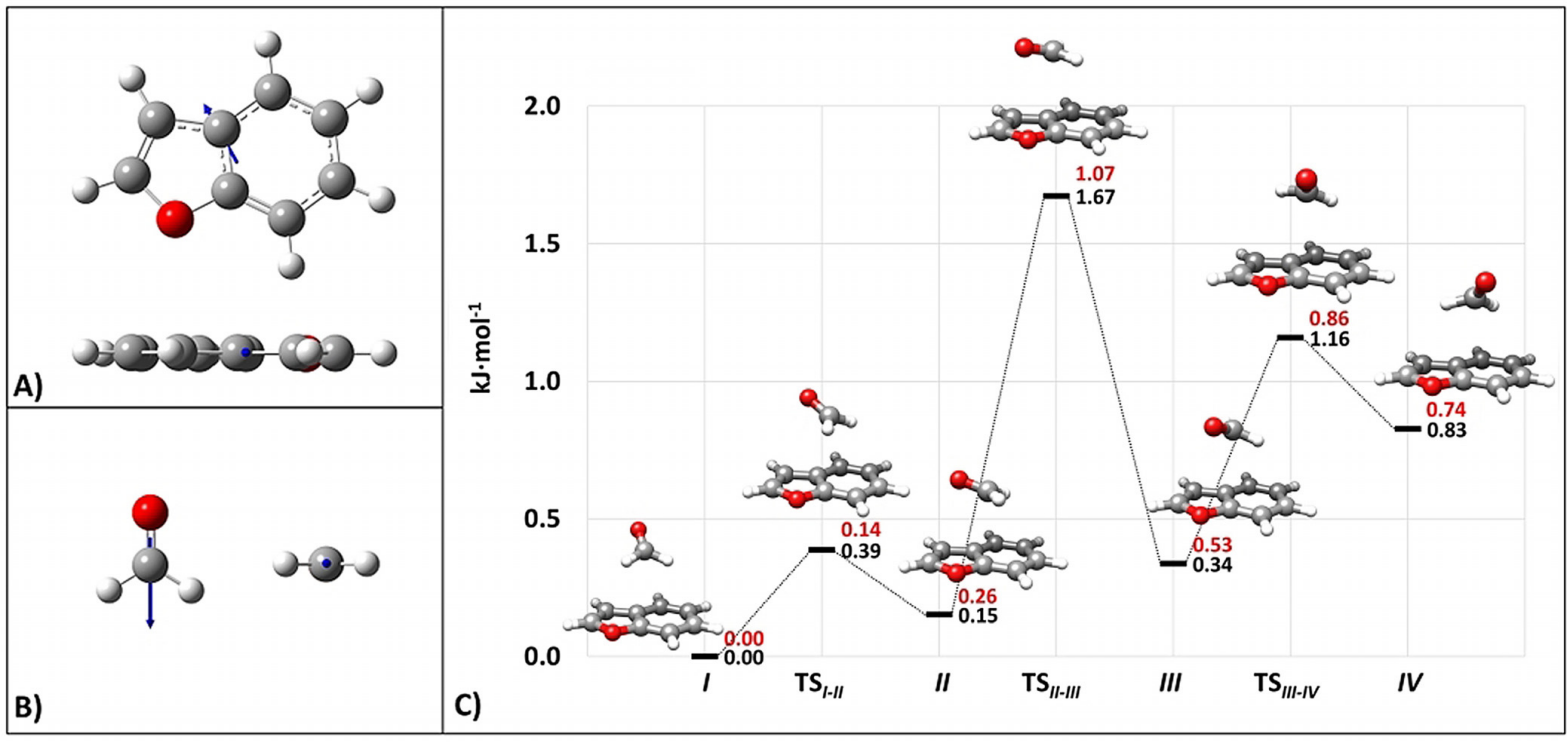
BZF (DM: 0.74 D) and FA (DM: 2.41 D) with their electric dipole moment vectors (at the jB2 level) are depicted in panels (A) and (B), respectively. The dipole moment vectors are not drawn to scale. Panel (C) shows the portion of the PES of the BZF–FA complex limited to the four lowest energy minima and the corresponding transition states: the relative jun‐ChS equilibrium energies (in black) and the harmonic ZPE‐corrected counterparts (in red) are displayed. ZPE corrections are at the CP‐jB2 level.

Experimentally, using a resonator‐based pulsed‐jet FTMW spectrometer^[31]^ (see the SI for a complete account on experimental details), it was possible to prove the presence of only one species (isomer ***I***) in the helium supersonic expansion of BZF and FA, thus supporting the relaxation toward the most stable isomer predicted by computations. In the recorded spectra, a 1:3 intensity ratio is observed (see Figure 3 A), which is typical of transitions belonging to the two states (labeled as *υ*=0 and *υ*=1) arising from the interchange of equivalent fermions (hydrogens of FA, having nuclear spin *I*=1/2). This fine structure suggests a “rolling” of the FA hydrogens with respect to the carbonyl axis. However, this tunneling splitting was not always resolvable (see Figure 3 B). From the analysis of the recorded spectra, accomplished by means of the Pickett's SPFIT program^[32]^ (employing Watson's *S*‐reduced Hamiltonian in *III* ^*l*^ representation),^[33]^ a set of rotational parameters has been obtained for each state and reported in Table 1. However, the spectroscopic assignment to the isomer ***I*** was not at all straightforward when relying on the usual practice of comparing the experimentally derived rotational constants with the calculated equilibrium counterparts. Indeed, the experimental parameters lie between those computed at the CP‐jB2 level for the isomers ***I*** and ***IV*** (see Table S1.1.2). Instead, once the vibrational corrections are incorporated in the computed constants, the assignment to the isomer ***I*** leaves little doubt, as evident from the comparison of Table 1. Vibrational corrections to rotational constants have been obtained from anharmonic CP‐corrected B3LYP‐D3(BJ)/SNSD[^25^, ^27^, ^33^, ^34^] computations (hereafter CP‐B3) in the framework of second‐order vibrational perturbation theory (VPT2)^[35]^ (see the SI for details). With inspection of Table 1 leaving only little space for doubt, the unbiased assignment to the isomer ***I*** was eventually achieved by recording, assigning and fitting the spectra of the complexes corresponding to all the monosubstituted ^13^C‐isotopologues of BZF and FA in natural abundance, as well as ^18^O‐FA, which was produced in situ by isotopic exchange (adding a small amount of H_2_^18^O to the gas mixture^[36]^). In the case of the ^18^O‐FA isotopologue, the rotational parameters of both *υ*=0 and *υ*=1 states were successfully fitted, while for the ^13^C species only the strongest *υ*=1 lines were observed. It is noted that the experimental rotational constants of all these isotopic species (which are available in the section S2 of the SI) have been found consistent with those of the isomer ***I***.

**Figure 3:**
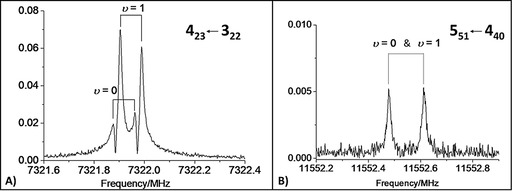
Examples of recorded rotational transitions belonging to the isomer ***I***: A) the *a*‐type *J*′_Ka′ Kc′_←*J*′′_Ka′′ Kc′′_=4_23_←3_22_ transition exhibiting the splitting due to tunneling (*υ*=0 and *υ*=1 states); B) the *b*‐type 5_51_←4_40_ transition with the tunneling states not being resolved.

**Table 1:** Spectroscopic parameters^[a]^ of the main isotopic species of the isomer ***I***.

Parameter	Experiment		Theory^[b]^	
	*υ*=0	*υ*=1	*Isomer **I***	*Isomer **IV***
*A*_0_ [MHz]	1180.9038(2)^[c]^	1180.9045(2)	1180.17	1190.64
*B*_0_ [MHz]	1096.1994(2)	1096.2033(2)	1103.11	1048.87
*C*_0_ [MHz]	788.2780(1)	788.2808(1)	789.01	763.23
*D*_J_^[d]^ [kHz]	3.902(2)	3.903(2)	2.5	2.5
*D*_JK_ [kHz]	−4.826(6)	−4.822(6)	−3.4	−3.2
*D*_K_ [kHz]	1.829(4)	1.828(4)	1.4	1.2
*d*_1_ [kHz]	−0.448(2)	−0.448(2)	−0.2	−0.4
*d*_2_ [kHz]	0.9641(8)	0.9639(8)	0.7	−0.1
*N* ^[e]^	204			
*σ* ^[f]^ [kHz]	2.7	

[a] Watson's *S*‐reduction, *III* ^l^ representation. [b] Vibrationally averaged rotational constants obtained by correcting the equilibrium CP‐jB2 rotational constants for vibrational corrections at the CP‐B3 level (see the SI for additional details). [c] Standard errors in units of the last digit. [d] Quartic centrifugal‐distortion constants (*D* and *d*). [e] Number of lines employed in the fit; [f] Root‐mean square error of the fit.

For the isomer ***I***, the availability of a large set of rotational constants (eleven isotopologues for the *υ*=1 state) allows the determination of a semi‐experimental (SE) equilibrium structure (*r*_e_^SE^)^[37]^ by exploiting the SE approach in combination with the template model (TM) approach,^[38]^ which are both detailed in the SI. Briefly, the structures of the BZF and FA frames within the complex have been accurately determined using the TM approach starting from the *r*_e_^SE^ values of the isolated fragments. The *r*_e_^SE^ of FA was taken from the SMART Lab database,^[39]^ while that of BZF has been purposely evaluated in this work based on the experimental data from ref. [40]. Then, the SE intermolecular parameters of the complex were obtained from a least‐squares fit of the SE equilibrium rotational constants. The latter were derived by correcting the experimental ground‐state rotational constants for the computed vibrational contributions (at the CP‐B3 level). Since the focus is on the intermolecular parameters describing the NCI and the TM approach ensures very accurate structures, the intramolecular parameters were kept fixed in the fitting procedure. Three structural intermolecular parameters have been determined (see Figure 4). These are the C(FA)⋅⋅⋅C_β_ distance (3.2257±0.0006 Å), the C(FA)⋅⋅⋅C_β_–C_4_ angle (90.18±0.04°), and the C(FA)⋅⋅⋅C_β_‐C_4_‐C_3_ dihedral angle (−98.31±0.03°). The good agreement between the CP‐jB2 intermolecular parameters and the SE counterparts, with discrepancies well within 1 % (see Figure 4), demonstrates the reliability of the CP‐jB2 geometries and, consequently, of the corresponding equilibrium rotational constants. The presence of an interaction between BZF and FA is clearly reflected by the C(FA)⋅⋅⋅C_β_ distance, which is shorter than the sum of carbon van der Waals radii (3.40 Å).^[41]^

**Figure 4:**
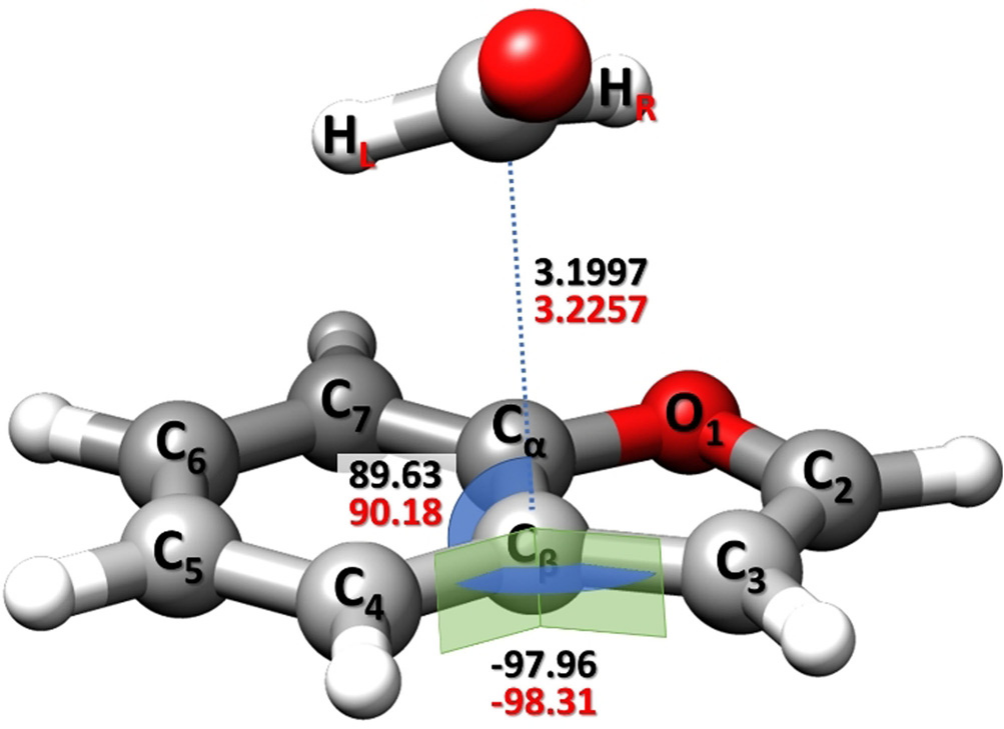
SE equilibrium intermolecular parameters (in red) of the isomer ***I*** compared to the CP‐jB2 counterparts (in black).

The intermolecular parameters follow the trends suggested by a natural bond orbital (NBO) analysis^[42]^ performed at the B3LYP‐D3(BJ)/maug‐cc‐pVTZ‐*d*H[^25^, ^35^, ^43^] level (hereafter mB3) using the CP‐jB2 geometry as well as the *r*_e_^SE^ structure. The NBO analysis (the two geometries provide nearly identical results) indicates that the isomer ***I*** earns most of its stability from a π–π* interaction (see Figure 5 A and Table 2). This is accompanied by different (weak) C−H⋅⋅⋅π hydrogen bond (HB) contributions (only that >0.5 kJ mol^−1^ is reported in Table 2; for the complete list, see the SI). The same CP‐jB2 structure was used for analyzing the reduced density gradient (Figure 5 B) and visualizing the so‐called NCI plot^[44]^ (Figure 5 C); both of them show a broad attractive interaction zone above the BZF plane. From a more quantitative point of view, a Natural Energy Decomposition Analysis (NEDA),^[45]^ carried out at the mB3 level, points out that the electrical (−22.6 kJ mol^−1^) and charge transfer (−18.3 kJ mol^−1^) contributions overcome the core repulsion (24.2 kJ mol^−1^), thus resulting in a total interaction energy of −16.7 kJ mol^−1^ (using the CP‐jB2 geometry, −16.6 kJ mol^−1^ employing the *r*_e_^SE^ structure; see the SI for further details). Since dispersion interactions are not explicitly accounted for in the NEDA, we also performed a SAPT2+(3)δMP2/aug‐cc‐pVTZ[^46^, ^47^] energy decomposition analysis on top of CP‐jB2 geometries (for the analysis description and explanation of the acronym, see the SI). Based on the full account for all isomers (Table S1.5.1) and that for the reduced model of the PDB:3HPT structure (Table S1.6.1), the SAPT analysis emphasizes the prominent role always played by dispersions in the complex stabilization. For isomer ***I***, this term amounts to −20.9 kJ mol^−1^, followed by a significant electrostatic contribution (−11.8 kJ mol^−1^) and a small induction term (−3.4 kJ mol^−1^), resulting in a total interaction energy of −16.3 kJ mol^−1^. Thus, NEDA and SAPT analyses show a remarkable agreement with the jun‐ChS interaction energy (−16.1 kJ mol^−1^, see the SI). The larger interaction energy of the BZF–FA complex with respect to the stacked benzene dimer (−11.6 kJ mol^−1^ at the jun‐ChS level^[22a]^) points out the role of dipolar interactions in enhancing π⋅⋅⋅π* interactions.

**Figure 5:**
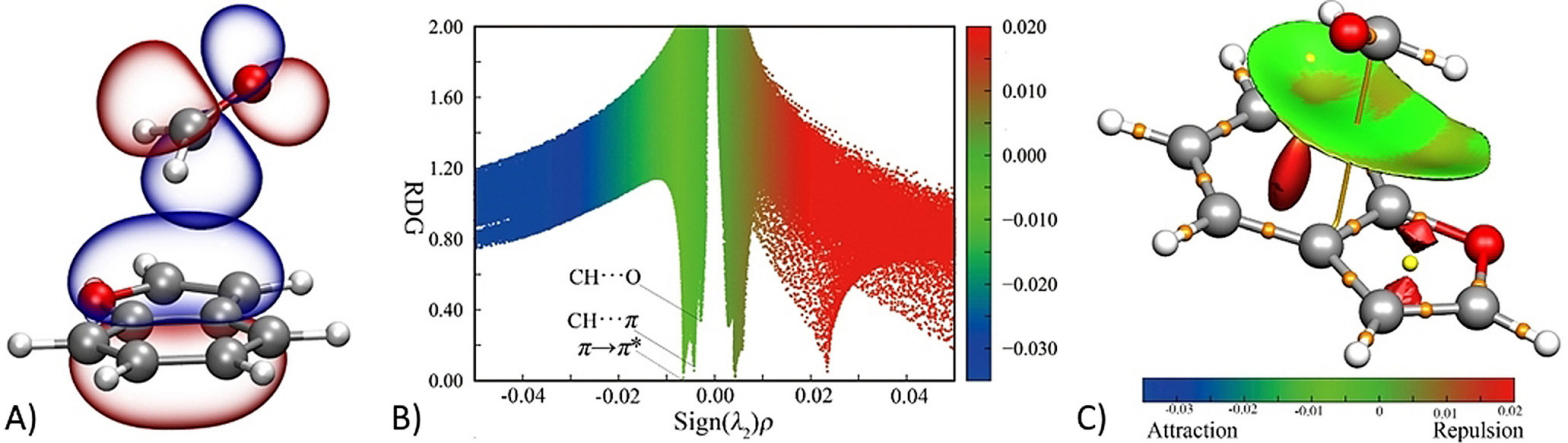
Isomer ***I***: A) NBO representation of the highest occupied *π* orbital of BZF and the antibonding (π*) orbital of FA; B) Reduced density gradient (RDG) representation; C) The NCI plot: blue and green surfaces show the presence of strong and weak attractive interactions, respectively, while the red one indicates repulsive interactions.

**Table 2:** NBO7 *E* ^(2)^ contributions >0.5 kJ mol^−1^ at the mB3 level (on top of the CP‐jB2 geometry and, within parentheses, *r*_e_^SE^). See Figure 4 for atom labeling.

Donor NBO	Acceptor NBO	*E* ^(2)^ [kJ mol^−1^]
BD(2) C_β_‐C_α_	BD*(1) C‐O (FA)	4.3 (4.0)
BD(2) C_7_‐C_6_	BD*(1) C‐H_L_ (FA)	0.6 (0.6)

In summary, a π–π* bonded complex between BZF and FA has been characterized by combining rotational spectroscopy in supersonic expansion with high‐level quantum‐chemical computations. The resulting interaction energy (−16.1 kJ mol^−1^) is larger than that of the prototypical aromatic benzene dimer (−11.6 kJ mol^−1^),^[22a]^ but much lower than classical HBs such as the N⋅⋅⋅H‐O NCI formed in the pyridine‐water complex (−29.8 kJ mol^−1^).^[22a]^

The rotational assignment of eleven singly‐substituted isotopic species holds a large amount of structural information (see, e.g., refs. [20f, 20k]) that allowed the determination of the SE equilibrium structure, the most accurate geometry for medium to large systems.^[37]^ This result is particularly significant because, in several previous studies on non‐covalent complexes (see, e.g., refs. [20a, 20f, 20g, 20k]), the microwave investigations of different isotopic species were employed for the derivation, at most, of approximated vibrationally averaged structures (when not mixing computed equilibrium with experimentally vibrationally averaged parameters). Differently, our approach leads to the determination of an accurate equilibrium structure by rigorously correcting experimental rotational constants for computed vibrational contributions (examples for intermolecular complexes are available in refs. [20i, 20l, 23]). The latter have been obtained from anharmonic computations that are expensive for a system as large as BZF–FA. The good agreement of the CP‐jB2 structure with *r*_e_^SE^ (see Figure 4) and of the CP‐jB2 energetics with the jun‐ChS counterparts, points out the reliability of the CP‐jB2 level in describing a π–π* interacting system in isolated conditions.

Finally, focusing on the crystal structure of Figure 1 and its comparison with BZF–FA isomers, some conclusions can be drawn:

1. The most stable structure in a matrix/solvent free environment, namely that observed in supersonic expansion, shows a different orientation of the FA moiety with respect to PDB:3HPT (YET 2.D), which is instead more similar to the isomer ***II***.
2. The comparison between the model structure of PDB:3HPT (YET 2.D) (see Figure 1 and Table S1.6.1) and the isomer ***II*** (CP‐jB2 geometry) points out similar O(FA)⋅⋅⋅O(BZF) and O(FA)⋅⋅⋅C2(BZF) distances (3.53 Å vs. 3.64 Å and 3.52 Å vs. 3.59 Å, respectively). Instead, the C(FA)⋅⋅⋅⋅C_α_ contact is much shorter in the isomer ***II*** than in PDB:3HPT (3.09 Å vs. 3.55 Å, respectively).
3. The above considerations, together with the NBO analysis of a selected portion of the PDB:3HPT crystal (at the same level as BZF–FA) chosen to mimic the frame where BZF (YET 2.D fragment) is inserted, clearly point out that, in the crystal, the stabilization contribution of such a π–π* interaction (about 1 kJ mol^−1^) is less strong than that of the model structure in the gas phase (about 4 kJ mol^−1^; see Table 2), and comparable to the C−H⋅⋅⋅π NCIs (see NBO results in Table S1.6.2 of the SI).
4. Our results allow for an unbiased structural and energetic characterization of π–π* interactions between a carbonyl group and an heteroaromatic moiety, thus permitting the disentanglement of the different contributions ruling the behavior of more complex systems.

## Conflict of interest

The authors declare no conflict of interest.

## Supporting information

As a service to our authors and readers, this journal provides supporting information supplied by the authors. Such materials are peer reviewed and may be re‐organized for online delivery, but are not copy‐edited or typeset. Technical support issues arising from supporting information (other than missing files) should be addressed to the authors.

Supporting Information
