# Supplementary material for: Stacked but not Stuck: Unveiling the Role of π→π* Interactions with the Help of the Benzofuran–Formaldehyde Complex
Source: Angew Chem Int Ed Engl. 2021 Nov 23;61(1):e202113737. doi: 10.1002/anie.202113737 (PMC9298890; doi:10.1002/anie.202113737)
Supplement: Supplementary file 1 — Supporting Information [file ANIE-61-0-s001.pdf]

Angewandte Chemie
Eine Zeitschrift der Gesellschaft Deutscher Chemiker

Supporting Information

Stacked but not Stuck: Unveiling the Role of π→π* Interactions with the Help of the Benzofuran–Formaldehyde Complex

Xiaolong Li[+], Lorenzo Spada[+], Silvia Alessandrini, Yang Zheng, Kevin Gregor Lengsfeld, Jens-Uwe Grabow, Gang Feng, Cristina Puzzarini, and Vincenzo Barone**

anie_202113737_sm_miscellaneous_information.pdf

Supporting Information

S1. Computational details:

S1.1. Conformational search and energetic characterization of the binary benzofuran-formaldehyde adducts.
S1.2. Rotational parameters.
S1.3. Natural Bond Orbital (NBO) analysis.
S1.4. Natural Energy Decomposition Analysis (NEDA).
S1.5. Symmetry Adapted Perturbation Theory (SAPT) Analysis.
S1.6. Analysis of non-covalent interactions in the PDB:3HPT YET 2.D ligand model.

S2. Experimental details:

S2.1. Description of the experimental setup and assignment procedure.
S2.2. Experimental rotational transitions and fitted parameters of isomer *I* of the Benzofuran-Formaldehyde complex for:
S2.2.1. The parent species.
S2.2.2. The BZF-(^{18}O)FA isotopologue.
S2.2.3. The ($^{13}C_2$)BZF-FA isotopologue.
S2.2.4. The ($^{13}C_3$)BZF-FA isotopologue.
S2.2.5. The ($^{13}C_\alpha$)BZF-FA isotopologue.
S2.2.6. The ($^{13}C_\beta$)BZF-FA isotopologue.
S2.2.7. The ($^{13}C_4$)BZF-FA isotopologue.
S2.2.8. The ($^{13}C_5$)BZF-FA isotopologue.
S2.2.9. The ($^{13}C_6$)BZF-FA isotopologue.
S2.2.10. The ($^{13}C_7$)BZF-FA isotopologue.
S2.2.11. The BZF-(^{13}C)FA isotopologue.

S3. Semi-experimental equilibrium structures:

S3.1. Theoretical background.
S3.2. Benzofuran.
S3.3. The isomer ***I*** of the Benzofuran-Formaldehyde complex.

S4. References

S1. Computational details

S1.1. Conformational search and energetic characterization of the binary benzofuran-formaldehyde adducts.

Starting from the selection of a dozen of initial structures followed by a systematic search employing the CREST code,[1] a large number of low-energy stationary points on the potential energy surface (PES) were located at the B3LYP-D3(BJ)/SNSD[2] level (hereafter B3), where D3(BJ) stands for the D3 scheme for the treatment of dispersion effects combined with the Becke-Johnson (BJ) damping function. Then, true energy minima have been identified by diagonalization of analytical Hessians (all eigenvalues being positive) and reoptimized at a higher level of theory, thereby using the double-hybrid B2PLYP functional combined with the D3(BJ) correction and in conjunction with the jun-cc-pVTZ basis set (hereafter referred to as jB2).[2f,3] Both B3 and jB2 calculations incorporated the counterpoise correction (CP)[4] in each optimization step, thus leading to the levels of theory denoted as CP-B3 and CP-jB2, respectively. This procedure led to the characterization, at the CP-jB2 level, of fourteen low-energy minima (within 4 kJ mol^{-1} above the global minimum), whose Cartesian coordinates are reported in Table S1.1.1.
To get a more accurate evaluation of the relative energies of all minima and of the most relevant transition states, and to accurately estimate the interaction energies, the so-called “jun-Cheap” composite scheme (denoted as jun-ChS in the following and in the manuscript)[5] has been employed, incorporating the CP correction[4] in each energy contribution, on top of the CP-jB2 geometries. The starting point of this composite scheme is the coupled-cluster singles and doubles method augmented by a perturbative treatment of triple excitations, CCSD(T).[6] Briefly, the CP-corrected CCSD(T)/jun-cc-pVTZ energy (evaluated within the frozen-core, fc, approximation) has been improved by adding corrections accounting for the extrapolation to the complete basis set (CBS) limit and the core-valence (CV) correlation. These two contributions are evaluated employing the Møller-Plesset theory to second order (MP2).[7] The overall expression is:

$$E_{junChS} = E(\mathrm{CCSD(T)/junVTZ}) + \Delta E^{MP2/\infty} + \Delta E_{CV}^{MP2/wCVTZ} \quad (2)$$

where the CP-corrected fc-MP2 energies computed in conjunction with the jun-cc-pVTZ and jun-cc-pVQZ basis sets are extrapolated to the CBS limit by means of the n^{-3} formula.[8] The CV contribution is evaluated as the energy difference between CP-corrected all-electron and fc-MP2 calculations, both in the same cc-pwCVTZ basis set.[9] The obtained results are reported in Table S1.1.2, where the lowest normal mode frequencies are also given for all stationary point structures.
All calculations were performed using the Gaussian16 software.[10]

S1.2. Rotational parameters.

The equilibrium rotational constants for the fourteen isomers of the 1:1 benzofuran-formaldehyde (BZF-FA) complex mentioned above have been straightforwardly derived from the equilibrium geometries. For the isomers ***I*** and ***IV*** (see main text), vibrational ground-state rotational constants (B_0^i) have also been obtained by correcting the CP-jB2 equilibrium rotational constants (B_e^i) with vibrational corrections (ΔB_{vib}^i) at the CP-B3 level:

$$B_0^i = B_e^i(\mathrm{CP-jB2}) + \Delta B_{vib}^i(\mathrm{CP-B3}) \tag{1}$$

where B_e^i denotes the equilibrium rotational constant with respect to the *i*-th inertial axis (*i* = *a*,*b*,*c*), so that $B_e^a = A_e$. Details on the evaluation of the $\Delta B_{vib}^i(\mathrm{CP-B3})$ term are given in section S3.

For all isomers, the dipole moment components and equilibrium rotational constants at the CP-jB2 level are reported in Table S1.1.2.

All calculations were performed using the Gaussian16 software.[10]

Table S1.1.1. The CP-jB2 computed structures of the fourteen isomers of BZF-FA together with their corresponding Cartesian coordinates (Å).

ISOMER	PICTURE	CARTESIAN COORDINATES (Å)
I	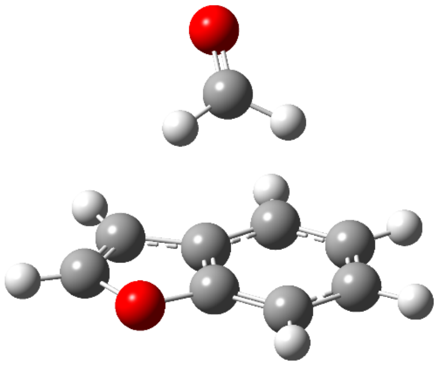	O -0.788793 -1.736892 -0.865408<br>C -0.168213 -2.412177 0.157191<br>C 0.116719 -1.605241 1.204601<br>C -0.355736 -0.295371 0.838709<br>C -0.902907 -0.440041 -0.445319<br>C -1.455634 0.604094 -1.168570<br>C -1.451662 1.850282 -0.552687<br>C -0.913472 2.028598 0.731983<br>C -0.363352 0.969044 1.437938<br>H -0.002804 -3.458636 -0.020410<br>H 0.605654 -1.898870 2.115973<br>H -1.866022 0.452470 -2.155396<br>H -1.871831 2.698495 -1.073093<br>H -0.926968 3.013655 1.175048<br>H 0.056445 1.118794 2.421943<br>C 2.245832 0.533490 -0.829507<br>O 3.256538 0.629645 -0.176564<br>H 1.963891 -0.410090 -1.325115<br>H 1.555170 1.379725 -0.966121
II	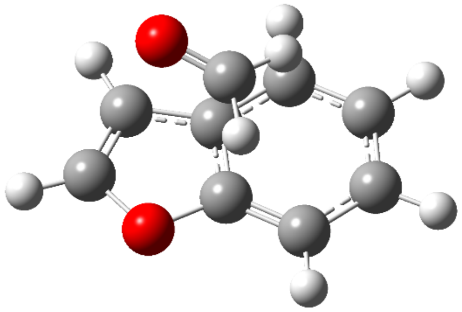	O 0.927556 1.127922 1.168438<br>C 1.622718 1.607887 0.085784<br>C 0.971148 1.390057 -1.078838<br>C -0.245671 0.706366 -0.732478<br>C -0.216658 0.575075 0.664900<br>C -1.213322 -0.045351 1.401168<br>C -2.281847 -0.564093 0.679440<br>C -2.338832 -0.454034 -0.719531<br>C -1.333160 0.178193 -1.436371<br>H 2.565760 2.067429 0.316010<br>H 1.318740 1.662591 -2.058844<br>H -1.155875 -0.120952 2.476598<br>H -3.085136 -1.059324 1.205132<br>H -3.186380 -0.870020 -1.244683<br>H -1.387457 0.256187 -2.512628<br>C 1.165766 -2.142112 0.178527<br>O 2.202346 -1.904833 -0.393124<br>H 1.119394 -2.244663 1.274908<br>H 0.214895 -2.268312 -0.361706
III	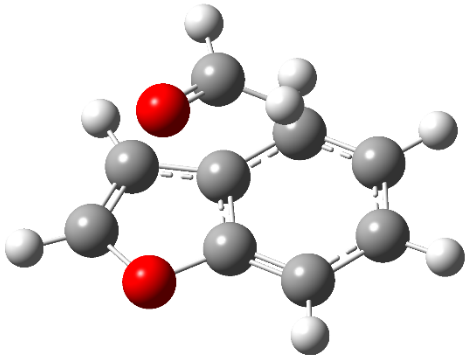	O 0.998179 -1.191258 -1.087216<br>C 1.654628 -1.616179 0.038450<br>C 0.958818 -1.351066 1.168355<br>C -0.248694 -0.691582 0.745189<br>C -0.164981 -0.622747 -0.654863<br>C -1.132906 -0.040042 -1.456181<br>C -2.233351 0.499345 -0.801398<br>C -2.347623 0.449255 0.597692<br>C -1.367863 -0.142715 1.381717<br>H 2.605951 -2.083430 -0.135771<br>H 1.266267 -1.587513 2.171188<br>H -1.027630 -0.003459 -2.529675<br>H -3.016302 0.967465 -1.379874<br>H -3.219511 0.879043 1.069207<br>H -1.467611 -0.177563 2.457245<br>C 1.092075 2.135992 0.467299<br>O 2.073131 2.080153 -0.233779<br>H 0.084297 2.291984 0.053100<br>H 1.156282 2.034371 1.563092

IV	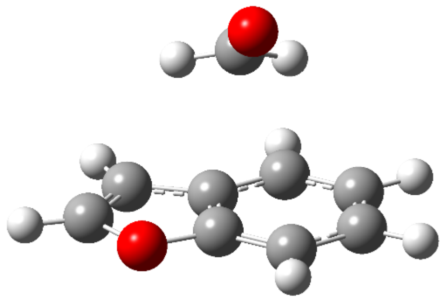	O -0.876261 -1.354998 -1.096978<br>C -1.584127 -1.912107 -0.062495<br>C -1.736088 -1.055192 0.973969<br>C -1.072695 0.162032 0.584954<br>C -0.563467 -0.084049 -0.699370<br>C 0.164692 0.840720 -1.429851<br>C 0.379215 2.072935 -0.824315<br>C -0.118234 2.352617 0.458430<br>C -0.842973 1.410193 1.173969<br>H -1.908855 -2.923532 -0.222795<br>H -2.253474 -1.260426 1.894005<br>H 0.547771 0.608663 -2.411768<br>H 0.943539 2.828922 -1.350089<br>H 0.070406 3.322688 0.894775<br>H -1.219612 1.636048 2.161246<br>C 2.221584 -0.792417 0.750960<br>O 3.265344 -1.074138 0.213883<br>H 1.979444 0.237682 1.054425<br>H 1.455730 -1.555646 0.966851
V	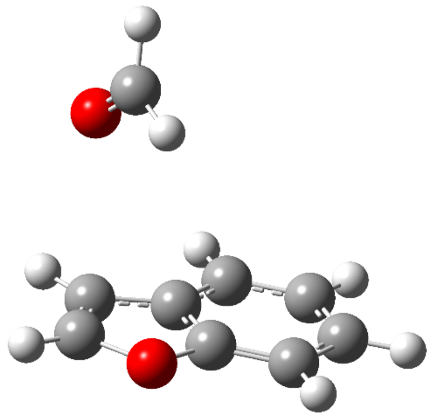	O -1.334115 1.847012 0.364883<br>C -0.175937 2.338946 -0.186183<br>C 0.596062 1.362242 -0.715687<br>C -0.115930 0.130273 -0.493655<br>C -1.294237 0.492647 0.175796<br>C -2.260242 -0.414820 0.578519<br>C -2.011761 -1.750179 0.284491<br>C -0.842310 -2.145545 -0.384752<br>C 0.112895 -1.220118 -0.779747<br>H -0.055696 3.404422 -0.118909<br>H 1.550569 1.486866 -1.194045<br>H -3.155256 -0.096642 1.091282<br>H -2.733962 -2.498857 0.575948<br>H -0.687573 -3.193753 -0.596279<br>H 1.013120 -1.528917 -1.290394<br>C 3.066258 -0.457443 1.042267<br>O 3.419801 -0.383479 -0.109796<br>H 3.775589 -0.708820 1.847328<br>H 2.021732 -0.278715 1.339426
VI	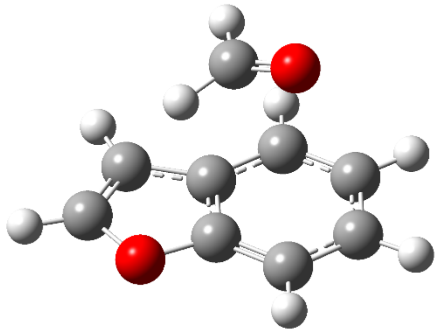	O 1.718117 -0.304808 1.129820<br>C 2.447457 -0.666469 0.025736<br>C 1.873934 -0.247553 -1.126468<br>C 0.669994 0.444349 -0.744334<br>C 0.628590 0.376957 0.656629<br>C -0.389449 0.917260 1.424120<br>C -1.411469 1.551964 0.730938<br>C -1.400649 1.639317 -0.670149<br>C -0.369980 1.093868 -1.419865<br>H 3.348981 -1.213435 0.231489<br>H 2.258318 -0.405904 -2.118286<br>H -0.390221 0.834920 2.500128<br>H -2.236166 1.980315 1.280921<br>H -2.217498 2.137768 -1.171094<br>H -0.372337 1.165296 -2.498125<br>C -1.213364 -2.101898 -0.364911<br>O -2.281392 -1.961639 0.177720<br>H -1.098649 -2.013798 -1.457043<br>H -0.295812 -2.328311 0.201420

VII	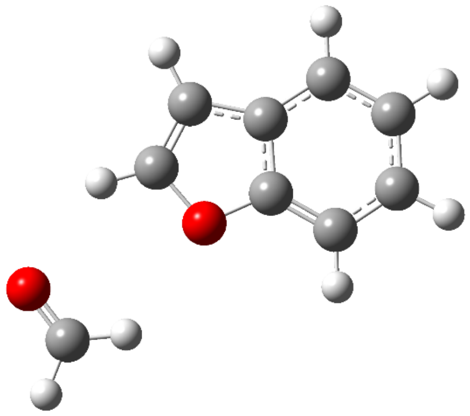	O 0.774716 -0.284833 0.000000<br>C 1.066884 1.061259 0.000000<br>C -0.052760 1.819658 0.000000<br>C -1.163221 0.902795 0.000000<br>C -0.590918 -0.377595 0.000000<br>C -1.327849 -1.549928 0.000000<br>C -2.710793 -1.410414 0.000000<br>C -3.315327 -0.143902 0.000000<br>C -2.557035 1.017971 0.000000<br>H 2.117025 1.292151 0.000000<br>H -0.085703 2.894445 0.000000<br>H -0.850233 -2.518111 0.000000<br>H -3.331907 -2.294230 0.000000<br>H -4.393588 -0.076387 0.000000<br>H -3.033293 1.987936 0.000000<br>C 4.072306 -1.017035 0.000000<br>O 4.262306 0.176386 0.000000<br>H 3.056793 -1.439098 0.000000<br>H 4.911327 -1.731069 0.000000
VIII	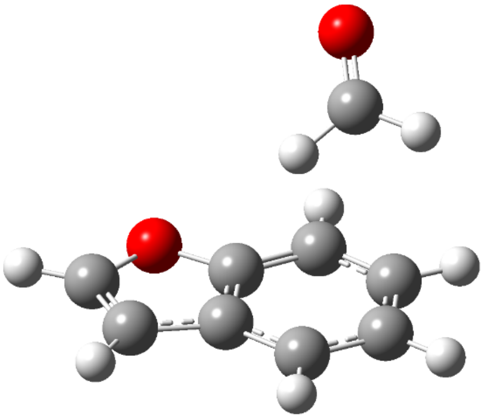	O -1.538086 -1.175056 0.917194<br>C -2.367571 -1.297081 -0.168120<br>C -2.135412 -0.344220 -1.100349<br>C -1.065892 0.467491 -0.581074<br>C -0.741030 -0.094307 0.663373<br>C 0.255933 0.393763 1.491678<br>C 0.948292 1.508467 1.033242<br>C 0.646969 2.096475 -0.205797<br>C -0.352571 1.587573 -1.022724<br>H -3.064848 -2.112945 -0.121365<br>H -2.656128 -0.232109 -2.034365<br>H 0.481649 -0.072955 2.438229<br>H 1.734427 1.929661 1.642727<br>H 1.206298 2.963771 -0.525766<br>H -0.575954 2.048106 -1.974396<br>C 2.594933 -0.763551 -0.770371<br>O 3.084456 -1.740158 -0.257263<br>H 3.175715 0.158662 -0.934613<br>H 1.546524 -0.739743 -1.103831
IX	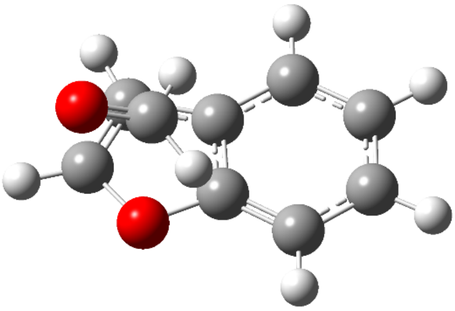	O 0.755940 0.535478 1.270058<br>C 1.105398 1.656679 0.557080<br>C 0.236451 1.913345 -0.447185<br>C -0.761087 0.876326 -0.388852<br>C -0.388916 0.060135 0.689865<br>C -1.090747 -1.067898 1.081852<br>C -2.223574 -1.377488 0.338543<br>C -2.623916 -0.581989 -0.746641<br>C -1.906197 0.544640 -1.120876<br>H 2.002203 2.146830 0.887392<br>H 0.298825 2.730718 -1.142971<br>H -0.773010 -1.667318 1.921614<br>H -2.808300 -2.246507 0.602437<br>H -3.511012 -0.855727 -1.299045<br>H -2.224412 1.149533 -1.957710<br>C 2.314033 -1.306495 -0.597337<br>O 3.470101 -0.967519 -0.661206<br>H 1.536088 -0.850244 -1.229713<br>H 1.974536 -2.090721 0.098670

X	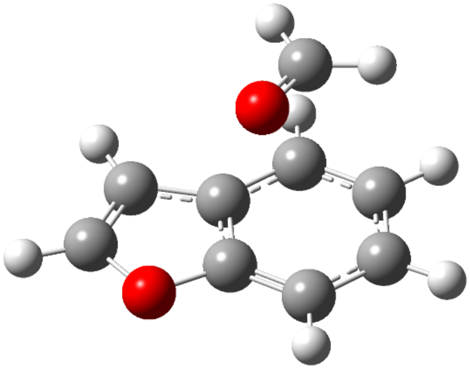	O -1.795051 0.237404 1.042433<br>C -2.486825 0.537107 -0.102112<br>C -1.830566 0.139712 -1.216864<br>C -0.607752 -0.467222 -0.761643<br>C -0.642977 -0.374573 0.637786<br>C 0.365169 -0.837637 1.466087<br>C 1.459301 -1.421118 0.840588<br>C 1.529690 -1.525656 -0.557969<br>C 0.506080 -1.055097 -1.370246<br>H -3.427656 1.033262 0.048348<br>H -2.170535 0.263344 -2.229456<br>H 0.302657 -0.737971 2.538758<br>H 2.274379 -1.797355 1.441374<br>H 2.396371 -1.988605 -1.007870<br>H 0.568673 -1.145974 -2.445475<br>C 1.959211 1.783475 -0.529396<br>O 1.324637 2.258933 0.379511<br>H 2.933454 1.296209 -0.367265<br>H 1.595933 1.810384 -1.568960
XI	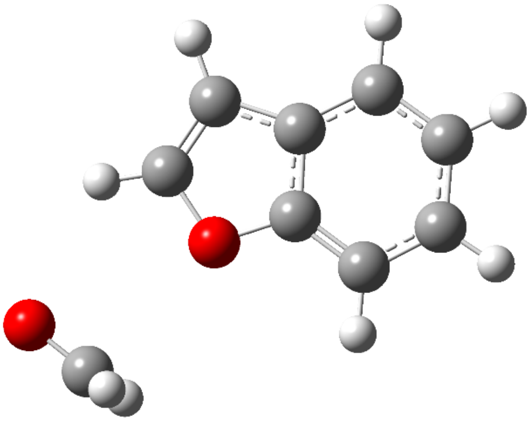	O -0.875867 -0.112638 0.000000<br>C -1.062532 1.250764 0.000000<br>C 0.111608 1.921125 0.000000<br>C 1.148232 0.921335 0.000000<br>C 0.479103 -0.310978 0.000000<br>C 1.122605 -1.536929 0.000000<br>C 2.512151 -1.504445 0.000000<br>C 3.212700 -0.288400 0.000000<br>C 2.546742 0.928673 0.000000<br>H -2.089210 1.567800 0.000000<br>H 0.226831 2.990186 0.000000<br>H 0.571998 -2.465548 0.000000<br>H 3.063149 -2.433545 0.000000<br>H 4.292907 -0.304547 0.000000<br>H 3.096487 1.858899 0.000000<br>C -3.604148 -1.076892 0.000000<br>O -4.130518 0.009479 0.000000<br>H -3.351122 -1.599367 0.935252<br>H -3.351122 -1.599367 -0.935252
XII	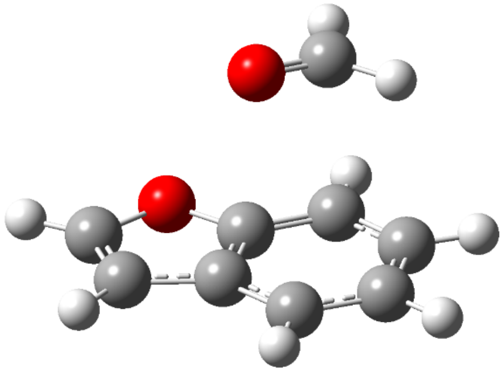	O 1.782223 -0.078733 -1.115983<br>C 2.476508 -0.580621 -0.042741<br>C 1.835609 -0.364782 1.128166<br>C 0.622878 0.334031 0.795167<br>C 0.647133 0.480730 -0.599533<br>C -0.358311 1.097145 -1.325564<br>C -1.435861 1.587456 -0.595020<br>C -1.488390 1.458899 0.802419<br>C -0.470233 0.835413 1.508317<br>H 3.404492 -1.064322 -0.285065<br>H 2.174118 -0.670911 2.101513<br>H -0.301907 1.192589 -2.399507<br>H -2.244788 2.082510 -1.112770<br>H -2.341892 1.853581 1.334515<br>H -0.525140 0.733572 2.582367<br>C -2.023393 -1.732400 -0.547542<br>O -1.351339 -2.337593 0.250550<br>H -2.936831 -1.194132 -0.250238<br>H -1.755878 -1.684171 -1.615086

XIII	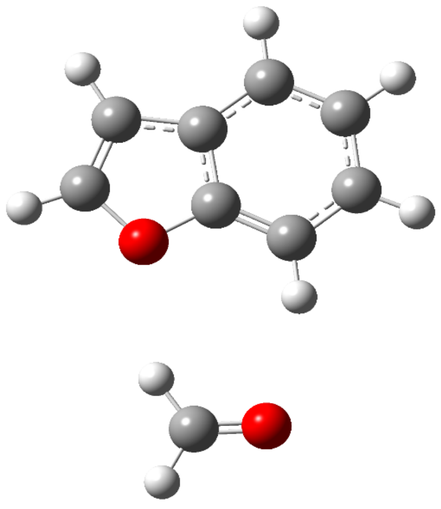	O 0.441276 1.328045 0.000000<br>C -0.479693 2.347349 0.000000<br>C -1.750956 1.886974 0.000000<br>C -1.656453 0.449842 0.000000<br>C -0.284249 0.162516 0.000000<br>C 0.240212 -1.119365 0.000000<br>C -0.686322 -2.155579 0.000000<br>C -2.067742 -1.905496 0.000000<br>C -2.567405 -0.611626 0.000000<br>H -0.066345 3.338981 0.000000<br>H -2.641393 2.489898 0.000000<br>H 1.305730 -1.298580 0.000000<br>H -0.333372 -3.176547 0.000000<br>H -2.753115 -2.740722 0.000000<br>H -3.632490 -0.429427 0.000000<br>C 3.796241 0.333695 0.000000<br>O 3.678145 -0.868878 0.000000<br>H 2.921574 1.000576 0.000000<br>H 4.789233 0.811777 0.000000
XIV	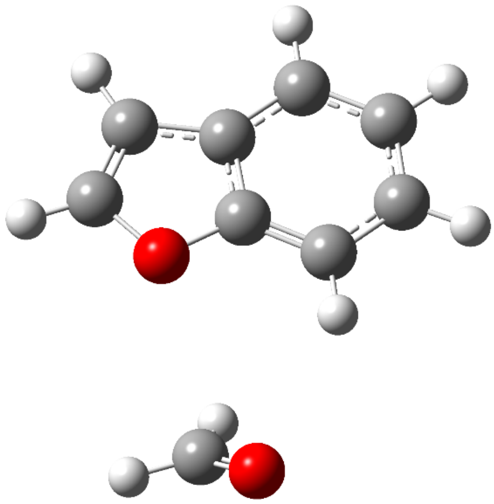	O 0.677946 1.143419 -0.386557<br>C -0.038865 2.293173 -0.159642<br>C -1.332857 2.037979 0.138293<br>C -1.476758 0.605336 0.103289<br>C -0.207834 0.107780 -0.226363<br>C 0.083682 -1.239956 -0.362298<br>C -0.973198 -2.117487 -0.150150<br>C -2.256303 -1.654000 0.180303<br>C -2.523407 -0.299113 0.310068<br>H 0.518978 3.206509 -0.253749<br>H -2.088931 2.772021 0.352479<br>H 1.075410 -1.583182 -0.615430<br>H -0.801932 -3.179959 -0.243324<br>H -3.050017 -2.370350 0.335449<br>H -3.514810 0.047595 0.563957<br>C 3.252809 -0.023132 0.654894<br>O 3.530012 -0.907313 -0.117999<br>H 2.453889 -0.134019 1.403710<br>H 3.786936 0.940297 0.649353

Table S1.1.2 CP-jB2 equilibrium rotational constants (A_e, B_e, C_e; in MHz), absolute values of the equilibrium dipole moment components ($|\mu_{e,a}|$, $|\mu_{e,b}|$, $|\mu_{e,c}|$; in debye), equilibrium relative energies (ΔE; in kJ·mol^{-1}) at the CP-jB2 and jun-ChS levels, harmonic ZPE-corrected energies (ΔE_0; in kJ·mol^{-1}) obtained by adding the CP-jB2 harmonic ZPE contributions. The interaction energies (IE; in kJ·mol^{-1}) and lowest normal mode frequencies (LNMF; in cm^{-1}) are reported for all energy minima and transition states.

MINIMA[a]												
Isomer	A_e	B_e	C_e	$\|\mu_{e,a}\|$	$\|\mu_{e,b}\|$	$\|\mu_{e,c}\|$	ΔE		ΔE_0		IE[a]	LNMF
							CP-jB2	jun-ChS	CP-jB2[a]	jun-ChS[a]	jun-ChS	
I	1189.40	1123.68	798.64	2.0	0.4	0.3	0.00	0.00	0.00	0.00	-16.15	+23
II	1419.94	1044.06	878.27	1.8	0.2	0.2	0.24	0.15	0.35	0.26	-16.01	+23
III	1373.64	1065.63	879.58	1.8	0.5	1.8	0.24	0.34	0.43	0.53	-15.75	+31
IV	1211.55	1068.19	774.45	2.8	0.7	1.5	0.72	0.83	0.63	0.74	-15.32	+22
V	1463.31	900.34	625.78	0.1	0.1	1.8	1.86	1.59	1.05	0.78	-14.48	+11
VI	1381.13	1045.21	864.11	2.2	0.1	1.6	1.93	2.08	1.79	1.95	-14.07	+28
VII	2883.80	542.31	456.47	0.2	1.5	0.0	2.05	1.84	1.68	1.47	-14.47	+14
VIII	1429.24	866.93	720.62	1.8	2.1	1.4	2.15	2.59	1.69	2.12	-13.51	+4
IX	1691.92	805.34	698.20	2.5	0.2	0.3	2.40	2.19	1.83	1.62	-13.89	+22
X	1425.68	1044.97	894.59	0.7	0.9	2.1	2.64	3.19	2.27	2.82	-12.91	+24
XI	2667.46	606.90	496.12	1.3	1.3	0.0	2.87	2.45	2.36	1.94	-13.57	+16
XII	1394.38	1047.50	878.67	0.7	1.0	0.6	2.92	3.23	2.11	2.42	-12.86	+9
XIII	1625.79	760.93	518.33	0.3	2.6	0.0	3.23	3.15	3.01	2.93	-13.08	+15
XIV	1624.89	848.89	577.87	1.2	2.1	1.6	3.67	3.33	3.44	3.10	-12.67	+17
**TRANSITION STATES**												
I-II	1282.25	1081.27	845.92				0.43	0.39	0.18	0.14		*i* 26
II-III	1487.51	988.75	853.95				1.45	1.67	0.85	1.07		*i* 55
III-IV	1184.64	1129.56	812.73				0.95	1.16	0.65	0.86		*i* 30
I-V	1427.83	948.95	650.72				2.23	2.33	1.41	1.51		*i* 24
IV-VI	1294.90	1061.17	830.66				2.07	2.24	1.52	1.69		*i* 33
VII-XI	2729.88	583.18	482.50				3.20	2.88	2.25	1.93		*i* 52
II-IX	1644.85	837.33	728.40				2.43	2.29	1.51	1.37		*i* 26
VI-X	1410.67	1012.88	875.82				2.97	3.38	2.34	2.75		*i* 26
IX-XI	2251.73	663.03	557.36				3.93	3.76	3.30	3.13		*i* 20

[a] For definition see equation (1) of ref. [5]

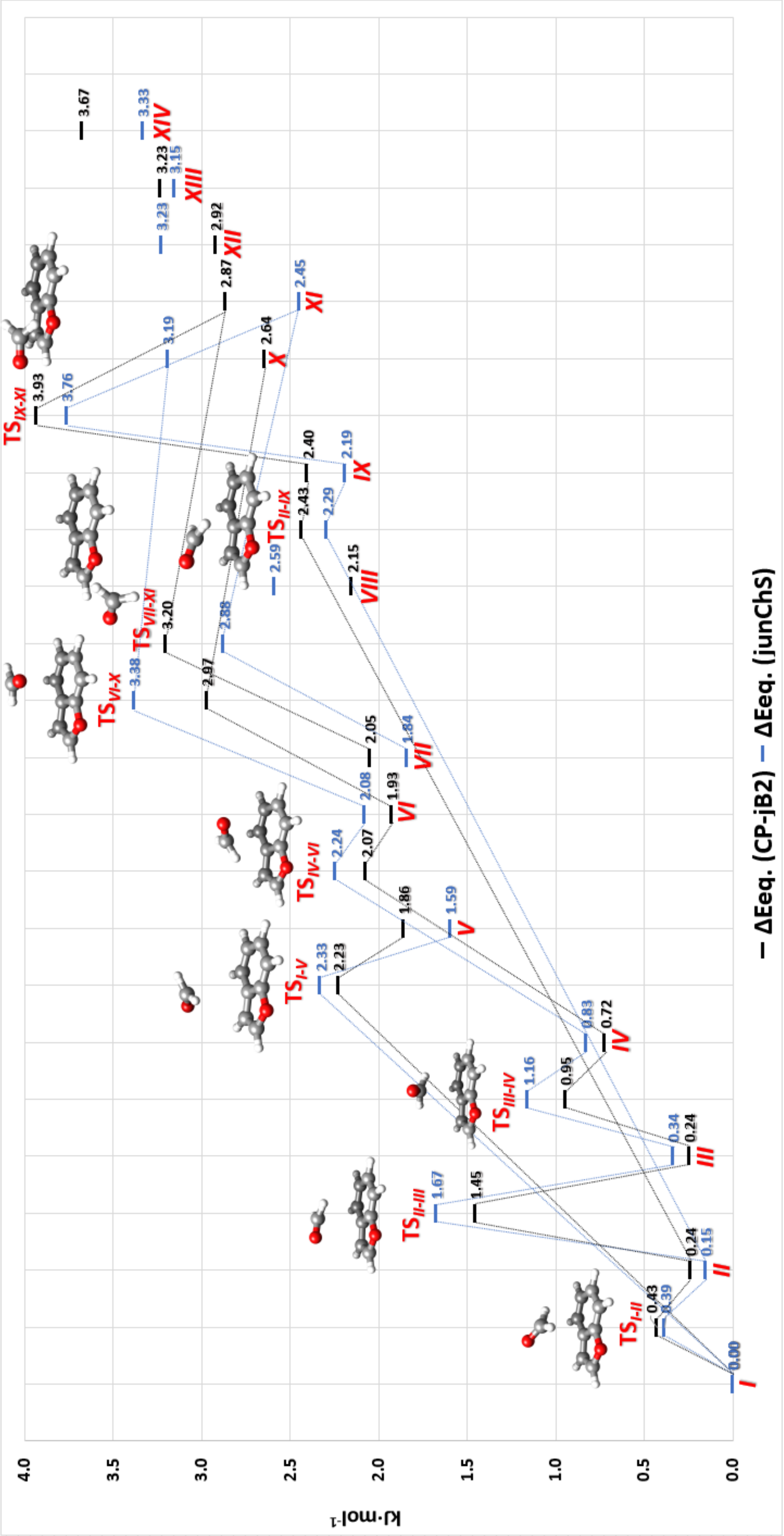

Figure SF1.1.1. Graphical representation of the relative jun-ChS and CP-jB2 equilibrium energies (ΔE_{eq}; in kJ·mol^{-1}) reported in Table S1.1.2. The structures of the transition states are also shown.

S1.3. Natural Bond Orbital (NBO) analysis.

For the fourteen low-lying minima, the Natural Bond Orbital (NBO) analysis has been performed, using the NBO7 program[11] interfaced to Gaussian16,[10] at the B3LYP-D3(BJ)/maug-cc-pVTZ-*d*H level of theory,[2,12,13] on top of the corresponding CP-jB2 geometries. For the isomer ***I***, for comparison purposes, the analysis has also been carried out using the semi-experimental equilibrium structure (r_e^{SE}; see section S3) as reference geometry. The results obtained are reported in Tables S1.3.1 to S1.3.14.

Table S1.3.1. NBO results for the Isomer ***I*** of the BZF-FA complex.

NBO Donor	NBO Acceptor	E(2) [kJ·mol^{-1}]	
		CP-jB2	r_e^{SE}
BD (2) C2- C3	BD*(1) C16- H18	0.2	0.2
BD (1) C4- C5	RY (3) C16	0.4	0.4
BD (2) C4- C5	BD*(1) C16- O17	4.3	4.0
BD (2) C4- C5	RY (4) C16	0.3	0.3
BD (1) C4- C9	RY (3) C16	0.3	0.2
BD (2) C6- C7	BD*(1) C16- H19	0.6	0.6
BD (2) C8- C9	BD*(1) C16- O17	0.3	0.2
BD (2) C8- C9	BD*(1) C16- H19	0.3	0.3
BD (1) C16- O17	BD*(2) C4- C5	0.3	0.3

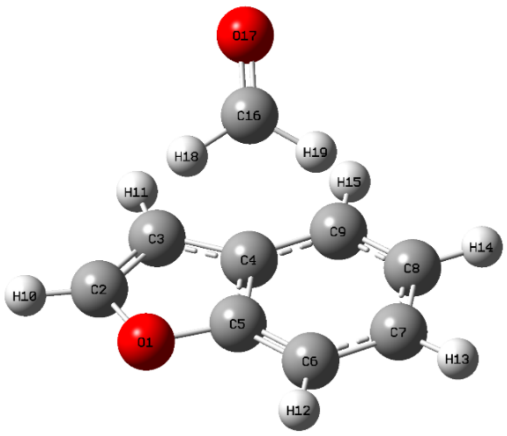

Table S1.3.2. NBO results for the Isomer ***II*** of the BZF-FA complex.

NBO Donor	NBO Acceptor	E(2) [kJ·mol^{-1}]
BD (2) C2- C3	BD*(1) C16- O17	0.4
BD (1) C4- C5	RY (3) C16	0.2
BD (2) C4- C5	BD*(1) C16- O17	4.1
BD (2) C4- C5	BD*(2) C16- O17	0.4
BD (2) C6- C7	BD*(1) C16- O17	0.5
BD (2) C6- C7	BD*(2) C16- O 17	0.2
BD (2) C8- C9	BD*(1) C16- H19	0.7
BD (2) C16- O17	RY (3) C4	0.5
BD (1) C16- H18	BD*(2) C4- C5	0.6
BD (1) C16- H18	BD*(2) C6- C7	0.3

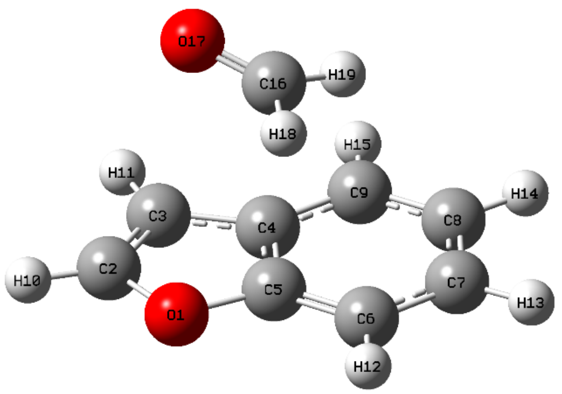

Table S1.3.3. NBO results for the Isomer ***III*** of the BZF-FA complex.

NBO Donor	NBO Acceptor	E(2) [kJ·mol^{-1}]
LP (2) O1	RY (3) O17	0.3
BD (1) C3- C4	RY (3) C16	0.2
BD (1) C4- C5	RY (3) C16	0.5
BD (2) C4- C5	BD*(1) C16- O17	4.9
BD (2) C4- C5	BD*(2) C16- O17	0.2
BD (1) C4- C9	RY (3) C16	0.3
BD (2) C6- C7	BD*(1) C16- H18	0.8
BD (2) C8- C9	BD*(1) C16- O17	0.2
BD (2) C16- O17	RY (4) C5	0.2
BD (1) C16- H19	BD*(2) C4- C5	0.7

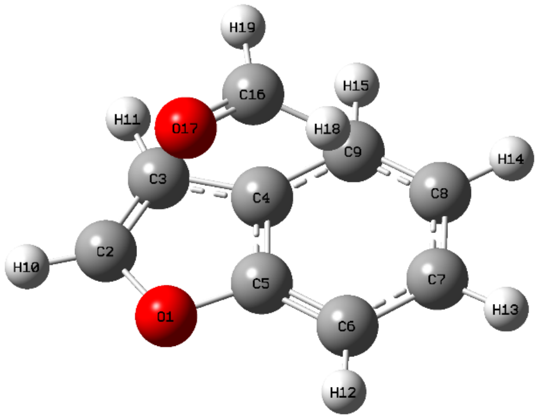

Table S1.3.4. NBO results for the Isomer ***IV*** of the BZF-FA complex.

NBO Donor	NBO Acceptor	E(2) [kJ·mol^{-1}]
BD (2) C2-C3	BD*(1) C16-H19	0.7
BD (2) C4-C5	BD*(1) C16-O17	2.3
BD (2) C4-C5	BD*(2) C16-O17	0.3
BD (2) C6-C7	BD*(1) C16-O17	0.9
BD (2) C8-C9	BD*(1) C16-H18	0.9
BD (1) C16-O17	BD*(2) C4-C5	0.3

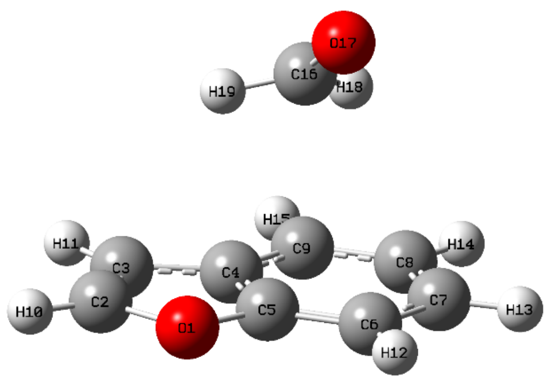

Table S1.3.5. NBO results for the Isomer ***V*** of the BZF-FA complex.

NBO Donor	NBO Acceptor	E(2) [kJ·mol^{-1}]
BD (2) C4-C5	BD*(1) C16- H19	0.4
LP (2) O17	BD*(1) C2-C3	0.3
LP (2) O17	BD*(2) C2-C3	0.4
BD (1) C16-O17	BD*(2) C8-C9	0.3
BD (2) C16-O17	RY (2) C3	0.3
BD (2) C16-O17	RY (1) H15	0.2
BD (1) C16-H19	BD*(2) C2-C3	0.5
BD (1) C16-H19	BD*(2) C4-C5	0.8
BD (1) C16-H19	BD*(2) C8-C9	0.4

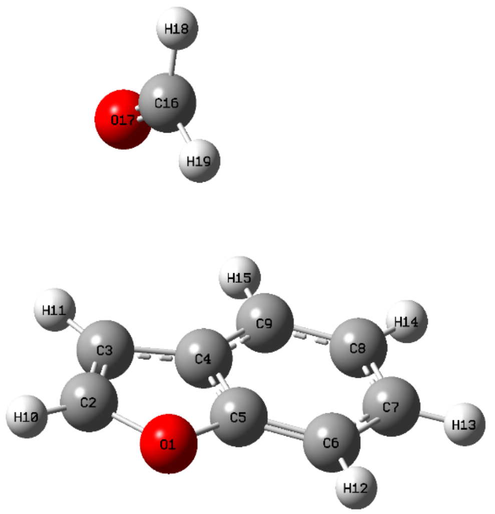

Table S1.3.6. NBO results for the Isomer ***VI*** of the BZF-FA complex.

NBO Donor	NBO Acceptor	E(2) [kJ·mol^{-1}]
BD (2) C2-C3	BD*(1) C16-H19	0.3
BD (1) C4-C5	RY (3) C16	0.3
BD (2) C4-C5	BD*(1) C16-O17	5.7
BD (2) C8-C9	BD*(1) C16-O17	0.3
BD (2) C16-O17	RY (3) C6	0.3
BD (1) C16-H18	BD*(2) C4-C5	0.8
BD (1) C16-H18	BD*(2) C8-C9	0.3

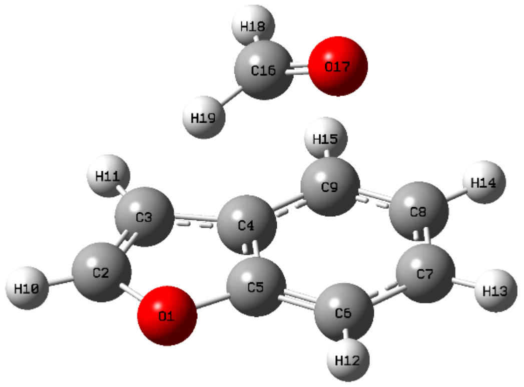

Table S1.3.7. NBO results for the Isomer ***VII*** of the BZF-FA complex.

NBO Donor	NBO Acceptor	E(2) [kJ·mol^{-1}]
LP (1) O1	BD*(1) C16-H18	2.1
LP (1) O1	RY (1) H18	0.3
BD (1) C2-H10	BD*(2) C16-O17	0.2
LP (1) O17	BD*(1) C2-H10	1.8
LP (2) O17	BD*(1) C2-C3	0.4
LP (2) O17	BD*(1) C2-H10	4.1
BD (2) C16-O17	RY (1) C2	0.4
BD (2) C16-O17	RY (3) C2	0.2

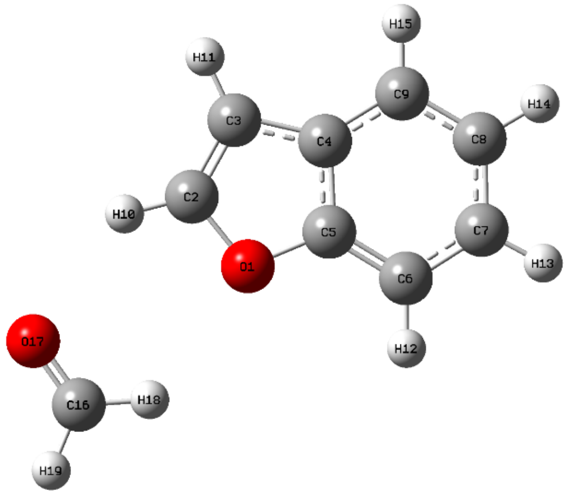

Table S1.3.8. NBO results for the Isomer ***VIII*** of the BZF-FA complex.

NBO Donor	NBO Acceptor	E(2) [kJ·mol^{-1}]
BD (2) C4-C5	BD*(1) C16-H19	0.8
BD (1) C6-C7	RY (3) C16	0.3
BD (2) C6-C7	BD*(1) C16-O17	3.8
BD (1) C7-C8	RY (3) C16	0.2
BD (2) C8-C9	BD*(2) C16-O17	0.3
BD (1) C16-H18	BD*(2) C6-C7	0.3
BD (1) C16-H18	BD*(2) C8-C9	0.3

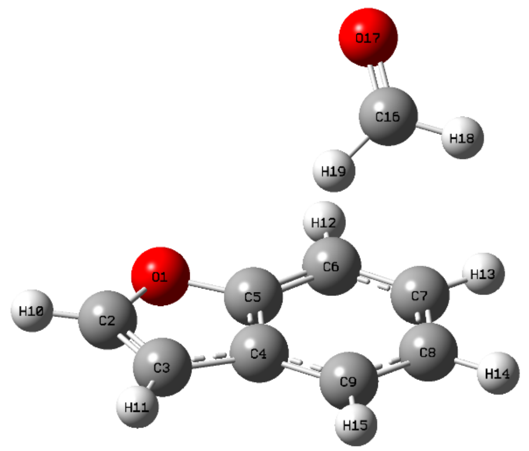

Table S1.3.9. NBO results for the Isomer ***IX*** of the BZF-FA complex.

NBO Donor	NBO Acceptor	E(2) [kJ·mol^{-1}]
LP (1) O1	BD*(1) C16-O17	0.4
LP (2) O1	BD*(1) C16-O17	2.5
BD (1) O1-C2	RY (3) C16	0.3
BD (1) O1-C5	RY (3) C16	0.2
BD (2) C2-C3	BD*(1) C16-O17	0.4
BD (2) C4-C5	BD*(1) C16-O17	0.4
BD (2) C4- C5	BD*(1) C16-H18	0.6
BD (1) C16-O17	BD*(2) C2-C3	0.4
BD (1) C16-H18	BD*(2) C4-C5	0.3
BD (1) C16-H19	BD*(2) C4-C5	0.4

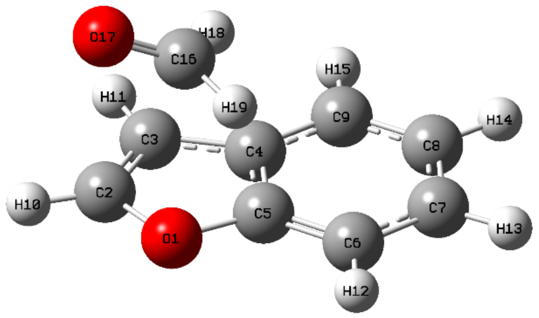

Table S1.3.10. NBO results for the Isomer ***X*** of the BZF-FA complex.

NBO Donor	NBO Acceptor	E(2) [kJ·mol^{-1}]
LP (2) O1	RY (3) O17	0.3
BD (1) C8-C9	RY (3) C16	0.2
BD (2) C8-C9	BD*(1) C16-O17	4.9
BD (2) C16-O17	RY (1) C4	0.3
BD (2) C16- O17	RY (3) C4	0.5
BD (2) C16-O17	RY (3) C6	0.3
BD (1) C16-H18	BD*(2) C8-C9	0.4
BD (1) C16-H19	BD*(2) C8-C9	0.5

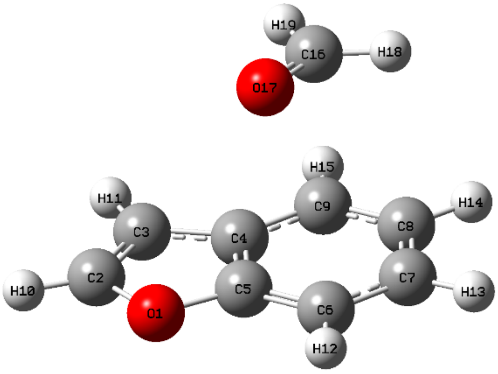

Table S1.3.11. NBO results for the Isomer ***XI*** of the BZF-FA complex.

NBO Donor	NBO Acceptor	E(2) [kJ·mol^{-1}]
LP (1) O1	BD*(1) C16-O17	0.7
LP (1) O1	BD*(2) C16-O17	3.5
LP (1) O1	RY (1) H18	0.3
LP (1) O1	RY (1) H19	0.3
BD (1) O1-C5	RY (3) C16	0.7
BD (1) C2-H10	BD*(2) C16-O17	0.4
LP (1) O17	BD*(1) C2-H10	0.8
BD (1) C16-O17	RY (1) C2	0.4
BD (1) C16-O17	RY (3) C2	0.3
BD (1) C16-O17	RY (1) H10	0.3
BD (2) C16-O17	BD*(1) O1-C2	0.3
BD (2) C16-O17	BD*(1) O1-C5	0.5
BD (2) C16-O17	BD*(1) C2-C3	0.5
BD (2) C16- O17	BD*(1) C2-H10	0.6

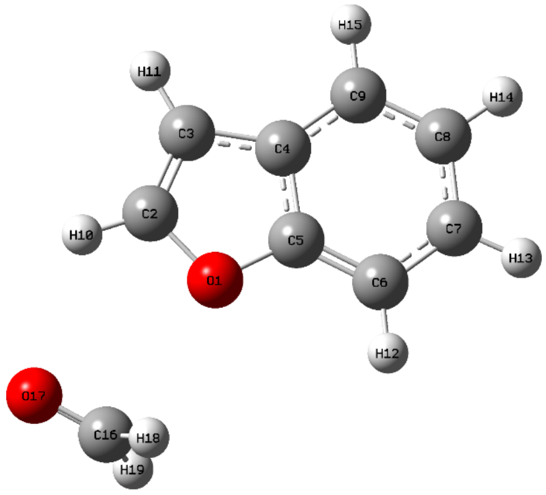

Table S1.3.12. NBO results for the Isomer ***XII*** of the BZF-FA complex.

NBO Donor	NBO Acceptor	E(2) [kJ·mol^{-1}]
BD (1) C6-C7	RY (3) C16	0.2
BD (2) C6-C7	BD*(1) C16-O17	3.8
BD (2) C16-O17	RY (1) C4	0.3
BD (2) C16-O17	RY (3) C4	0.5
BD (2) C16-O17	RY (4) C5	0.2
BD (1) C16-H18	BD*(2) C6-C7	0.4
BD (1) C16-H18	BD*(2) C8-C9	0.2
BD (1) C16-H19	BD*(2) C6-C7	0.3

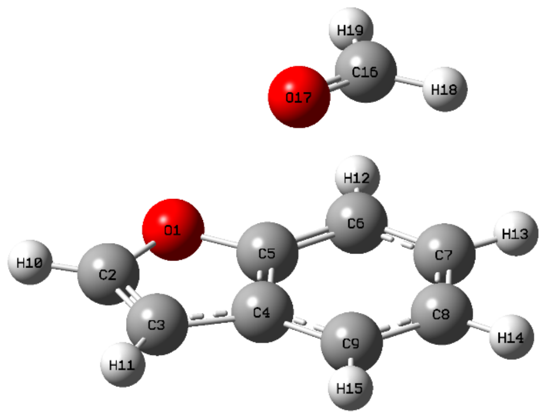

Table S1.3.13. NBO results for the Isomer ***XIII*** of the BZF-FA complex.

NBO Donor	NBO Acceptor	E(2) [kJ·mol^{-1}]
LP (1) O1	BD*(1) C16-H18	3.5
LP (1) O1	RY (1) H18	0.5
BD (1) C6-H12	BD*(2) C16-O17	0.2
LP (1) O17	BD*(1) C6-H12	1.8
LP (1) O17	RY (8) C6	0.3
LP (2) O17	BD*(1) C6-H12	5.1
LP (2) O17	RY (1) H12	0.2
LP (2) O17	RY (6) H12	0.3
BD (2) C16-O17	RY (2) C6	0.3
BD (2) C16-O17	RY (1) H12	0.4

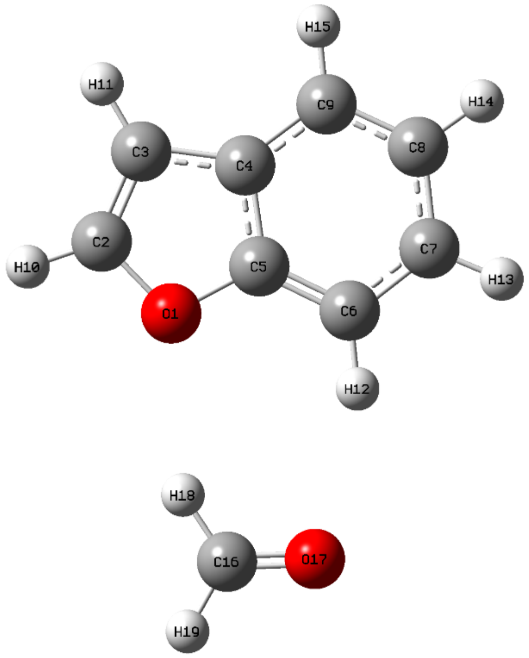

Table S1.3.14. NBO results for the Isomer ***XIV*** of the BZF-FA complex.

NBO Donor	NBO Acceptor	E(2) [kJ·mol^{-1}]
LP (1) O1	BD*(1) C16-O17	2.7
LP (1) O1	RY (3) C16	0.3
LP (2) O1	BD*(1) C16-O17	1.3
LP (2) O1	RY (2) H18	0.2
LP (1) O17	BD*(1) C6-H12	0.5
LP (2) O17	BD*(1) C6-H12	1.3
BD (1) C16-O17	BD*(1) C6-C7	0.2
BD (1) C16-O17	BD*(1) C6-H12	0.5
BD (2) C16-O17	RY (1) C6	0.2
BD (2) C16-O17	RY (2) C6	0.3
BD (2) C16-O17	RY (1) H12	0.5
BD (1) C16-H18	BD*(2) C6-C7	0.3

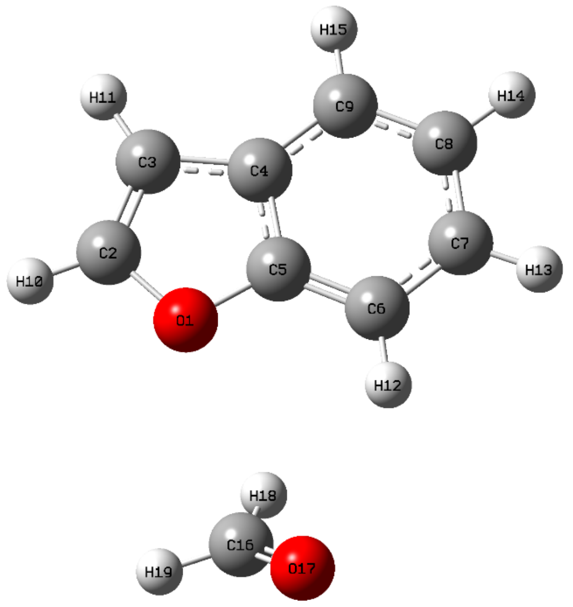

S1.4. Natural Energy Decomposition Analysis (NEDA).

For the fourteen low-lying minima, the Natural Energy Decomposition Analysis (NEDA) has been performed, using the NBO7 program[11] interfacted to Gaussian16,[10] at the B3LYP-D3(BJ)/maug-cc-pVTZ-*d*H level of theory,[2,12,13] on top of the corresponding CP-jB2 geometries. The obtained results are reported in Table S1.4.1 and graphically shown in Figure SF1.4.1. For the isomer ***I***, the NEDA results obtained on top of the CP-jB2 and semi-experimental equilibrium (r_e^{SE}, see section S3) structures are collected in Table S1.4.2 and depicted in Figure SF1.4.2 for comparison purposes.

Table S1.4.1. NEDA results (in kJ·mol^{-1}) on top of the CP-jB2 geometries.

**ISOMER**	ELECTRICAL	CHARGE TRANSFER	CORE	TOTAL[a]
I	-22.6	-18.3	24.2	-16.7
II	-25.1	-22.6	31.3	-16.4
III	-24.7	-21.9	30.3	-16.4
IV	-20.8	-17.9	22.9	-15.9
V	-22.8	-15.5	23.9	-14.3
VI	-22.0	-20.8	28.1	-14.6
VII	-24.8	-19.0	30.2	-13.6
VIII	-20.3	-15.7	21.6	-14.5
IX	-20.1	-18.5	25.0	-13.7
X	-24.5	-21.7	32.4	-13.8
XI	-28.7	-20.0	36.0	-12.7
XII	-22.0	-19.2	27.7	-13.5
XIII	-22.9	-22.4	32.8	-12.6
XIV	-24.2	-20.1	32.2	-12.2

[a] Each contribution (including the total energy one) is rounded to its closest decimal value according to the obtained data. The sum of rounded terms of each isomer can, therefore, differ from the reported total energy (maximum discrepancy 0.1 kJ·mol^{-1}).

Table S1.4.2. NEDA results (in kJ·mol^{-1}) for the isomer ***I*** on top of the CP-jB2 and semi-experimental equilibrium (r_e^{SE}) structures.

**ISOMER *I***	ELECTRICAL	CHARGE TRANSFER	CORE	TOTAL
CP-jB2	-22.6	-18.3	24.2	-16.7
r_e^{SE}	-21.3	-17.6	22.3	-16.6

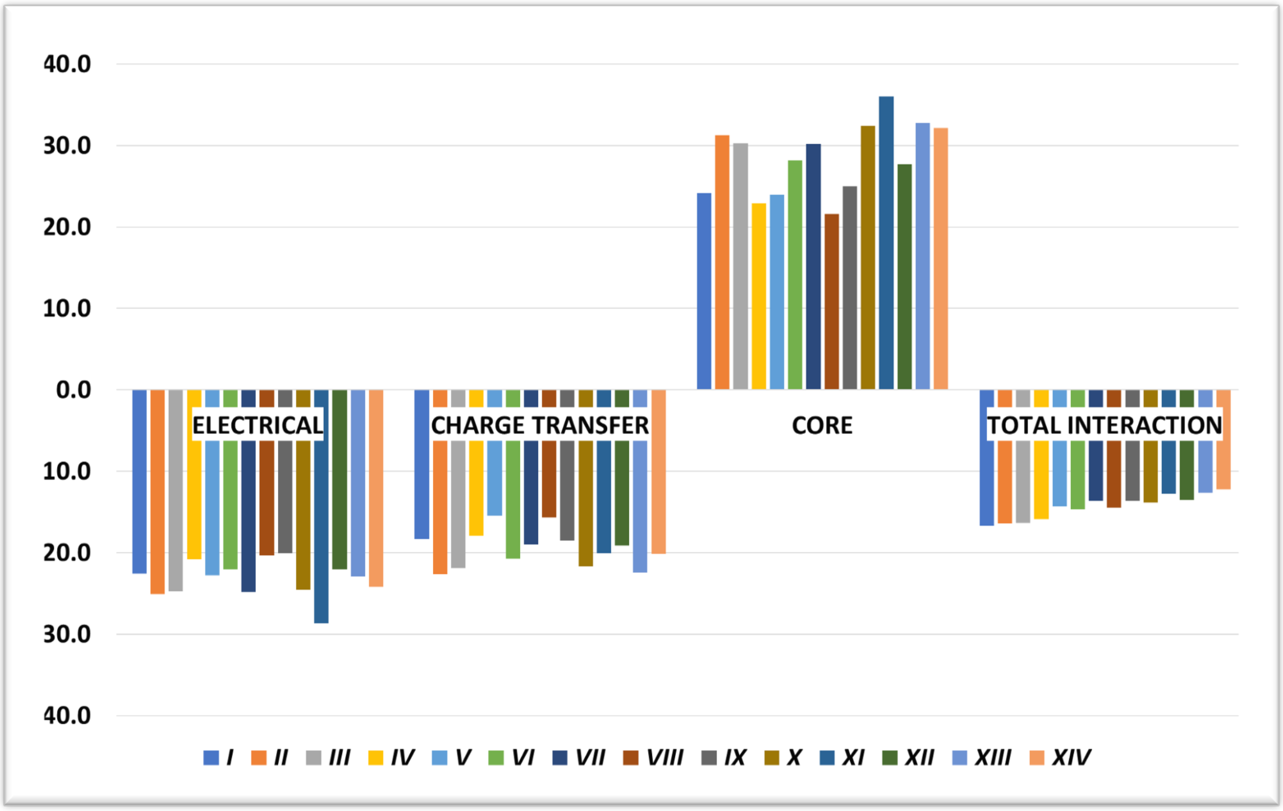

Figure SF1.4.1. Graphical representation of the NEDA results on top of the CP-jB2 geometries. The color legend for the different isomers is provided here above.

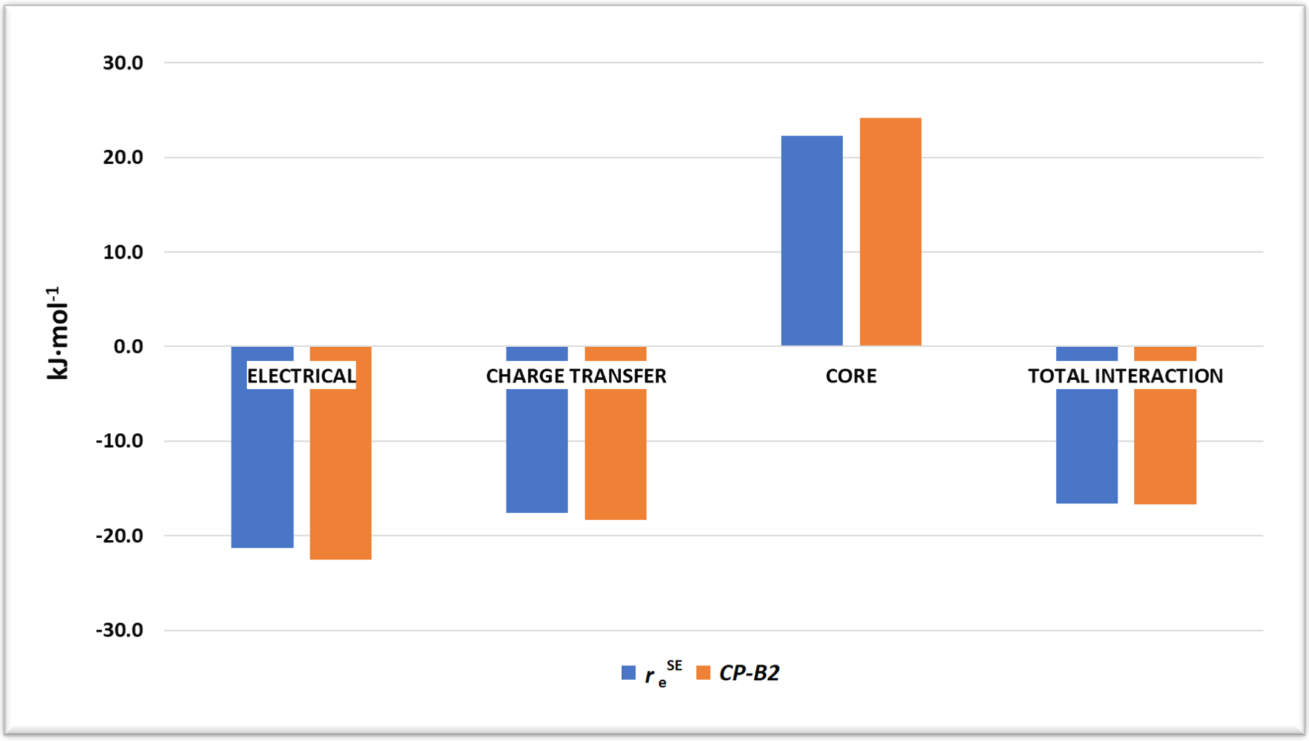

Figure SF1.4.2. Graphical representation of the NEDA results for the isomer ***I*** on top of the CP-jB2 and semi-experimental equilibrium (r_e^{SE}) structures.

S1.5. Symmetry Adapted Perturbation Theory (SAPT) Analysis.

Using the CP-jB2 optimzed geometries of the fourteen low-lying minima, the interaction energy decomposition analysis has been carried out employing the symmetry adapted perturbation theory (SAPT)[14] approach. In detail, the analysis has been performed at the "gold-standard" SAPT2+(3)δMP2/aug-cc-pVTZ level of theory (see ref.14b for details) employing the PSI4 program package.[15] The total interaction energy (E_{tot}) and its decomposition into the electrostatic (E_{elec}), dispersion (E_{disp}), induction (E_{ind}), and exchange-repulsion (E_{ex}) terms are reported in Table S1.5.1.

Table S1.5.1. Results of the SAPT2+(3)δmp2/aug-cc-pVTZ calculations for the BZF−FA clusters. Values in kJ·mol^{-1}.

Isomer	E_{elec}	E_{ex}	E_{ind}	E_{disp}	E_{tot}[a]
I	−11.8(33%)[b]	19.7	−3.4(9%)	−20.9(58%)	−16.3
II	−11.4(29%)	23.0	−4.8(12%)	−23.1(59%)	−16.3
III	−11.4(29%)	22.9	−4.5(12%)	−23.0(59%)	−16.0
IV	−10.9(32%)	18.9	−3.2(9%)	−20.2(59%)	−15.5
V	−12.2(41%)	15.7	−2.1(7%)	−15.7(52%)	−14.3
VI	−8.8(25%)	21.0	−4.3(12%)	−22.2(63%)	−14.3
VII	−15.8(51%)	16.4	−3.0(10%)	−11.9(39%)	−14.3
VIII	−10.5(34%)	17.7	−3.1(10%)	−17.5(56%)	−13.5
IX	−9.5(32%)	15.4	−2.4(8%)	−17.6(60%)	−14.1
X	−9.0(25%)	22.5	−4.7(13%)	−22.0(62%)	−13.2
XI	−14.4(46%)	17.7	−3.4(11%)	−13.4(43%)	−13.6
XII	−8.3(25%)	19.8	−3.9(12%)	−20.6(63%)	−13.1
XIII	−14.7(48%)	18.0	−3.3(11%)	−12.9(42%)	−12.9
XIV	−12.3(41%)	17.4	−2.7(9%)	−15.0(50%)	−12.6

[a] Each contribution (including the total energy one) is rounded to its closest decimal value according to the obtained data. The sum of rounded terms of each isomer can, therefore, differ from the reported total energy (maximum discrepancy 0.1 kJ·mol^{-1}).
[b]The values in parentheses represent the percent contribution of the total interaction energy.

S1.6. Analysis of non-covalent interactions in the PDB:3HPT YET 2.D ligand model.

We have investigated the role of π-π^* interactions in ruling the stabilization of intermolecular complexes in the gas phase and in solid state by comparing the NEDA and SAPT results for the BZF-FA complex and the BZF derivative of the YET 2.D ligand (PDB:3HPT).[16,17] In the latter case, we have selected the most representative portion of the crystal (Figure SF1.6.1), the chosen fragment being 2MeBZF-ACET (see Table S1.6.1). This consists of the BZF derivative, namely 2-methylbenzofuran, and the acetamide group of the peptic chain (peptide linkage between the TRP 215.D and GLY 216.D fragment). Table S1.6.1 collects the Cartesian coordinates taken from the PDB[16,17] together with those of the hydrogen atoms added to complete the valence of the terminal atoms of 2MeBZF-ACET. For 2MeBZF-ACET, the NBO, NEDA and SAPT analyses have been performed at the same levels of theory employed for the BZF-FA complex (see Tables S1.6.2 and S1.6.3).

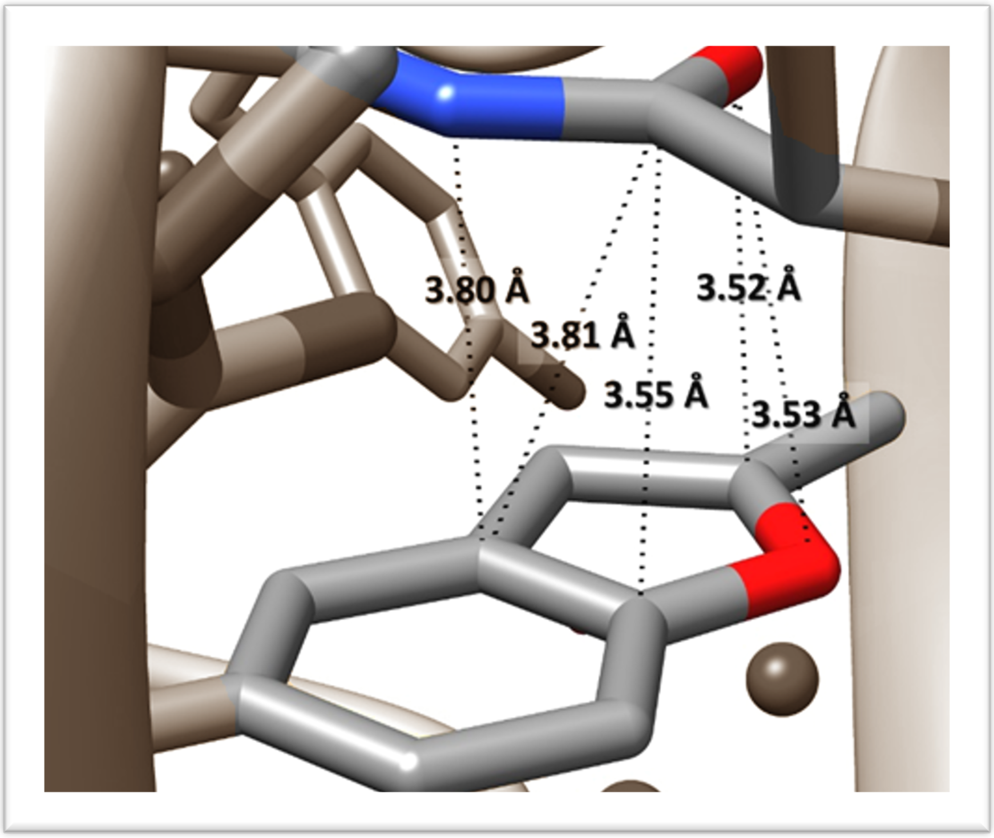

Figure SF1.6.1. Portion of the PDB:3HPT considered in the bond analyses. The Chimera 1.14[18] and GaussView softwares have been employed for graphical representations.

Table S1.6.1. Cartesian coordinates (Å) and schematic drawing of 2MeBZF-ACET within the crystal.

ATOM	X	Y	Z
C	2.282685	-1.742834	1.153684
C	1.728642	-1.765113	-0.267936
O	0.758406	-2.472842	-0.564997
H	2.309041	-0.701675	1.474579
N	2.359124	-0.967650	-1.130771
H	3.168432	-0.454064	-0.811629
H	1.636739	-2.320655	1.815402
H	3.290119	-2.158886	1.164367
H	2.034528	-0.879038	-2.083373
C	0.910236	2.743514	-0.536161
C	1.125124	2.439011	0.821211
C	-0.071450	2.052992	-1.261589
C	-0.812573	1.055738	-0.618609
C	-0.552137	0.775552	0.705774
C	0.402516	1.435230	1.462571
O	-1.400704	-0.259137	1.139680
C	-2.146964	-0.578452	0.041427
C	-3.188401	-1.651838	0.125977
C	-1.867652	0.186331	-1.111550
H	1.865459	2.995476	1.376744
H	-0.252733	2.287885	-2.300271
H	0.578182	1.183532	2.498600
H	-2.315752	0.138927	-2.093000
H	-3.657180	-1.627509	1.109583
H	-3.945136	-1.484622	-0.641351
H	-2.721803	-2.624142	-0.030108
H	1.500969	3.507787	-1.018477

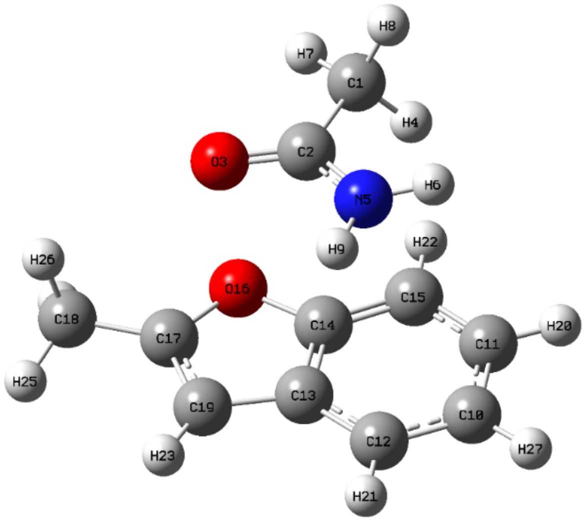

Table S1.6.2. NBO results for the 2MeBZF-ACET structure (in kJ·mol^{-1}).

NBO Donor	NBO Acceptor	E(2) [kJ·mol^{-1}]
BD (1) C1- H4	BD*(2) C11-C15	0.8
BD (2) C11- C15	BD*(1) C1-H4	1.2
BD (2) C13-C14	BD*(2) C2-O3	0.8

Table S1.6.3. Comparison of the NBO, NEDA and SAPT results for the Isomer ***I***, Isomer ***II*** and 2MeBZF-ACET structures. Values in kJ·mol^{-1}.

	NBO (C=C···C=O)	NEDA				SAPT				
	**E(2)**	**EL**[a]	**CT**[a]	**CO**[a]	**IE**[a]	**ELST**[a]	**IND**[a]	**DISP**[a]	**EXC**[a]	**TI**[a]
**Isomer *I***	4.3	-22.6	-18.3	24.2	-16.7	−11.8	−3.4	−20.9	19.7	−16.3
**Isomer *II***	4.1	-25.1	-22.6	31.3	-16.4	−11.4	−4.8	−23.1	23.0	−16.3
**Crystal Model**	0.8	-21.5	-18.9	21.4	-19.1	−6.5	−3.1	−25.4	15.4	−19.5

[a]The EL, CT, CO, IE, ELST, IND, DISP, EXC and TI labels stand for “electrical”, “charge transfer”, “core”, “electrostatics”, “induction”, “dispersion”, “exchange” and “total interaction”, as defined by the corresponding analysis methods. Each contribution (including the total/interaction energy terms) is rounded to its closest decimal value according to the obtained data. The sum of rounded terms of each isomer can, therefore, differ from the reported total energy (maximum discrepancy 0.1 kJ·mol^{-1}).

S2. Experimental details

S2.1. Description of the experimental setup and of the assignment procedure.

The rotational spectra were measured using a coaxially oriented beam-resonator (COBRA) type[19] jet Fourier transform microwave (FTMW) spectrometer at Chongqing University.[20] Spanning the range 2–20 GHz, it is operated with the FTMW++ set of programs.[21] The estimated uncertainty of the present frequency measurements is 3 kHz.

The samples of BZF and polyformaldehyde (PFA) were purchased from Sigma-Aldrich and used without further purification. In all measurements, helium was employed as carrier gas at a backing pressure of 3 bar. PFA and BZF were placed in two different reservoirs inserted into the gas-line. While BZF was kept at room temperature, PFA was instead heated to 355 K to shift the equilibrium from PFA oligomers of 8-100 units towards formaldehyde (FA) monomers. The resulting gas mixture of BZF and FA in helium was then expanded through a solenoid valve (General Valve, Series 9, nozzle orifice 0.5 mm) into the Fabry-Pérot cavity to generate the molecular complexes.

Each rotational transition appears as a doublet because of the instrumental Doppler effect arising from the jet expanding coaxially to the TEM_{00q}-mode of the Fabry-Perot-type resonator. Therefore, the measured frequencies were obtained as the arithmetic mean of the frequencies of the two Doppler components.

The initial spectral survey was obtained in the 8-10 GHz frequency range, aming at recording the μ_a-type transitions for the first four low-energy isomers. However, only one species was detected. After the initial assignment of the $5_{0,5} \leftarrow 4_{0,4}$, $5_{1,5} \leftarrow 4_{1,4}$, $6_{0,6} \leftarrow 5_{0,5}$ and $6_{1,6} \leftarrow 5_{1,5}$ μ_a-type transitions, a total of 204 rotational transitions have been successfully fitted by means of the Pickett's SPFIT program[22] using the Watson's *S*-reduced semi-rigid Hamiltonian in its III^l representation.[23]

Most of the transitions exhibit a very narrow doublet, split with 1:3 intensity ratio, with the corresponding states being labelled as $\upsilon = 0$ and $\upsilon = 1$ states, respectively. This intensity ratio is ascribable to the exchange of the hydrogen atoms (nuclear spin I=1/2) of FA due to rotation around its C_2 axis. A set of rotational parameters (rotational constants and all five quartic centrifugal distortion constants) was well determined for each state, resulting in a microwave RMS of 2.7 kHz.

The comparison of the experimentally determined rotational parameters (see Table S2.2.1a) with the corresponding computed equilibrum values (see Table S1.1.2) does not allow for an unambigous assignment of the observed spectrum to a specific isomer, but instead it reduces the suitable candidates to the isomers ***I*** and ***IV***, which have similar calculated equilibrium rotational constants. Furthermore, they are very close in energy, with the isomer ***I*** being slightly more stable. While proper account of the vibrational corrections strongly suggests that the observed species is the isomer ***I***, the final unbiased assignment was achieved by successfully collecting and fitting the rotational spectra of the 9 monosubstituted ^{13}C isotopologues and the (^{18}O)FA species. The rotational data for several isotopologues also allowed for a quantitative structural determination (see section S3).

The ^{13}C isotopologues have been observed in natural abundance. The presence of the (^{18}O)FA species has been enhanced in situ by adding a drop of $H_2{}^{18}O$ in the carrier gas pipeline. This introduces a small amount of $H_2{}^{18}O$ vaper into the gas mixture which induces an in-situ isotope exchange reaction for producing the (^{18}O)FA.[21] The ratio of BZF-(^{18}O)FA and BZF-(^{16}O)FA in the supersonic jet is about 1:6 as made evident by their line intensities. In the case of the BZF-(^{18}O)FA species, rotational transitions belonging to both $\upsilon = 0$ and $\upsilon = 1$ states were successfully fitted to yield precise rotational parameters, while for the ^{13}C isotopologues, the observation of only the strongest tunnelling splitting component ($\upsilon = 1$ state) allowed us to determine the spectroscopic parameters only for this state. In the fitting procedure, we held D_{JK}, D_K, d_1 and d_2 fixed at the corresponding values of the parent species for the $\upsilon = 1$ state. As done for the parent species, the spectra of all isotopologues have been fitted by means of the SPFIT program by Pickett[22] using the Watson's S-reduced semi-rigid Hamiltonian in III^l representation.[23]

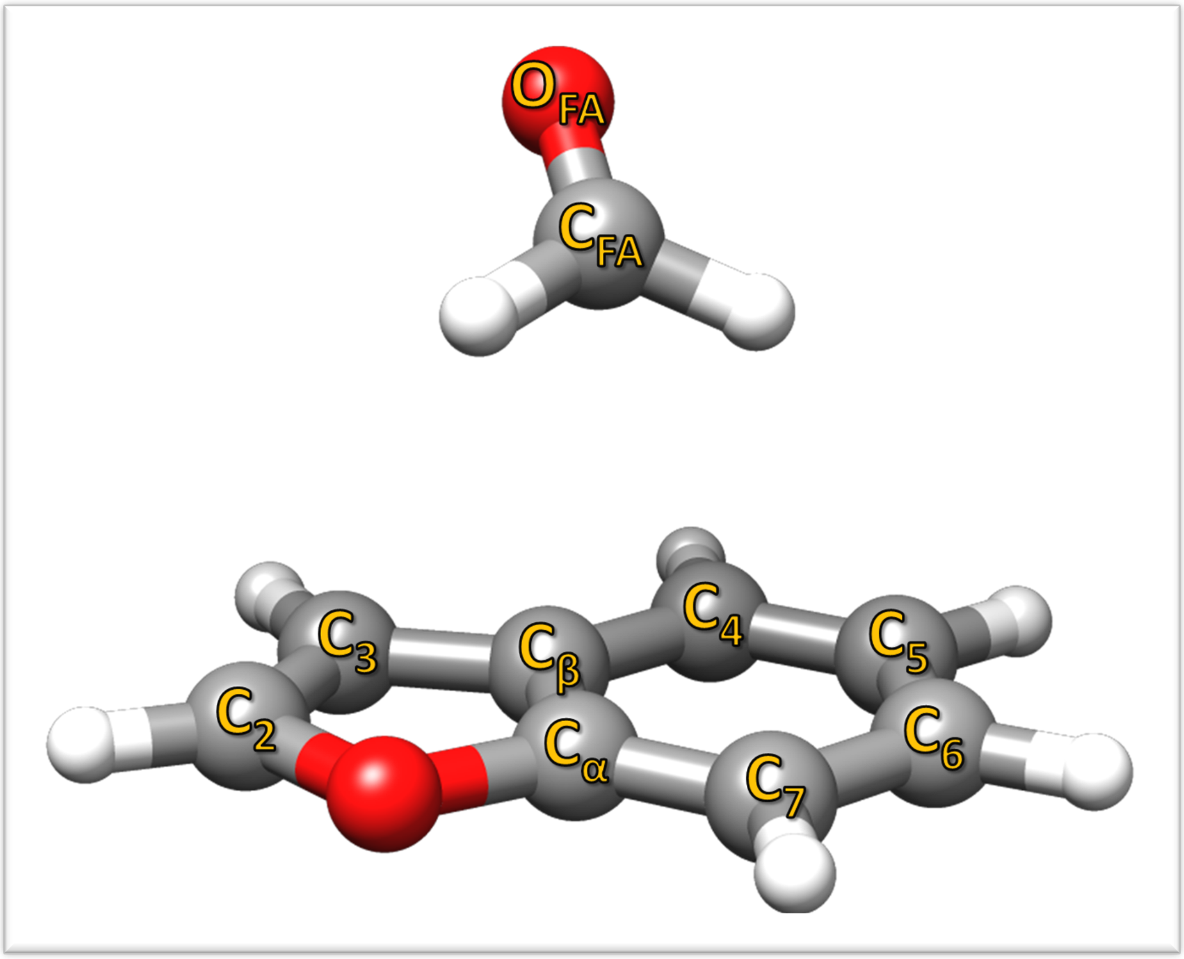

Figure SF2.1.1. Atom labels for the isomer ***I*** of the BZ-FA adduct

Spectral searches for other isomers (***II*** to ***XIV***) have been performed extensively, covering the 8.1-11.25 GHz frequency range (integrating 384 cycles in the 8.1-9.8 GHz range and integrating 512 cycles in the 9.0-11.25 GHz range). However, no rotational transitions belonging to these isomers could be assigned. This is in

agreement with the very low energy barriers ruling the relaxation to the most stable (and only observed in the supersonic expansion) structure (isomer ***I***) predicted by the computational investigation. In particular, for the hydrogen-bonded isomer ***VII***, additional survey scans in the 14.9-16.5 frequency range have been carried out using 0.4 MHz step and each step was averaged up to 1024 cycles. The non-detection of this isomer should be due to conformational relaxation or very weak signals.[25]
All fit results are reported in the tables below, followed by the measured transitions. The atomic numbering of the isomer ***I*** of the BZF-FA complex (which is used to label the isotopologues) is shown in Figure SF2.1.1.

S2.2. Experimental rotational transitions and fit results for the isomer *I* of the Benzofuran-Formaldehyde adduct

S2.2.1. The parent species.

Table S2.2.1a. Experimentally determined spectroscopic parameters (Watson's *S*-reduction, III^l representation) for the parent species of the isomer ***I*** of BZF-FA.

Parameter	Experiment	
	υ = 0	υ = 1
A_0 [MHz]	1180.9038(2)[a]	1180.9045(2)
B_0 [MHz]	1096.1994(2)	1096.2033(2)
C_0 [MHz]	788.2780(1)	788.2808(1)
D_J [kHz]	3.902(2)	3.903(2)
D_{JK} [kHz]	-4.826(6)	-4.822(6)
D_K [kHz]	1.829(4)	1.828(4)
d_1 [kHz]	-0.448(2)	-0.448(2)
d_2 [kHz]	0.9641(8)	0.9639(8)
N[b]	204	
σ[c] [kHz]	2.7	

[a] Standard errors in units of the last digit. [b] Number of the fitted lines; [c] Root-mean square error of the fit.

Table S2.2.1b. Numbered (*N*) experimental rotational transitions for the parent species of the isomer ***I*** of BZF-FA. Table reports the experimental frequencies (ν_{EXP}) and the residuals (observed - calculated differences; $\Delta\nu$).

N	J	K_a	K_c	v	J'	K_a'	K_c'	v'	ν_{EXP} [MHz]	$\Delta\nu$ [MHz]
1	3	1	3	0	2	1	2	0	5071.0350	-0.0021
2	3	1	3	1	2	1	2	1	5071.0534	-0.0001

3	3	0	3	0	2	0	2	0	5084.4732	-0.0020
4	3	0	3	1	2	0	2	1	5084.4951	0.0044
5	3	2	2	0	2	2	1	0	5653.0496	-0.0047
6	3	2	2	1	2	2	1	1	5653.0763	0.0022
7	3	1	2	0	2	1	1	0	5834.3696	-0.0023
8	3	1	2	1	2	1	1	1	5834.3896	0.0023
9	3	2	1	0	2	2	0	0	6221.7983	-0.0031
10	3	2	1	1	2	2	0	1	6221.8232	-0.0023
11	4	1	4	0	3	1	3	0	6653.0625	-0.0044
12	4	1	4	1	3	1	3	1	6653.0888	0.0010
13	4	0	4	0	3	0	3	0	6654.6198	-0.0058
14	4	0	4	1	3	0	3	1	6654.6463	0.0000
15	3	3	1	0	2	2	1	0	6966.8154	0.0006
16	3	3	1	1	2	2	1	1	6966.8221	-0.0010
17	3	3	0	0	2	2	1	0	7127.1212	0.0003
18	3	3	0	1	2	2	1	1	7127.1384	0.0045
19	4	2	3	0	3	2	2	0	7321.9193	-0.0046
20	4	2	3	1	3	2	2	1	7321.9461	-0.0002
21	4	1	3	0	3	1	2	0	7382.9407	-0.0019
22	4	1	3	1	3	1	2	1	7382.9613	0.0001
23	4	3	2	0	3	3	1	0	7805.5017	-0.0026
24	4	3	2	1	3	3	1	1	7805.5327	-0.0004
25	4	2	2	0	3	2	1	0	8110.3147	-0.0019
26	4	2	2	1	3	2	1	1	8110.3420	0.0023
27	5	0	5	0	4	1	4	0	8229.9541	0.0069
28	5	1	5	0	4	1	4	0	8229.9541	-0.0063
29	5	0	5	1	4	1	4	1	8229.9695	-0.0031
30	5	1	5	1	4	1	4	1	8229.9879	0.0021
31	5	0	5	0	4	0	4	0	8230.1076	0.0008
32	5	1	5	0	4	0	4	0	8230.1192	-0.0009
33	5	0	5	1	4	0	4	1	8230.1361	0.0039
34	5	1	5	1	4	0	4	1	8230.1361	-0.0093
35	4	3	1	0	3	3	0	0	8307.0390	-0.0007
36	4	3	1	1	3	3	0	1	8307.0746	-0.0013
37	5	1	4	0	4	2	3	0	8920.4381	-0.0019
38	5	1	4	1	4	2	3	1	8920.4645	-0.0014
39	5	2	4	0	4	2	3	0	8921.8628	0.0020
40	5	2	4	1	4	2	3	1	8921.8868	0.0003
41	5	1	4	0	4	1	3	0	8932.2507	0.0017
42	5	1	4	1	4	1	3	1	8932.2726	-0.0011
43	5	2	4	0	4	1	3	0	8933.6677	-0.0021
44	5	2	4	1	4	1	3	1	8933.6934	-0.0008
45	4	3	1	0	3	2	1	0	8973.9764	-0.0063
46	4	3	1	1	3	2	1	1	8974.0042	-0.0003
47	4	2	2	0	3	1	2	0	8990.2679	0.0000
48	4	2	2	1	3	1	2	1	8990.2931	-0.0004
49	4	1	3	0	3	0	3	0	9071.7132	0.0058
50	4	1	3	1	3	0	3	1	9071.7278	-0.0005
51	4	2	3	0	3	1	3	0	9081.7904	-0.0077
52	4	2	3	1	3	1	3	1	9081.8184	0.0005
53	4	3	2	0	3	2	2	0	9119.2561	-0.0087
54	4	3	2	1	3	2	2	1	9119.2832	0.0011
55	4	4	1	0	3	3	0	0	9142.6364	0.0036
56	4	4	1	1	3	3	0	1	9142.6364	-0.0018
57	4	4	0	0	3	3	0	0	9237.0325	-0.0006
58	4	4	0	1	3	3	0	1	9237.0428	-0.0001
59	4	4	1	0	3	3	1	0	9302.9372	-0.0018
60	4	4	1	1	3	3	1	1	9302.9485	-0.0004

61	4	4	0	0	3	3	1	0	9397.3367	-0.0025
62	4	4	0	1	3	3	1	1	9397.3518	-0.0019
63	5	3	3	0	4	3	2	0	9549.7913	0.0051
64	5	3	3	1	4	3	2	1	9549.8150	-0.0010
65	5	2	3	0	4	2	2	0	9706.1295	-0.0082
66	5	2	3	1	4	2	2	1	9706.1607	0.0019
67	4	3	1	0	3	2	2	0	9781.1076	0.0013
68	4	3	1	1	3	2	2	1	9781.1340	-0.0017
69	6	1	6	0	5	1	5	0	9806.1654	-0.0003
70	6	0	6	0	5	1	5	0	9806.1654	0.0008
71	6	0	6	0	5	0	5	0	9806.1786	0.0007
72	6	1	6	0	5	0	5	0	9806.1786	-0.0003
73	6	1	6	1	5	1	5	1	9806.1949	-0.0001
74	6	0	6	1	5	1	5	1	9806.1949	0.0009
75	6	1	6	1	5	0	5	1	9806.2090	0.0007
76	6	0	6	1	5	0	5	1	9806.2090	0.0018
77	5	4	2	0	4	4	1	0	9919.6061	0.0006
78	5	4	2	1	4	4	1	1	9919.6449	0.0005
79	5	4	1	0	4	4	0	0	10326.6519	-0.0034
80	5	4	1	1	4	4	0	1	10326.7054	-0.0001
81	5	3	2	0	4	3	1	0	10336.5866	0.0011
82	5	3	2	1	4	3	1	1	10336.6186	-0.0007
83	6	1	5	0	5	2	4	0	10501.3308	0.0043
84	6	1	5	1	5	2	4	1	10501.3559	-0.0002
85	6	2	5	0	5	2	4	0	10501.4746	0.0036
86	6	2	5	1	5	2	4	1	10501.4962	-0.0044
87	6	1	5	0	5	1	4	0	10502.7488	0.0015
88	6	1	5	1	5	1	4	1	10502.7764	-0.0003
89	5	4	2	0	4	3	1	0	10755.1983	-0.0004
90	5	4	2	1	4	3	1	1	10755.2044	-0.0022
91	6	2	4	0	5	3	3	0	11177.8370	0.0012
92	6	2	4	1	5	3	3	1	11177.8673	-0.0005
93	6	3	4	0	5	3	3	0	11184.7950	0.0011
94	6	3	4	1	5	3	3	1	11184.8246	-0.0003
95	5	3	2	0	4	2	2	0	11200.2538	0.0022
96	5	3	2	1	4	2	2	1	11200.2843	0.0002
97	6	2	4	0	5	2	3	0	11223.3117	0.0028
98	6	2	4	1	5	2	3	1	11223.3359	-0.0002
99	6	3	4	0	5	2	3	0	11230.2689	0.0018
100	6	3	4	1	5	2	3	1	11230.2945	0.0013
101	5	4	1	0	4	3	1	0	11256.6479	-0.0007
102	5	4	1	1	4	3	1	1	11256.6719	-0.0006
103	5	2	3	0	4	1	3	0	11313.4676	0.0046
104	5	2	3	1	4	1	3	1	11313.4910	-0.0002
105	5	3	3	0	4	2	3	0	11347.1330	0.0059
106	5	3	3	1	4	2	3	1	11347.1538	0.0021
107	5	1	4	0	4	0	4	0	11349.3360	0.0051
108	5	1	4	1	4	0	4	1	11349.3561	0.0005
109	5	2	4	0	4	1	4	0	11350.5909	-0.0011
110	5	2	4	1	4	1	4	1	11350.6161	-0.0005
111	7	0	7	0	6	0	6	0	11382.1938	-0.0008
112	7	1	7	0	6	1	6	0	11382.1938	0.0002
113	7	0	7	1	6	0	6	1	11382.2270	-0.0003
114	7	1	7	1	6	1	6	1	11382.2270	0.0006
115	5	4	2	0	4	3	2	0	11417.0375	-0.0027
116	5	4	2	1	4	3	2	1	11417.0595	-0.0008
117	5	5	1	0	4	4	0	0	11552.5365	-0.0045
118	5	5	1	1	4	4	0	1	11552.5365	-0.0110

119	5	5	0	0	4	4	0	0	11602.5426	0.0026
120	5	5	0	1	4	4	0	1	11602.5495	-0.0005
121	5	5	1	0	4	4	1	0	11646.9417	0.0005
122	5	5	1	1	4	4	1	1	11646.9509	-0.0014
123	5	5	0	0	4	4	1	0	11696.9377	-0.0025
124	5	5	0	1	4	4	1	1	11696.9531	-0.0017
125	6	4	3	0	5	4	2	0	11747.6048	0.0022
126	6	4	3	1	5	4	2	1	11747.6420	0.0008
127	6	3	3	0	5	3	2	0	12038.8989	-0.0013
128	6	3	3	1	5	3	2	1	12038.9264	0.0004
129	7	2	6	0	6	2	5	0	12077.5658	0.0078
130	7	2	6	1	6	2	5	1	12077.5865	-0.0046
131	7	1	6	0	6	1	5	0	12077.6961	0.0068
132	7	1	6	1	6	1	5	1	12077.7274	0.0049
133	6	4	3	0	5	3	2	0	12166.2162	0.0004
134	6	4	3	1	5	3	2	1	12166.2324	0.0039
135	6	4	2	0	5	4	1	0	12500.2449	-0.0020
136	6	4	2	1	5	4	1	1	12500.2958	0.0016
137	7	3	5	0	6	3	4	0	12772.7848	0.0004
138	7	3	5	1	6	3	4	1	12772.8173	-0.0007
139	7	2	5	0	6	2	4	0	12778.8901	0.0028
140	7	2	5	1	6	2	4	1	12778.9215	0.0014
141	8	0	8	0	7	0	7	0	12958.0730	0.0068
142	8	1	8	0	7	1	7	0	12958.0730	0.0069
143	8	0	8	1	7	0	7	1	12958.1016	0.0000
144	8	1	8	1	7	1	7	1	12958.1016	0.0001
145	6	5	2	0	5	4	1	0	13224.3273	-0.0011
146	6	5	2	1	5	4	1	1	13224.3344	-0.0001
147	6	4	2	0	5	3	2	0	13420.3106	0.0006
148	6	4	2	1	5	3	2	1	13420.3477	0.0003
149	7	4	4	0	6	4	3	0	13435.9927	0.0042
150	7	4	4	1	6	4	3	1	13436.0255	-0.0003
151	6	3	3	0	5	2	3	0	13533.0144	0.0002
152	6	3	3	1	5	2	3	1	13533.0522	0.0009
153	7	3	4	0	6	3	3	0	13538.6641	0.0003
154	7	3	4	1	6	3	3	1	13538.6916	0.0000
155	6	5	1	0	5	4	1	0	13570.2909	0.0066
156	6	5	1	1	5	4	1	1	13570.3087	0.0010
157	6	2	4	0	5	1	4	0	13604.5259	0.0030
158	6	2	4	1	5	1	4	1	13604.5554	0.0018
159	6	4	3	0	5	3	3	0	13614.8616	0.0050
160	6	4	3	1	5	3	3	1	13614.8857	0.0002
161	8	2	7	0	7	2	6	0	13653.1597	-0.0032
162	8	1	7	0	7	1	6	0	13653.1796	0.0047
163	8	2	7	1	7	2	6	1	13653.1967	-0.0023
164	8	1	7	1	7	1	6	1	13653.2126	0.0016
165	6	5	2	0	5	4	2	0	13725.7767	-0.0016
166	6	5	2	1	5	4	2	1	13725.7988	-0.0015
167	6	6	1	0	5	5	0	0	13947.3200	0.0078
168	6	6	1	1	5	5	0	1	13947.3200	-0.0005
169	6	6	0	0	5	5	0	0	13971.8722	-0.0019
170	6	6	0	1	5	5	0	1	13971.8855	0.0008
171	6	6	1	0	5	5	1	0	13997.3120	0.0008
172	6	6	1	1	5	5	1	1	13997.3246	0.0016
173	6	6	0	0	5	5	1	0	14021.8742	0.0011
174	6	6	0	1	5	5	1	1	14021.8879	0.0007
175	6	5	1	0	5	4	2	0	14071.7328	-0.0015
176	6	5	1	1	5	4	2	1	14071.7724	-0.0012

177	8	2	5	0	7	2	4	0	14349.3641	-0.0028
178	8	2	5	1	7	2	4	1	14349.4037	0.0001
179	7	4	3	0	6	4	2	0	14349.6260	-0.0018
180	7	4	3	1	6	4	2	1	14349.6608	-0.0019
181	8	2	6	0	7	2	5	0	14350.1289	-0.0023
182	8	2	6	1	7	2	5	1	14350.1682	0.0005
183	9	0	9	0	8	0	8	0	14533.7584	-0.0032
184	9	1	9	0	8	1	8	0	14533.7584	-0.0032
185	9	0	9	1	8	0	8	1	14533.7991	0.0003
186	9	1	9	1	8	1	8	1	14533.7991	0.0003
187	8	3	4	0	7	3	3	0	15042.6470	-0.0010
188	8	3	4	1	7	3	3	1	15042.6859	0.0000
189	8	3	5	0	7	3	4	0	15063.6630	0.0014
190	8	3	5	1	7	3	4	1	15063.6970	0.0004
191	9	1	8	0	8	1	7	0	15228.5970	-0.0070
192	9	2	8	0	8	2	7	0	15228.5970	-0.0060
193	9	1	8	1	8	1	7	1	15228.6416	-0.0006
194	9	2	8	1	8	2	7	1	15228.6416	0.0004
195	8	4	3	0	7	4	2	0	15667.9232	-0.0020
196	8	4	3	1	7	4	2	1	15667.9718	0.0016
197	8	4	4	0	7	4	3	0	15882.9506	-0.0008
198	8	4	4	1	7	4	3	1	15882.9776	-0.0016
199	9	3	7	0	8	3	6	0	15924.2669	-0.0029
200	9	3	7	1	8	3	6	1	15924.3092	0.0002
201	9	2	7	0	8	2	6	0	15924.3544	0.0023
202	9	2	7	1	8	2	6	1	15924.3935	0.0023
203	9	3	6	0	8	3	5	0	16625.1407	-0.0050
204	9	3	6	1	8	3	5	1	16625.1820	-0.0030

S2.2.2. The BZF-(^{18}O)FA isotopologue.

Table S2.2.2a. Experimentally determined spectroscopic parameters (Watson's *S*-reduction, *III*^{l} representation) for the BZF-(^{18}O)FA isotopologue of the isomer ***I*** of BZF-FA.

Parameter	Experiment	
	υ = 0	υ = 1
A_0 [MHz]	1179.608(2)[a]	1179.6120(7)
B_0 [MHz]	1047.2712(6)	1047.2738(4)
C_0 [MHz]	762.0013(3)	762.0043(2)
D_J [kHz]	3.95(4)	3.865(9)
D_{JK} [kHz]	-5.11(8)	-4.84(2)
D_K [kHz]	2.09(4)	1.90(1)
d_1 [kHz]	-0.55(3)	-0.571(7)
d_2 [kHz]	1.072(7)	1.067(3)
N[b]	77	
σ[c] [kHz]	3.1	

[a] Standard errors in units of the last digit. [b] Number of the fitted lines; [c] Root-mean square error of the fit.

Table S2.2.2b. Numbered (*N*) experimental rotational transitions for the BZF-(^{18}O)FA isotopologue of the isomer ***I*** of BZF-FA. Table reports the experimental frequencies (ν_{EXP}) and the residuals (observed - calculated differences; $\Delta\nu$).

N	J	K_a	K_c	v	J'	K_a'	K_c'	v'	ν_{EXP} [MHz]	$\Delta\nu$ [MHz]
1	3	2	2	0	2	2	1	0	5427.4200	-0.0062
2	3	2	2	1	2	2	1	1	5427.4411	-0.0019
3	3	1	2	0	2	1	1	0	5657.6112	-0.0035
4	3	1	2	1	2	1	1	1	5657.6356	0.0043
5	3	2	1	0	2	2	0	0	5922.1979	0.0012
6	3	2	1	1	2	2	0	1	5922.2150	0.0000
7	4	1	4	0	3	1	3	0	6438.6171	-0.0061
8	4	1	4	1	3	1	3	1	6438.6433	-0.0001
9	4	0	4	0	3	0	3	0	6444.1917	0.0004
10	4	0	4	1	3	0	3	1	6444.2134	0.0016
11	4	2	3	0	3	2	2	0	7072.1501	0.0056
12	4	2	3	1	3	2	2	1	7072.1635	-0.0004
13	4	1	3	0	3	1	2	0	7195.4277	-0.0025
14	4	1	3	1	3	1	2	1	7195.4528	0.0014
15	4	3	2	0	3	3	1	0	7463.5674	0.0026
16	4	3	2	1	3	3	1	1	7463.5895	-0.0014
17	4	2	2	0	3	2	1	0	7819.2579	0.0039
18	4	2	2	1	3	2	1	1	7819.2797	0.0015
19	4	3	1	0	3	3	0	0	7847.5513	-0.0076
20	4	3	1	1	3	3	0	1	7847.5850	-0.0021
21	5	1	5	0	4	1	4	0	7964.4546	-0.0029
22	5	1	5	1	4	1	4	1	7964.4814	0.0000
23	5	0	5	0	4	0	4	0	7965.2904	-0.0014

24	5	0	5	1	4	0	4	1	7965.3154	-0.0004
25	5	2	4	0	4	2	3	0	8641.9112	0.0035
26	5	2	4	1	4	2	3	1	8641.9317	0.0035
27	5	1	4	0	4	1	3	0	8677.0918	0.0011
28	5	1	4	1	4	1	3	1	8677.1107	-0.0022
29	4	4	0	1	3	3	0	1	9173.1371	0.0004
30	5	3	3	0	4	3	2	0	9197.0212	0.0067
31	5	3	3	1	4	3	2	1	9197.0424	0.0010
32	4	4	1	1	3	3	1	1	9228.8906	0.0009
33	5	4	2	0	4	4	1	0	9447.4012	0.0049
34	5	4	2	1	4	4	1	1	9447.4399	0.0028
35	5	2	3	0	4	2	2	0	9470.9599	0.0022
36	5	2	3	1	4	2	2	1	9470.9869	-0.0006
37	6	1	6	0	5	1	5	0	9488.3340	0.0028
38	6	1	6	1	5	1	5	1	9488.3570	-0.0012
39	6	0	6	0	5	0	5	0	9488.4483	0.0057
40	6	0	6	1	5	0	5	1	9488.4681	-0.0016
41	5	4	1	0	4	4	0	0	9698.4451	-0.0031
42	5	4	1	1	4	4	0	1	9698.4903	-0.0007
43	5	3	2	0	4	3	1	0	9892.1052	0.0035
44	5	3	2	1	4	3	1	1	9892.1389	0.0025
45	6	2	5	0	5	2	4	0	10177.1587	-0.0056
46	6	2	5	1	5	2	4	1	10177.1830	-0.0010
47	6	1	5	0	5	1	4	0	10184.1937	0.0014
48	6	1	5	1	5	1	4	1	10184.2109	-0.0019
49	6	3	4	0	5	3	3	0	10825.7797	-0.0027
50	6	3	4	1	5	3	3	1	10825.8077	-0.0001
51	6	2	4	0	5	2	3	0	10944.1038	-0.0038
52	6	2	4	1	5	2	3	1	10944.1389	0.0006
53	7	1	7	0	6	1	6	0	11011.8106	0.0024
54	7	1	7	1	6	1	6	1	11011.8319	-0.0059
55	7	0	7	0	6	0	6	0	11011.8319	0.0099
56	7	0	7	1	6	0	6	1	11011.8546	0.0030
57	5	1	4	1	4	0	4	1	11045.2766	-0.0020
58	5	5	0	1	4	4	0	1	11544.5408	0.0026
59	5	5	1	1	4	4	1	1	11571.7225	-0.0028
60	7	2	6	0	6	2	5	0	11702.4026	-0.0002
61	7	2	6	1	6	2	5	1	11702.4142	-0.0053
62	7	1	6	0	6	1	5	0	11703.5633	-0.0059
63	6	3	3	0	5	3	2	0	11703.5836	-0.0048
64	7	1	6	1	6	1	5	1	11703.5836	-0.0026
65	6	3	3	1	5	3	2	1	11703.6283	-0.0020
66	7	2	5	1	6	2	4	1	12417.5720	-0.0014
67	8	0	8	0	7	0	7	0	12535.1020	-0.0029
68	8	1	8	0	7	1	7	0	12535.1020	-0.0013
69	8	0	8	1	7	0	7	1	12535.1343	-0.0023
70	8	1	8	1	7	1	7	1	12535.1343	-0.0007
71	8	2	7	1	7	2	6	1	13225.5915	0.0041
72	8	1	7	0	7	1	6	0	13225.7536	0.0052
73	8	1	7	1	7	1	6	1	13225.7641	0.0040
74	9	0	9	0	8	0	8	0	14058.2180	-0.0017
75	9	1	9	0	8	1	8	0	14058.2180	-0.0015
76	9	0	9	1	8	0	8	1	14058.2517	-0.0014
77	9	1	9	1	8	1	8	1	14058.2517	-0.0012

S2.2.3. The ($^{13}C_2$)BZF-FA isotopologue.

Table S2.2.3a. Experimentally determined spectroscopic parameters (Watson's *S*-reduction, *III*^{l} representation) for the υ = 1 state of the ($^{13}C_2$)BZF-FA isotopologue of the isomer ***I*** of BZF-FA.

Parameter	**Experiment**
	υ = 1
A_0 [MHz]	1165.146(6)[a]
B_0 [MHz]	1095.96(1)
C_0 [MHz]	781.1750(8)
D_J [kHz]	3.939(8)
D_{JK} [kHz]	[-4.822(6)][b]
D_K [kHz]	[1.828(4)]
d_1 [kHz]	[-0.448(2)]
d_2 [kHz]	[0.9639(8)]
N[c]	10
σ[d] [kHz]	4.0

[a] Standard errors in units of the last digit. [b] Values in brackets are fixed to the corresponding υ = 1 state values of the parent species. [c] Number of the fitted lines; [d] Root-mean square error of the fit.

Table S2.2.3b. Numbered (*N*) experimental rotational transitions for the υ = 1 state of the ($^{13}C_2$)BZF-FA isotopologue of the isomer ***I*** of BZF-FA. Table reports the experimental frequencies (ν_{EXP}) and the residuals (observed - calculated differences; $\Delta\nu$).

N	J	K_a	K_c	J'	K_a'	K_c'	ν_{EXP} [MHz]	$\Delta\nu$ [MHz]
1	4	1	4	3	1	3	6596.4662	0.0042
2	4	0	4	3	0	3	6597.3255	-0.0025
3	4	1	3	3	1	2	7316.2028	0.0004
4	5	0	5	4	0	4	8158.9421	0.0048
5	5	1	5	4	1	4	8158.8726	0.0011
6	5	1	4	4	1	3	8858.1212	-0.0007
7	6	1	6	5	1	5	9720.8273	-0.0077
8	6	0	6	5	0	5	9720.8358	-0.0036
9	7	1	7	6	1	6	11282.6457	0.0050
10	7	0	7	6	0	6	11282.6457	0.0047

S2.2.4. The ($^{13}C_3$)BZF-FA isotopologue.

Table S2.2.4a. Experimentally determined spectroscopic parameters (Watson's *S*-reduction, *III*[l] representation) for the υ = 1 state of the ($^{13}C_3$)BZF-FA isotopologue of the isomer ***I*** of BZF-FA.

Parameter	**Experiment**
	υ = 1
A_0 [MHz]	1169.799(4)
B_0 [MHz]	1092.838(6)
C_0 [MHz]	785.1122(5)
D_J [kHz]	3.893(5)
D_{JK} [kHz]	[-4.822(6)][b]
D_K [kHz]	[1.828(4)]
d_1 [kHz]	[-0.448(2)]
d_2 [kHz]	[0.9639(8)]
N[c]	10
σ[d] [kHz]	2.3

[a] Standard errors in units of the last digit. [b] Values in brackets are fixed to the corresponding υ = 1 state values of the parent species. [c] Number of the fitted lines; [d] Root-mean square error of the fit.

Table S2.2.4b. Numbered (*N*) experimental rotational transitions for the υ = 1 state of the ($^{13}C_3$)BZF-FA isotopologue of the isomer ***I*** of BZF-FA. Table reports the experimental frequencies (ν_{EXP}) and residuals (observed - calculated differences; $\Delta\nu$).

N	J	K_a	K_c	J'	K_a'	K_c'	ν_{EXP} [MHz]	$\Delta\nu$ [MHz]
1	4	1	4	3	1	3	6624.2394	-0.0002
2	4	0	4	3	0	3	6625.4425	-0.0019
3	4	1	3	3	1	2	7341.9319	0.0000
4	5	1	5	4	1	4	8194.6659	-0.0003
5	5	0	5	4	0	4	8194.7706	0.0009
6	5	1	4	4	1	3	8888.1746	0.0002
7	6	1	6	5	1	5	9764.5268	-0.0032
8	6	0	6	5	0	5	9764.5433	0.0054
9	7	1	7	6	1	6	11334.2299	-0.0010
10	7	0	7	6	0	6	11334.2299	-0.0016

S2.2.5. The ($^{13}C_\alpha$)BZF-FA isotopologue.

Table S2.2.5a. Experimentally determined spectroscopic parameters (Watson's *S*-reduction, III^l representation) for the υ = 1 state of the ($^{13}C_\alpha$)BZF-FA isotopologue of the isomer ***I*** of BZF-FA.

Parameter	Experiment
	υ = 1
A_0 [MHz]	1179.780(5)
B_0 [MHz]	1093.874(8)
C_0 [MHz]	787.1308(6)
D_J [kHz]	3.890(4)
D_{JK} [kHz]	[-4.822(6)][b]
D_K [kHz]	[1.828(4)]
d_1 [kHz]	[-0.448(2)]
d_2 [kHz]	[0.9639(8)]
N[c]	10
σ[d] [kHz]	2.9

[a] Standard errors in units of the last digit. [b] Values in brackets are fixed to the corresponding υ = 1 state values of the parent species. [c] Number of the fitted lines; [d] Root-mean square error of the fit.

Table S2.2.5b. Numbered (*N*) experimental rotational transitions for the υ = 1 state of the ($^{13}C_\alpha$)BZF-FA isotopologue of the isomer ***I*** of BZF-FA. Table reports: the experimental frequencies (ν_{EXP}) and the residuals (observed - calculated differences; $\Delta\nu$).

N	J	K_a	K_c	J'	K'_a	K'_c	ν_{EXP} [MHz]	$\Delta\nu$ [MHz]
1	4	1	4	3	1	3	6643.2054	-0.0019
2	4	0	4	3	0	3	6644.8304	-0.0060
3	4	1	3	3	1	2	7372.6209	0.0001
4	5	1	5	4	1	4	8217.8400	0.0044
5	5	0	5	4	0	4	8217.9938	0.0026
6	5	1	4	4	1	3	8919.1053	0.0005
7	7	1	7	6	1	6	11365.4910	0.0011
8	7	0	7	6	0	6	11365.4910	0.0001
9	8	1	8	7	1	7	12939.0723	-0.0012
10	8	0	8	7	0	7	12939.0723	-0.0013

S2.2.6. The ($^{13}C_β$)BZF-FA isotopologue.

Table S2.2.6a. Experimentally determined spectroscopic parameters (Watson's *S*-reduction, III^l representation) for the υ = 1 state of the ($^{13}C_β$)BZF-FA isotopologue of the isomer ***I*** of BZF-FA.

Parameter	**Experiment**
	υ = 1
A_0 [MHz]	1178.786(7)
B_0 [MHz]	1094.24(1)
C_0 [MHz]	788.0239(9)
D_J [kHz]	3.915(9)
D_{JK} [kHz]	[-4.822(6)][b]
D_K [kHz]	[1.828(4)]
d_1 [kHz]	[-0.448(2)]
d_2 [kHz]	[0.9639(8)]
N[c]	10
σ[d] [kHz]	4.1

[a] Standard errors in units of the last digit. [b] Values in brackets are fixed to the corresponding υ = 1 state values of the parent species. [c] Number of the fitted lines; [d] Root-mean square error of the fit.

Table S2.2.6b. Numbered (*N*) experimental rotational transitions for the υ = 1 state of the ($^{13}C_β$)BZF-FA isotopologue of the isomer ***I*** of BZF-FA. Table reports the experimental frequencies (ν_{EXP}) and the residuals (observed - calculated differences; $\Delta\nu$).

N	J	K_a	K_c	J'	K'_a	K'_c	ν_{EXP} [MHz]	$\Delta\nu$ [MHz]
1	4	1	4	3	1	3	6649.2459	0.0044
2	4	0	4	3	0	3	6650.8083	0.0017
3	4	1	3	3	1	2	7375.5507	0.0005
4	5	1	5	4	1	4	8225.6308	0.0055
5	5	0	5	4	0	4	8225.7658	-0.0070
6	5	1	4	4	1	3	8924.3651	-0.0011
7	6	1	6	5	1	5	9801.3129	-0.0039
8	6	0	6	5	0	5	9801.3258	-0.0034
9	7	1	7	6	1	6	11376.8330	0.0048
10	7	0	7	6	0	6	11376.8330	0.0038

S2.2.7. The ($^{13}C_4$)BZF-FA isotopologue.

Table S2.2.7a. Experimentally determined spectroscopic parameters (Watson's *S*-reduction, *III*[l] representation) for the υ = 1 state of the ($^{13}C_4$)BZF-FA isotopologue of the isomer ***I*** of BZF-FA.

Parameter	**Experiment**
	υ = 1
A_0 [MHz]	1172.73(1)
B_0 [MHz]	1090.81(1)
C_0 [MHz]	786.9611(4)
D_J [kHz]	3.890 (3)
D_{JK} [kHz]	[-4.822(6)][b]
D_K [kHz]	[1.828(4)]
d_1 [kHz]	[-0.448(2)]
d_2 [kHz]	[0.9639(8)]
N[c]	12
σ[d] [kHz]	2.7

[a] Standard errors in units of the last digit. [b] Values in brackets are fixed to the corresponding υ = 1 state values of the parent species. [c] Number of the fitted lines; [d] Root-mean square error of the fit.

Table S2.2.7b. Numbered (*N*) experimental rotational transitions for the υ = 1 state of the ($^{13}C_4$)BZF-FA isotopologue of the isomer ***I*** of BZF-FA. Table reports the experimental frequencies (ν_{EXP}) and the residuals (observed - calculated differences; $\Delta\nu$).

N	J	K_a	K_c	J'	K'_a	K'_c	ν_{EXP} [MHz]	$\Delta\nu$ [MHz]
1	4	1	4	3	1	3	6637.2371	0.0024
2	4	0	4	3	0	3	6638.6916	-0.0002
3	5	1	5	4	1	4	8211.4568	-0.0005
4	5	0	5	4	0	4	8211.5868	-0.0049
5	5	1	4	4	1	3	8902.6359	0.0005
6	6	1	6	5	1	5	9785.0257	-0.0029
7	6	0	6	5	0	5	9785.0452	0.0056
8	6	1	5	5	1	4	10470.8335	-0.0005
9	7	1	7	6	1	6	11358.4296	0.0020
10	7	0	7	6	0	6	11358.4296	0.0012
11	8	1	8	7	1	7	12931.6715	-0.0011
12	8	0	8	7	0	7	12931.6715	-0.0011

S2.2.8. The ($^{13}C_5$)BZF-FA isotopologue.

Table S2.2.8a. Experimentally determined spectroscopic parameters (Watson's *S*-reduction, *III*[l] representation) for the υ = 1 state of the ($^{13}C_5$)BZF-FA isotopologue of the isomer ***I*** of BZF-FA.

Parameter	**Experiment**
	υ = 1
A_0 [MHz]	1168.897(3)
B_0 [MHz]	1092.213(5)
C_0 [MHz]	782.1475(4)
D_J [kHz]	3.909(4)
D_{JK} [kHz]	[-4.822(6)][b]
D_K [kHz]	[1.828(4)]
d_1 [kHz]	[-0.448(2)]
d_2 [kHz]	[0.9639(8)]
N[c]	10
σ[d] [kHz]	1.9

[a] Standard errors in units of the last digit. [b] Values in brackets are fixed to the corresponding υ = 1 state values of the parent species. [c] Number of the fitted lines; [d] Root-mean square error of the fit.

Table S2.2.8b. Numbered (*N*) experimental rotational transitions for the υ = 1 state of the ($^{13}C_5$)BZF-FA isotopologue of the isomer ***I*** of BZF-FA. Table reports the experimental frequencies (ν_{EXP}) and the residuals (observed - calculated differences; $\Delta\nu$).

N	J	K_a	K_c	J'	K'_a	K'_c	ν_{EXP} [MHz]	$\Delta\nu$ [MHz]
1	4	1	4	3	1	3	6602.7633	0.0036
2	4	0	4	3	0	3	6603.9389	0.0015
3	4	1	3	3	1	2	7324.6011	0.0002
4	5	1	5	4	1	4	8167.2402	-0.0025
5	5	0	5	4	0	4	8167.3408	-0.0020
6	5	1	4	4	1	3	8865.0997	-0.0006
7	6	1	6	5	1	5	9731.1732	0.0026
8	6	0	6	5	0	5	9731.1732	-0.0050
9	7	1	7	6	1	6	11294.9365	0.0019
10	7	0	7	6	0	6	11294.9365	0.0013

S2.2.9. The ($^{13}C_6$)BZF-FA isotopologue.

Table S2.2.9a. Experimentally determined spectroscopic parameters (Watson's *S*-reduction, III^l representation) for the υ = 1 state of the ($^{13}C_6$)BZF-FA isotopologue of the isomer ***I*** of BZF-FA.

Parameter	Experiment
	υ = 1
A_0 [MHz]	1171.869(8)
B_0 [MHz]	1089.31(1)
C_0 [MHz]	781.581(1)
D_J [kHz]	3.900(1)
D_{JK} [kHz]	[-4.822(6)][b]
D_K [kHz]	[1.828(4)]
d_1 [kHz]	[-0.448(2)]
d_2 [kHz]	[0.9639(8)]
N[c]	10
σ[d] [kHz]	5.0

[a] Standard errors in units of the last digit. [b] Values in brackets are fixed to the corresponding υ = 1 state values of the parent species. [c] Number of the fitted lines; [d] Root-mean square error of the fit.

Table S2.2.9b. Numbered (*N*) experimental rotational transitions for the υ = 1 state of the ($^{13}C_6$)BZF-FA isotopologue of the isomer ***I*** of BZF-FA. Table reports the experimental frequencies (ν_{EXP}) and the residuals (observed - calculated differences; $\Delta\nu$).

N	J	K_a	K_c	J'	K'_a	K'_c	ν_{EXP} [MHz]	$\Delta\nu$ [MHz]
1	4	1	4	3	1	3	6598.3775	-0.0089
2	4	0	4	3	0	3	6599.8381	-0.0048
3	4	1	3	3	1	2	7324.6091	-0.0006
4	5	1	5	4	1	4	8161.8552	0.0085
5	5	0	5	4	0	4	8161.9857	0.0053
6	5	1	4	4	1	3	8861.4152	0.0014
7	6	1	6	5	1	5	9724.6530	-0.0014
8	6	0	6	5	0	5	9724.6675	0.0022
9	7	1	7	6	1	6	11287.2858	-0.0030
10	7	0	7	6	0	6	11287.2858	-0.0038

S2.2.10. The ($^{13}C_7$)BZF-FA isotopologue.

Table S2.2.10a. Experimentally determined spectroscopic parameters (Watson's *S*-reduction, III^l representation) for the υ = 1 state of the ($^{13}C_7$)BZF-FA isotopologue of the isomer ***I*** of BZF-FA.

Parameter	**Experiment**
	υ = 1
A_0 [MHz]	1175.922(4)
B_0 [MHz]	1088.025(7)
C_0 [MHz]	785.4454 (5)
D_J [kHz]	3.884(5)
D_{JK} [kHz]	[-4.822(6)][b]
D_K [kHz]	[1.828(4)]
d_1 [kHz]	[-0.448(2)]
d_2 [kHz]	[0.9639(8)]
N[c]	10
σ[d] [kHz]	2.4

[a] Standard errors in units of the last digit. [b] Values in brackets are fixed to the corresponding υ = 1 state values of the parent species. [c] Number of the fitted lines; [d] Root-mean square error of the fit.

Table S2.2.10b. Numbered (*N*) experimental rotational transitions for the υ = 1 state of the ($^{13}C_7$)BZF-FA isotopologue of the isomer ***I*** of BZF-FA. Table reports: the experimental frequencies (ν_{EXP}) and the residuals (observed - calculated differences; $\Delta\nu$).

N	J	K_a	K_c	J'	K'_a	K'_c	ν_{EXP} [MHz]	$\Delta\nu$ [MHz]
1	4	1	4	3	1	3	6626.3452	-0.0041
2	4	0	4	3	0	3	6628.1198	-0.0011
3	4	1	3	3	1	2	7350.6855	-0.0004
4	5	1	5	4	1	4	8197.6637	0.0007
5	5	0	5	4	0	4	8197.8435	0.0055
6	5	1	4	4	1	3	8892.8782	0.0007
7	6	1	6	5	1	5	9768.2152	-0.0015
8	6	0	6	5	0	5	9768.2325	0.0004
9	7	1	7	6	1	6	11338.5857	-0.0002
10	7	0	7	6	0	6	11338.5857	-0.0014

S2.2.11. The BZF-(^{13}C)FA isotopologue.

Table S2.2.11a. Experimentally determined spectroscopic parameters (Watson's *S*-reduction, *III*^{l} representation) for the υ = 1 state of the BZF-(^{13}C)FA isotopologue of the isomer ***I*** of BZF-FA.

Parameter	**Experiment**
	υ = 1
A_0 [MHz]	1178.526(4)
B_0 [MHz]	1082.367(8)
C_0 [MHz]	781.4010(6)
D_J [kHz]	3.886(6)
D_{JK} [kHz]	[-4.822(6)][b]
D_K [kHz]	[1.828(4)]
d_1 [kHz]	[-0.448(2)]
d_2 [kHz]	[0.9639(8)]
N[c]	10
σ[d] [kHz]	2.6

[a] Standard errors in units of the last digit. [b] Values in brackets are fixed to the corresponding υ = 1 state values of the parent species. [c] Number of the fitted lines; [d] Root-mean square error of the fit.

Table S2.2.11b. Numbered (*N*) experimental rotational transitions for the υ = 1 state of the BZF-(^{13}C)FA isotopologue of the isomer ***I*** of BZF-FA. Table reports the experimental frequencies(ν_{EXP}) and the residuals (observed - calculated differences; $\Delta\nu$).

N	J	K_a	K_c	J'	K_a'	K_c'	ν_{EXP} [MHz]	$\Delta\nu$ [MHz]
1	4	1	4	3	1	3	6595.7776	-0.0023
2	4	0	4	3	0	3	6598.0496	0.0029
3	4	1	3	3	1	2	7329.5968	-0.0004
4	5	1	5	4	1	4	8159.1956	0.0020
5	5	0	5	4	0	4	8159.4402	0.0023
6	5	1	4	4	1	3	8860.4043	0.0002
7	6	1	6	5	1	5	9721.6752	-0.0035
8	6	0	6	5	0	5	9721.6983	-0.0039
9	7	1	7	6	1	6	11283.9607	0.0040
10	7	0	7	6	0	6	11283.9607	0.0019

S3. Semi-experimental equilibrium structures

S3.1. Theoretical background.

The quest of accurate structures is a topic of fundamental importance in the context of non-covalent interactions due to their great impact in nature. The semi-experimental equilibrium structure (r_e^{SE}) approach offers a way to determine equilibrium structural parameters with good accuracy.[26,27] This method requires the experimental determination of the vibrational ground-state rotational constants $B^i_{\text{exp},0}$ for a (possibly) large set of isotopologues to be combined with vibrational contributions quantum chemically calculated (ΔB^i_{vib}).
To evaluate all or selected geometrical parameters, a least-square fitting procedure is performed on the semi-experimental equilibrium rotational constants $B^i_{\text{SE},e}$, which are obtained by correcting the ground-state rotational cosnntants ($B^i_{\text{exp},0}$) for the corresponding computed vibrational corrections ($\Delta B^i_{\text{calc},vib}$). As evident from equation (3), within second-order vibrational perturbation theory (VPT2),[28] the evaluation of vibrational corrections requires the calculation of the vibration-rotation interaction constants (α^i_r), which in turn need anharmonic force field computations (carried out at the B3 level for isolated BZF and CP-B3 for the isomer ***I*** of BZF-FA):

$$B^i_{\text{SE},e} = B^i_{\text{exp},0} - \Delta B^i_{\text{calc},vib} = B^i_{\text{exp},0} + \frac{1}{2}\sum_r \alpha^i_r \quad (3)$$

In the equation above, the sum runs over the *r* vibrational normal modes and *i* refers to the inertial axes (*a*, *b*, *c*).
In the present investigation, two semi-experimental structures have been determined: that of benzofuran (BZF) and that of the isomer ***I*** of the BZF-FA adduct. These are detailed in sections S3.2 and S3.3, respectively.
Despite the large number of isotopic substitutions, a full r_e^{SE} structure cannot be obtained for the BZF-FA adduct. However, using the so-called "template model" (TM) approach,[27] the intramolecular parameters of both the molecular fragments (i.e. BZF and FA) can be accurately determined and kept fixed in the least-square fitting procedure for the molecular adduct. According to the TM approach, the intramolecular parameters of BZF and FA within the complex are determined as:

$$r_e^{SE}(intramolecular, adduct) = r_e^{QM}(adduct) + \Delta TM \quad (4)$$

where

$$\Delta TM = r_e^{SE}(monomer) - r_e^{QM}(monomer) \quad (5)$$

$r_e^{QM}(adduct)$ is a generic intramolecular parameter of the molecular complex optimized at a given level of theory (jB2 in the present case) and ΔTM is the difference, for the isolated monomer, between the corresponding semi-experimental equilibrium parameter and its quantum-chemical value (at the same level of theory

as above, i.e., jB2 here). The application of the TM model as described above implies that the r_e^{SE} structures of both fragments are known. While the r_e^{SE} of FA is available in the literature (taken from the SMART lab database [27]), that of BZF has been purposely evaluated (see section S3.2).
Note that, for a given parameter, whenever the r_e^{SE} value of the monomer is not available, then the r_e^{QM} counterpart (at the jB2 level in this study) is used.

S3.2. Benzofuran.

An accurate r_e^{SE} structure of isolated benzofuran has been determined based on the availability in the literature of the rotational constants of 10 isotopologues (parent, all monosubstituted ^{13}C and ^{18}O species).[29]

Table S3.2.1. The r_e^{SE} structure of benzofuran.

C						
C	1	R1				
C	2	R2	1	A1		
C	1	R3	2	A2	3	D0
H	1	R4	2	A3	3	D180
H	2	R5	1	A4	5	D0
H	3	R6	2	A5	6	D0
H	4	R7	1	A6	5	D0
C	3	R8	2	A7	1	D0
C	4	R9	1	A8	2	D0
C	9	R10	3	A9	2	D180
O	10	R11	4	A10	1	D180
H	11	R12	9	A11	3	D0
C	11	R13	9	A12	3	D180
H	14	R14	12	A13	10	D180
**FITTED PARAMETERS**				**PARAMETERS FIXED at the B2 LEVEL**		
R1		1.4036(4)		R4		1.08023
R2		1.3861(3)		R5		1.08036
A1		121.29(1)		R6		1.08057
R3		1.3875(3)		R7		1.07940
A2		121.41(1)		R12		1.07528
R8		1.3995(6)		R14		1.07428
A7		118.29(2)		A3		119.35051
R9		1.3848(6)		A4		119.09253
A8		116.25(2)		A5		120.74438
R10		1.4399(5)		A6		122.14555
A9		135.61(2)		A11		127.88372
R11		1.3651(4)		A13		115.20154
A10		125.72(3)		D0		0.0
R13		1.3515(4)		D180		180.0
A12		105.77(2)				

According to equation (3), the semi-experimental equilibrium rotational constants of each isotopologue have been derived by subtracting from the experimental ground-state rotational constants the corresponding B3 vibrational corrections. In the least-square fit procedure, the parameters involving the hydrogen atoms have been kept fixed at their corresponding jB2 values. Benzofuran is a planar molecule (the inertial defect, Δ_c, should thus be equal to zero), with all considered parent and isotopic substituted species having a Δ_c value of about -0.06 uÅ^2 in the vibrational ground state. It is worth to mention that, when a structural determination is constrained to planarity, only two rotational constants of each isotopologue are linearly independent and can be used in the fitting procedure. The determined structure of isolated benzofuran is reported in Table S3.2.1, where the 15 adjusted parameters (σ^2=2·10^{-6}) are reported. Table S3.2.2 collects the experimental rotational constants used in the fit together with the corresponding vibrational corrections

Table S3.2.2. Ground-state rotational constants[a] and corresponding B3 vibrational corrections for the different isotopologues of benzofuran. Values in MHz. The isotopologues numbering follows that of Table S3.2.1.

Isotopologue	A_0	B_0	C_0	ΔA_{vib}	ΔB_{vib}	ΔC_{vib}
PARENT	3916.565	1660.795	1166.418	-29.202	-10.321	-7.571
$^{13}C_1$	3900.582	1636.727	1153.101	-28.940	-10.159	-7.464
$^{13}C_2$	3903.641	1635.481	1152.750	-29.000	-10.139	-7.459
$^{13}C_3$	3855.715	1655.198	1158.221	-28.630	-10.238	-7.490
$^{13}C_4$	3857.094	1656.390	1158.930	-28.650	-10.241	-7.492
$^{13}C_9$	3898.733	1660.587	1164.730	-28.956	-10.257	-7.518
$^{13}C_{10}$	3904.842	1660.539	1165.251	-28.910	-10.263	-7.518
$^{13}C_{11}$	3875.783	1646.803	1155.897	-28.810	-10.186	-7.473
$^{18}O_{12}$	3848.624	1635.757	1148.039	-28.625	-10.081	-7.407
$^{13}C_{14}$	3916.629	1631.908	1152.101	-29.120	-10.129	-7.460

[a] From ref. [29] and rounded to the third decimal.

S3.3. The isomer *I* of the Benzofuran-Formaldehyde complex.

The isolated benzofuran and formaldehyde have been used as template molecules. Their r_e^{SE} structures (BZF: section S3.2; FA: CCse structure[27] available at smart.sns.it/molecules/) thus provide the intramolecular geometrical parameter corrections (ΔTM) to be applied to the corresponding CP-jB2 calculated values of the isomer ***I*** of the BZF-FA adduct:

$$r_e^{SE}(intramolecular) = r_e^{CP-jB2} + \Delta TM \quad (6)$$

where

$$\Delta TM = r_e^{SE}(BZF/FA) - r_e^{jB2}(BZF/FA) \quad (7)$$

On top of this CP-jB2 intramolecular-corrected structure, three intermolecular parameters (the $C_{FA}\cdots C_{\beta}$ distance: 3.2257±0.0006 Å; the $C_{FA}\cdots C_{\beta}$-C_4 angle: 90.18±0.04°; the dihedral $C_{FA}\cdots C_{\beta}$-C_4-C_3 angle: -98.31±0.03°) have been adjusted by means of a least-square fit (σ^2 = 0.2) to reproduce the eleven sets of semi-experimental equilibrium rotational constants.
The data employed (rotational constants and vibrational corrections) in the fit are collected in Table S3.3.1. While Figure SF3.3.1 provides a graphical representation of the r_e^{SE} structure of the BZF-FZ complex (isomer ***I***), a list of selected intermolecular parameters is reported in Table S3.3.2, with the determined semi-experimental structure in Cartesian coordiantes being reported in Table S3.3.3.

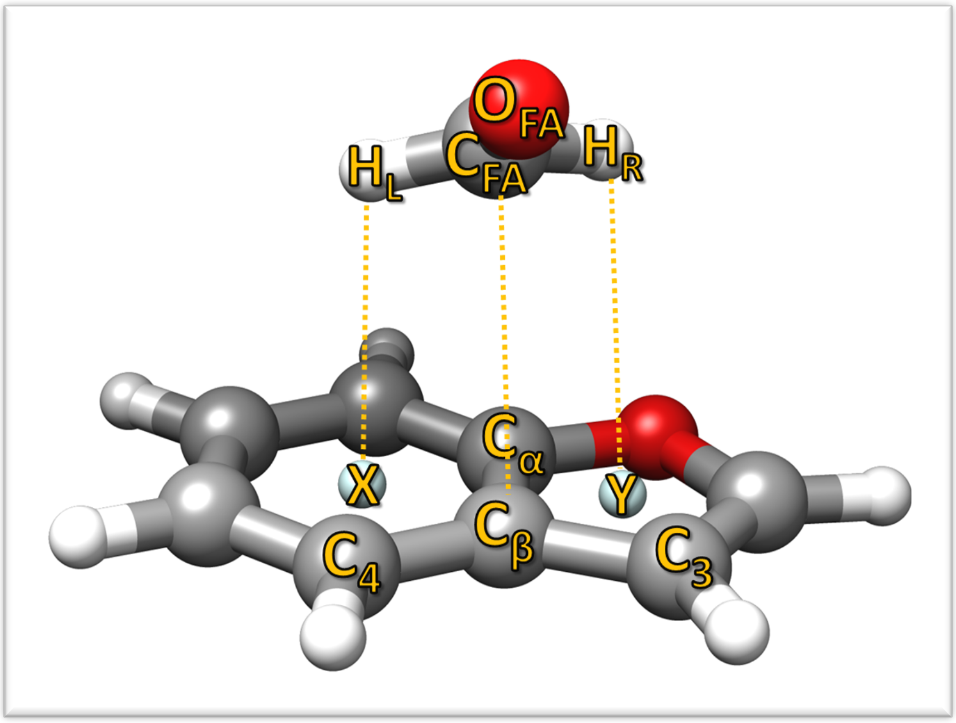

Figure SF3.3.1. Significant intermolecular contacts in the isomer ***I*** of the BZF-FA adduct. The X and Y dummy atoms are the centers of mass of benzene and furan rings, respectively.

Table S3.3.1. Ground-state rotational constants and the corresponding CP-B3 vibrational corrections. Values in MHz.

Isotopologue	A_0	B_0	C_0	A_0-A_e	B_0-B_e	C_0-C_e
PARENT	1180.904	1096.203	788.281	-9.224	-20.572	-9.628
^{13}C(FA)	1178.526	1082.367	781.401	-8.736	-16.717	-8.269
^{18}O(FA)	1179.612	1047.274	762.004	-10.032	-20.691	-10.023
$^{13}C_2$	1165.146	1095.958	781.175	-10.074	-18.838	-8.669
$^{13}C_3$	1169.799	1092.838	785.112	-9.194	-21.380	-9.969
$^{13}C_4$	1172.729	1090.807	786.961	-9.292	-21.356	-10.185
$^{13}C_5$	1168.897	1092.213	782.147	-10.363	-21.303	-10.371
$^{13}C_6$	1171.869	1089.308	781.581	-10.697	-20.589	-10.086
$^{13}C_7$	1175.922	1088.025	785.445	-9.909	-21.479	-10.075
$^{13}C_\alpha$	1179.780	1093.874	787.131	-8.366	-18.725	-9.128
$^{13}C_\beta$	1178.786	1094.244	788.024	-7.914	-18.958	-9.166

Table S3.3.2. Significant r_e^{SE} intermolecular parameters of the Isomer ***I*** of the BZF-FA adduct.

**Intermolecular parameter**	r_e^{SE}
$C_{FA}\cdots C_\beta$ [Å]	3.2257
$H_L\cdots X$ [Å]	2.7861
$H_R\cdots Y$ [Å]	2.9619
O_{FA}-$C_{FA}\cdots C_\beta$ [°]	114.99
$C_{FA}\cdots C_\beta$-C_α [°]	81.77
$C_{FA}\cdots C_\beta$-C_4 [°]	90.18
$C_{FA}\cdots C_\beta$-C_4-C_3 [°]	-98.31

Table S3.3.3. The r_e^{SE} structure of the isomer ***I*** of the BZF-FA adduct in Cartesian coordinates (Å).

C	0.00000000	0.00000000	0.00000000
C	0.00000000	0.00000000	1.40399000
C	1.18425116	0.00000000	2.12409082
C	1.18441514	-0.00091494	-0.72380817
H	-0.94156131	0.00010386	-0.52942830
H	-0.94388522	0.00343746	1.92925271
H	1.17460835	0.00935617	3.20424726
H	1.19787142	-0.00108682	-1.80318428
C	2.39134925	-0.00083046	1.41502558
C	2.35457410	-0.00250545	0.01727197
C	3.78809016	0.01723913	1.76387679
O	3.62074807	0.01354914	-0.49192283
H	4.22173796	0.03047788	2.74757254
C	4.45951980	0.02477999	0.59128354
H	5.50787255	0.03848583	0.35732041
C	2.13568032	3.18179590	0.95604476
O	2.30409688	3.85424805	1.94319161
H	2.96813270	2.93287765	0.28051602
H	1.14727709	2.78504551	0.68539483

S4. References

[1] P. Pracht, F. Bohle, S. Grimme, *Phys. Chem. Chem. Phys.* **2020**, *22*, 7169–7192.

[2] a) A. D. Becke, *J. Chem. Phys*. **1993**, *98*, 5648-5652; b) P.J. Stephens, F.J. Devlin, C.F. Chabalowski, M.J. Frisch, *J. Phys. Chem.* **1994**, *98*, 11623-11627; c) V. Barone, P. Cimino, E. Stendardo, *J. Chem. Theory Comput.* **2008**, *4*, 751–764; d) SNSD basis set available at: https://smart.sns.it/; e) S. Grimme, J. Antony, S. Ehrlich, H. Krieg *J. Chem. Phys*. **2010**, *132*, 154104; f) S. Grimme, S. Ehrlich, L. Goerigk, *J. Comp. Chem*. **2011**, *32*, 1456-1465;

[3] a) S. Grimme, *J. Chem. Phys.* **2006**, *124*, 034108; b) M. Biczysko, G. Scalmani, J. Bloino, V. Barone, *J. Chem. Theory Comput.* 2010, **6**, 2115-2125; c) E. Papajak, D. G. Truhlar, *J. Chem. Theory Comput.* **2011**, *7*, 10−18; d) E. Papajak, J. Zheng, X. Xu, H. R. Leverentz, D. G. Truhlar, *J. Chem. Theory Comput.* **2011**, 7, 3027–3034.

[4] S. F. Boys, F. Bernardi, *Mol. Phys*. **1970**, *19*, 553-566.

[5] S. Alessandrini, V. Barone, C. Puzzarini, *J. Chem. Theory Comput*. **2020**, *16*, 988−1006.

[6] K. Raghavachari, G. W. Trucks, J. A. Pople, M. Head-Gordon, *Chem. Phys. Lett.* **1989**, *157*, 479–483.

[7] C. Møller, M. S. Plesset, *Phys. Rev.* **1934**, *46*, 618–622.

[8] T. Helgaker, W. Klopper, H. Koch, J. Noga, *J. Chem. Phys.* **1997**, *106*, 9639–9646.

[9] K. A. Peterson, T. H. Dunning, *J. Chem. Phys.* **2002**, *117*, 10548–10560.

[10] Gaussian 16, Revision C.01, M. J. Frisch, G. W. Trucks, H. B. Schlegel, G. E. Scuseria, M. A. Robb, J. R. Cheeseman, G. Scalmani, V. Barone, G. A. Petersson, H. Nakatsuji, X. Li, M. Caricato, A. V. Marenich, J. Bloino, B. G. Janesko, R. Gomperts, B. Mennucci, H. P. Hratchian, J. V. Ortiz, A. F. Izmaylov, J. L. Sonnenberg, D. Williams-Young, F. Ding, F. Lipparini, F. Egidi, J. Goings, B. Peng, A. Petrone, T. Henderson, D. Ranasinghe, V. G. Zakrzewski, J. Gao, N. Rega, G. Zheng, W. Liang, M. Hada, M. Ehara, K. Toyota, R. Fukuda, J. Hasegawa, M. Ishida, T. Nakajima, Y. Honda, O. Kitao, H. Nakai, T. Vreven, K. Throssell, J. A. Montgomery, Jr., J. E. Peralta, F. Ogliaro, M. J. Bearpark, J. J. Heyd, E. N. Brothers, K. N. Kudin, V. N. Staroverov, T. A. Keith, R. Kobayashi, J. Normand, K. Raghavachari, A. P. Rendell, J. C. Burant, S. S. Iyengar, J. Tomasi, M. Cossi, J. M. Millam, M. Klene, C. Adamo, R. Cammi, J. W. Ochterski, R. L. Martin, K. Morokuma, O. Farkas, J. B. Foresman, and D. J. Fox, Gaussian, Inc., Wallingford CT, **2016**.

[11] E. D. Glendening, J, K. Badenhoop, A. E. Reed, J. E. Carpenter, J. A. Bohmann, C. M. Morales, P. Karafiloglou, C. R. Landis, F. Weinhold, Theoretical Chemistry Institute, University of Wisconsin, Madison, **2018**; b) E. D. Glendening, C. R. Landis, and F. Weinhold, *WIREs Comput. Mol. Sci*. **2012**, *2*, 1-42.

[12] a) A. D. Becke, *J. Chem. Phys*. **1993**, *98*, 5648-5652; b) P.J. Stephens, F.J. Devlin, C.F. Chabalowski, M.J. Frisch, *J. Phys. Chem.* **1994**, *98*, 11623-11627.

[13] a) T. Fornaro, M. Biczysko, J. Bloino, V. Barone, *Phys. Chem. Chem. Phys.* **2016**, *18*, 8479–8490; b) E. Papajak, H. R. Leverentz, J. Zheng, D. G. Truhlar, *J. Chem. Theory Comput.* **2009**, *5*, 1197–1202.

[14] a) B. Jeziorski, R. Moszynski, K. Szalewicz, *Chem. Rev*. **1994**, *94*, 1887 – 1930; b) T. M. Parker, L. A. Burns, R. M. Parrish, A. G. Ryno, C. D. Sherrill, *J. Chem. Phys*. **2014**, *140*, 094106.

[15] R. M. Parrish, L. A. Burns, D. G. A. Smith, A. C. Simmonett, A. E. DePrince III, E. G. Hohenstein, U. Bozkaya, A. Y. Sokolov, R. Di Remigio, R. M. Richard, J. F. Gonthier, A. M. James, H. R. McAlexander, A. Kumar, M. Saitow, X. Wang, B. P. Pritchard, P. Verma, H. F. Schaefer III, K. Patkowski, R. A. King, E. F. Valeev, F. A. Evangelista, J. M. Turney, T. Daniel Crawford, C. D. Sherril, *J. Chem. Theory Comput*. **2017**, *13*, 3185 – 3197.

[16] Y. Shi, J. Zhang, M. Shi, S. P. O'Connor, S. N. Bisaha, C. Li, D. Sitkoff, A. T. Pudzianowski, S. Chong, H. E. Klei, K. Kish, J. Yanchunas, E. C.-K. Liu, K. S. Hartl, S. M. Seiler, T. E. Steinbacher, W. A. Schumacher, K. S. Atwal, P. D. Stein, *Bioorganic Med. Chem. Lett.* **2009**, *19*, 4034-4041.

[17] H.M. Berman, J. Westbrook, Z. Feng, G. Gilliland, T.N. Bhat, H. Weissig, I.N. Shindyalov, P.E. Bourne, *Nucleic Acids Res*. **2000**, *28*, 235-242.

[18] E.F. Pettersen, T.D. Goddard, C.C. Huang, G.S. Couch, D.M. Greenblatt, E.C. Meng, T.E. Ferrin, *J. Comput. Chem.* **2004**, 25,1605-1612.[19] J.-U. Grabow, W. Stahl, H. Dreizler, *Rev. Sci. Instrum.* **1996**, *67*, 4072–4084.
[20] J.-U. Grabow, Q. Gou, G. Feng, 72nd International Symposium on Molecular Spectroscopy, **2017**, TH03.
[21] FTMW++program: J.-U. Grabow, Habilitationsschrift, Universität Hannover, Hannover, **2004**. Program available at: https://seafile.projekt.uni-hannover.de/d/b05d0b38d02c4bc79969/
[22] H. M. Pickett, *J. Mol. Spectrosc.* **1991**, *148*, 371–377.
[23] J. K. G. Watson, in Vibrational Spectra and Structure, edited by J. R. Durig (Elsevier, New York, Amsterdam) **1977**, Vol. 6, pp. 1–89.
[24] W. Li, A. Maris, C. Calabrese, I. Usabiaga, W. D. Geppert, L. Evangelisti, S. Melandri, *Phys. Chem. Chem. Phys.* **2019**, *21*, 23559–23566.
[25] R. S. Ruoff, T. D. Klots, T. Emilsson, H. S. Gutowsky, *J. Chem. Phys.* **1990**, *93*, 3142–3150.
[26] a) P. Pulay, W. Meyer, J. E. Boggs, *J. Chem. Phys.* **1978**, *68*, 5077–5085; b) F. Pawłowski, P. Jørgensen, J. Olsen, F. Hegelund, T. Helgaker, J. Gauss, K. L. Bak, J. F. Stanton, *J. Chem. Phys.* **2002**, *116*, 6482–6496.
[27] M. Piccardo, E. Penocchio, C. Puzzarini, M. Biczysko, V. Barone, *J. Phys. Chem. A* **2015**, *119*, 2058−2082.
[28] a) I. M. Mills, Vibration-Rotation Structure in Asymmetric- and Symmetric-Top Molecules. In *Molecular Spectroscopy: Modern Research*; Rao, K.N., Mathews, C.W., Eds.; Academic Press: New York, NY, USA, **1972**. b) V. Barone, *J. Chem. Phys.* **2005**, *122*, 014108.
[29] A. Maris, B. M. Giuliano, S. Melandri, P. Ottaviani, W. Caminati, L. B. Favero, B. Velino, *Phys. Chem. Chem. Phys.* **2005**,*7*, 3317-3322.

Unterstützende Informationen

S1. Rechnerische Details:

S1.1. Konformationssuche und energetische Charakterisierung der binären Benzofuran-Formaldehyd-Addukte.
S1.2. Rotationsparameter.
S1.3. Natural Bond Orbital (NBO) Analyse.
S1.4. Natural Energy Decomposition Analysis (NEDA).
S1.5. Symmetry Adapted Perturbation Theory (SAPT) Analyse.
S1.6. Analyse der nicht-kovalenten Wechselwirkungen im PDB:3HPT YET 2.D Ligandenmodell.

S2. Experimentelle Details:

S2.1. Beschreibung des Versuchsaufbaus und des Zuordnungsverfahrens.
S2.2. Experimentelle Rotationsübergänge und Anpassungs-Ergebnisse von Isomer ***I*** des Benzofuran-Formaldehyd-Komplexes:
S2.2.1. Die Hauptspezies.
S2.2.2. Das BZF-(^{18}O)FA-Isotopologe.
S2.2.3. Das ($^{13}C_2$)BZF-FA-Isotopologe.
S2.2.4. Das ($^{13}C_3$)BZF-FA-Isotopologe.
S2.2.5. Das ($^{13}C_\alpha$)BZF-FA-Isotopologe.
S2.2.6. Das ($^{13}C_\beta$)BZF-FA-Isotopologe.
S2.2.7. Das ($^{13}C_4$)BZF-FA-Isotopologe.
S2.2.8. Das ($^{13}C_5$)BZF-FA-Isotopologe.
S2.2.9. Das ($^{13}C_6$)BZF-FA-Isotopologe.
S2.2.10. Das ($^{13}C_7$)BZF-FA-Isotopologe.
S2.2.11. Das BZF-(^{13}C)FA-Isotopologe.

S3. Semi-experimentelle Gleichgewichtsstrukturen:

S3.1. Theoretische Grundlagen.
S3.2. Benzofuran.
S3.3. Das Isomer ***I*** des Benzofuran-Formaldehyd-Komplexes.

S4. Referenzen

S1. Rechnerische Details

S1.1. Konformationssuche und energetische Charakterisierung der binären Benzofuran-Formaldehyd-Addukte.

Ausgehend von der Auswahl eines Dutzends von Ausgangsstrukturen, gefolgt von einer systematischen Suche unter Verwendung des CREST-Kodes,[1] wurde eine große Anzahl von stationären Punkten mit niedriger Energie der Potenzialoberfläche (PES) auf dem B3LYP-D3(BJ)/SNSD-Niveau[2] (im Folgenden B3) gefunden, wobei D3(BJ) für das D3-Schema zur Behandlung von Dispersionseffekten in Kombination mit der Becke-Johnson (BJ)-Dämpfungsfunktion steht. Anschließend wurden echte Energieminima durch Diagonalisierung der analytischen Hessians (alle Eigenwerte sind positiv) identifiziert und auf einem höheren Theorieniveau erneut optimiert, wobei das B2PLYP-Doppelhybridfunktional in Kombination mit der D3(BJ)-Korrektur und in Verbindung mit dem jun-cc-pVTZ-Basissatz (im Folgenden als jB2 bezeichnet) verwendet wurde.[2f,3] Sowohl bei den B3- als auch bei den jB2-Berechnungen wurde die counterpoise (CP) Korrektur[4] in jedem Optimierungsschritt einbezogen, was Theorieniveaus entspricht, die als CP-B3 bzw. CP-jB2 bezeichnet werden. Dieses Verfahren führte auf dem CP-jB2-Niveau zur Charakterisierung von vierzehn tiefliegenden Energieminima (innerhalb von 4 kJ mol $^{-1}$über dem globalen Minimum), deren kartesische Koordinaten in Tabelle S1.1.1 angegeben sind.

Um eine genauere Bewertung der relativen Energien aller Minima und der wichtigsten Übergangszustände zu erhalten und die Wechselwirkungsenergien genau abzuschätzen, wurde das so genannte "jun-Cheap"-Verbundschema (im Folgenden und im Manuskript als jun-ChS bezeichnet)[5] verwendet, das die CP-Korrektur[4] in jeden Energiebeitrag zusätzlich zu den CP-jB2-Geometrien einbezieht. Der Ausgangspunkt dieses zusammengesetzten Schemas ist die coupled-cluster singles and doubles-Methode, die um die Störungsbehandlung von Dreifachanregungen, CCSD(T), ergänzt wird.[6] Zusammenfassend wurde die CP-korrigierte CCSD(T)/jun-cc-pVTZ-Energie (bewertet in der frozen-core (fc) Näherung) verbessert, indem Korrekturen hinzugefügt wurden, die die Extrapolation zum vollständigen Basissatz (CBS) und die Kern-Valenz-Korrelation (CV) berücksichtigen. Diese beiden Beiträge werden mit Hilfe der Møller-Plesset-Theorie zweiter Ordnung (MP2) berechnet. [7] Der Gesamtausdruck lautet:

$$E_{\mathrm{junChS}} = E(\mathrm{CCSD(T)/junVTZ}) + \Delta E^{\mathrm{MP2}/\infty} + \Delta E_{\mathrm{CV}}^{\mathrm{MP2/wCVTZ}} \qquad (2)$$

wobei die CP-korrigierten fc-MP2-Energien, die in Verbindung mit den Basissätzen jun-cc-pVTZ und jun-cc-pVQZ berechnet wurden, mit Hilfe der n^{-3}-Formel auf das CBS-Limit extrapoliert werden.[8] Der CV-Beitrag wird als Energiedifferenz zwischen CP-korrigierten All-Elektronen- und fc-MP2-Berechnungen, beide im gleichen cc-pwCVTZ-Basissatz, evaluiert.[9] Die Ergebnisse sind in Tabelle S1.1.2 aufgeführt, wo auch die niedrigsten Normalmodenfrequenzen für die Strukturen aller stationären Punkte angegeben sind.

Alle Berechnungen wurden mit der Software Gaussian16 durchgeführt.[10]

S1.2. Rotationsparameter.

Die Gleichgewichts-Rotationskonstanten für die vierzehn Isomere des oben erwähnten 1:1-Benzofuran-Formaldehyd (BZF-FA)-Komplexes wurden direkt aus den Gleichgewichtsgeometrien abgeleitet. Für die Isomere ***I*** und ***IV*** (siehe Haupttext) wurden die Schwingungsgrundzustands-Rotationskonstanten B_0^i auch durch Korrektur der CP-jB2-Gleichgewichts-Rotationskonstanten B_e^i mit Schwingungs-Beiträgen (ΔB_vib^i) auf dem CP-B3-Niveau erhalten:

$$B_0^i = B_\mathrm{e}^i(\mathrm{CP-jB2}) + \Delta B_\mathrm{vib}^i(\mathrm{CP-B3}) \qquad (1)$$

wobei B_e^i die Gleichgewichts-Rotationskonstante in Bezug auf die *i-te* Trägheitsachse (*i* = *a*,*b*,*c*) bezeichnet, so dass $B_\mathrm{e}^a = A_\mathrm{e}$. Einzelheiten zur Auswertung des $\Delta B_\mathrm{vib}^i(\mathrm{CP-B3})$ Termes werden in Abschnitt S3 gegeben.
Für alle Isomere sind die Dipolmomentkomponenten und Gleichgewichts-Rotationskonstanten auf dem CP-jB2-Niveau in Tabelle S1.1.2 angegeben.
Alle Berechnungen wurden mit der Software Gaussian16 durchgeführt.[10]

Tabelle S1.1.1. Die mit CP-jB2 berechneten Strukturen der vierzehn Isomere von BZF-FA zusammen mit ihren kartesischen Koordinaten (Å).

ISOMER	BILD	KARTESISCHE KOORDINATEN (Å)
I	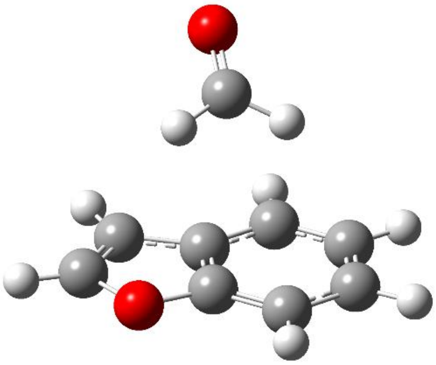	O -0.788793 -1.736892 -0.865408<br>C -0.168213 -2.412177 0.157191<br>C 0.116719 -1.605241 1.204601<br>C -0.355736 -0.295371 0.838709<br>C -0.902907 -0.440041 -0.445319<br>C -1.455634 0.604094 -1.168570<br>C -1.451662 1.850282 -0.552687<br>C -0.913472 2.028598 0.731983<br>C -0.363352 0.969044 1.437938<br>H -0.002804 -3.458636 -0.020410<br>H 0.605654 -1.898870 2.115973<br>H -1.866022 0.452470 -2.155396<br>H -1.871831 2.698495 -1.073093<br>H -0.926968 3.013655 1.175048<br>H 0.056445 1.118794 2.421943<br>C 2.245832 0.533490 -0.829507<br>O 3.256538 0.629645 -0.176564<br>H 1.963891 -0.410090 -1.325115<br>H 1.555170 1.379725 -0.966121
II	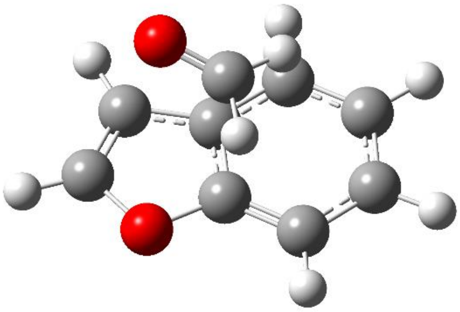	O 0.927556 1.127922 1.168438<br>C 1.622718 1.607887 0.085784<br>C 0.971148 1.390057 -1.078838<br>C -0.245671 0.706366 -0.732478<br>C -0.216658 0.575075 0.664900<br>C -1.213322 -0.045351 1.401168<br>C -2.281847 -0.564093 0.679440<br>C -2.338832 -0.454034 -0.719531<br>C -1.333160 0.178193 -1.436371<br>H 2.565760 2.067429 0.316010<br>H 1.318740 1.662591 -2.058844<br>H -1.155875 -0.120952 2.476598<br>H -3.085136 -1.059324 1.205132<br>H -3.186380 -0.870020 -1.244683<br>H -1.387457 0.256187 -2.512628<br>C 1.165766 -2.142112 0.178527<br>O 2.202346 -1.904833 -0.393124<br>H 1.119394 -2.244663 1.274908<br>H 0.214895 -2.268312 -0.361706
III	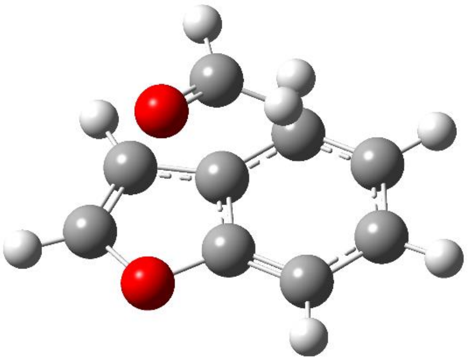	O 0.998179 -1.191258 -1.087216<br>C 1.654628 -1.616179 0.038450<br>C 0.958818 -1.351066 1.168355<br>C -0.248694 -0.691582 0.745189<br>C -0.164981 -0.622747 -0.654863<br>C -1.132906 -0.040042 -1.456181<br>C -2.233351 0.499345 -0.801398<br>C -2.347623 0.449255 0.597692<br>C -1.367863 -0.142715 1.381717<br>H 2.605951 -2.083430 -0.135771<br>H 1.266267 -1.587513 2.171188<br>H -1.027630 -0.003459 -2.529675<br>H -3.016302 0.967465 -1.379874<br>H -3.219511 0.879043 1.069207<br>H -1.467611 -0.177563 2.457245<br>C 1.092075 2.135992 0.467299<br>O 2.073131 2.080153 -0.233779<br>H 0.084297 2.291984 0.053100<br>H 1.156282 2.034371 1.563092

IV	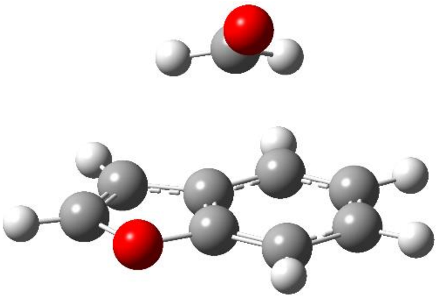	O -0.876261 -1.354998 -1.096978<br>C -1.584127 -1.912107 -0.062495<br>C -1.736088 -1.055192 0.973969<br>C -1.072695 0.162032 0.584954<br>C -0.563467 -0.084049 -0.699370<br>C 0.164692 0.840720 -1.429851<br>C 0.379215 2.072935 -0.824315<br>C -0.118234 2.352617 0.458430<br>C -0.842973 1.410193 1.173969<br>H -1.908855 -2.923532 -0.222795<br>H -2.253474 -1.260426 1.894005<br>H 0.547771 0.608663 -2.411768<br>H 0.943539 2.828922 -1.350089<br>H 0.070406 3.322688 0.894775<br>H -1.219612 1.636048 2.161246<br>C 2.221584 -0.792417 0.750960<br>O 3.265344 -1.074138 0.213883<br>H 1.979444 0.237682 1.054425<br>H 1.455730 -1.555646 0.966851
V	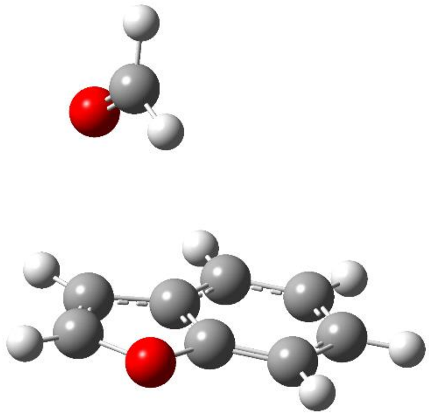	O -1.334115 1.847012 0.364883<br>C -0.175937 2.338946 -0.186183<br>C 0.596062 1.362242 -0.715687<br>C -0.115930 0.130273 -0.493655<br>C -1.294237 0.492647 0.175796<br>C -2.260242 -0.414820 0.578519<br>C -2.011761 -1.750179 0.284491<br>C -0.842310 -2.145545 -0.384752<br>C 0.112895 -1.220118 -0.779747<br>H -0.055696 3.404422 -0.118909<br>H 1.550569 1.486866 -1.194045<br>H -3.155256 -0.096642 1.091282<br>H -2.733962 -2.498857 0.575948<br>H -0.687573 -3.193753 -0.596279<br>H 1.013120 -1.528917 -1.290394<br>C 3.066258 -0.457443 1.042267<br>O 3.419801 -0.383479 -0.109796<br>H 3.775589 -0.708820 1.847328<br>H 2.021732 -0.278715 1.339426
VI	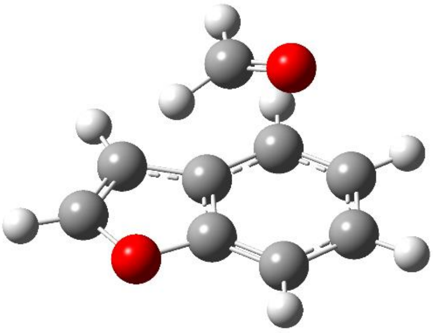	O 1.718117 -0.304808 1.129820<br>C 2.447457 -0.666469 0.025736<br>C 1.873934 -0.247553 -1.126468<br>C 0.669994 0.444349 -0.744334<br>C 0.628590 0.376957 0.656629<br>C -0.389449 0.917260 1.424120<br>C -1.411469 1.551964 0.730938<br>C -1.400649 1.639317 -0.670149<br>C -0.369980 1.093868 -1.419865<br>H 3.348981 -1.213435 0.231489<br>H 2.258318 -0.405904 -2.118286<br>H -0.390221 0.834920 2.500128<br>H -2.236166 1.980315 1.280921<br>H -2.217498 2.137768 -1.171094<br>H -0.372337 1.165296 -2.498125<br>C -1.213364 -2.101898 -0.364911<br>O -2.281392 -1.961639 0.177720<br>H -1.098649 -2.013798 -1.457043<br>H -0.295812 -2.328311 0.201420

VII	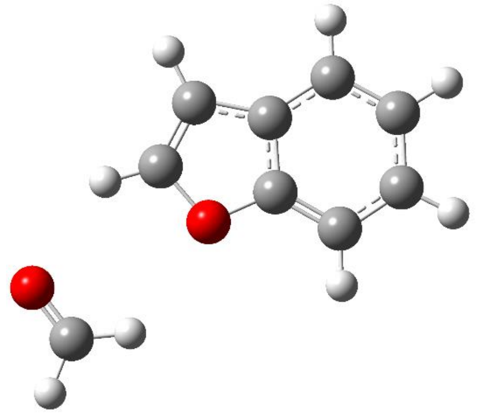	O 0.774716 -0.284833 0.000000<br>C 1.066884 1.061259 0.000000<br>C -0.052760 1.819658 0.000000<br>C -1.163221 0.902795 0.000000<br>C -0.590918 -0.377595 0.000000<br>C -1.327849 -1.549928 0.000000<br>C -2.710793 -1.410414 0.000000<br>C -3.315327 -0.143902 0.000000<br>C -2.557035 1.017971 0.000000<br>H 2.117025 1.292151 0.000000<br>H -0.085703 2.894445 0.000000<br>H -0.850233 -2.518111 0.000000<br>H -3.331907 -2.294230 0.000000<br>H -4.393588 -0.076387 0.000000<br>H -3.033293 1.987936 0.000000<br>C 4.072306 -1.017035 0.000000<br>O 4.262306 0.176386 0.000000<br>H 3.056793 -1.439098 0.000000<br>H 4.911327 -1.731069 0.000000
VIII	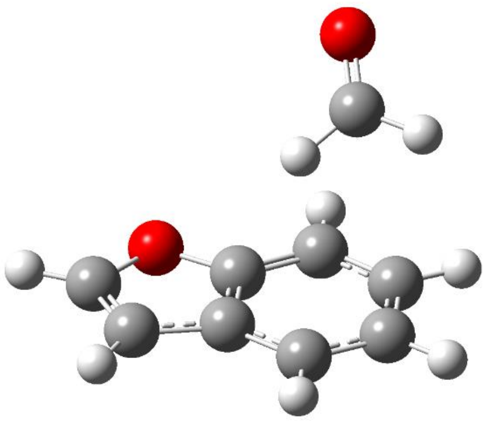	O -1.538086 -1.175056 0.917194<br>C -2.367571 -1.297081 -0.168120<br>C -2.135412 -0.344220 -1.100349<br>C -1.065892 0.467491 -0.581074<br>C -0.741030 -0.094307 0.663373<br>C 0.255933 0.393763 1.491678<br>C 0.948292 1.508467 1.033242<br>C 0.646969 2.096475 -0.205797<br>C -0.352571 1.587573 -1.022724<br>H -3.064848 -2.112945 -0.121365<br>H -2.656128 -0.232109 -2.034365<br>H 0.481649 -0.072955 2.438229<br>H 1.734427 1.929661 1.642727<br>H 1.206298 2.963771 -0.525766<br>H -0.575954 2.048106 -1.974396<br>C 2.594933 -0.763551 -0.770371<br>O 3.084456 -1.740158 -0.257263<br>H 3.175715 0.158662 -0.934613<br>H 1.546524 -0.739743 -1.103831
IX	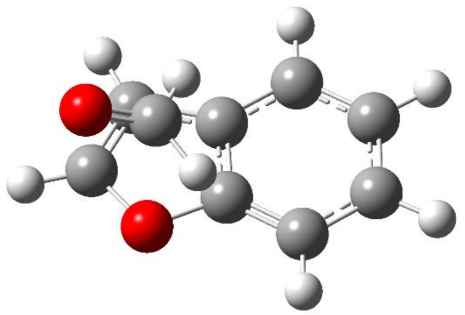	O 0.755940 0.535478 1.270058<br>C 1.105398 1.656679 0.557080<br>C 0.236451 1.913345 -0.447185<br>C -0.761087 0.876326 -0.388852<br>C -0.388916 0.060135 0.689865<br>C -1.090747 -1.067898 1.081852<br>C -2.223574 -1.377488 0.338543<br>C -2.623916 -0.581989 -0.746641<br>C -1.906197 0.544640 -1.120876<br>H 2.002203 2.146830 0.887392<br>H 0.298825 2.730718 -1.142971<br>H -0.773010 -1.667318 1.921614<br>H -2.808300 -2.246507 0.602437<br>H -3.511012 -0.855727 -1.299045<br>H -2.224412 1.149533 -1.957710<br>C 2.314033 -1.306495 -0.597337<br>O 3.470101 -0.967519 -0.661206<br>H 1.536088 -0.850244 -1.229713<br>H 1.974536 -2.090721 0.098670

X

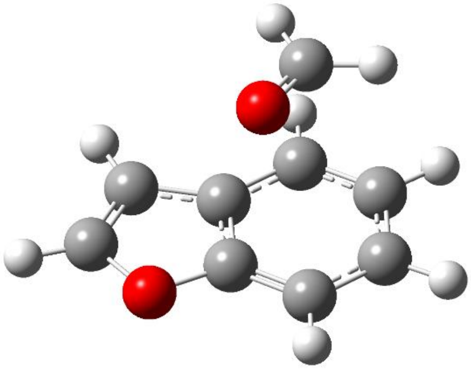

O	-1.795051	0.237404	1.042433
C	-2.486825	0.537107	-0.102112
C	-1.830566	0.139712	-1.216864
C	-0.607752	-0.467222	-0.761643
C	-0.642977	-0.374573	0.637786
C	0.365169	-0.837637	1.466087
C	1.459301	-1.421118	0.840588
C	1.529690	-1.525656	-0.557969
C	0.506080	-1.055097	-1.370246
H	-3.427656	1.033262	0.048348
H	-2.170535	0.263344	-2.229456
H	0.302657	-0.737971	2.538758
H	2.274379	-1.797355	1.441374
H	2.396371	-1.988605	-1.007870
H	0.568673	-1.145974	-2.445475
C	1.959211	1.783475	-0.529396
O	1.324637	2.258933	0.379511
H	2.933454	1.296209	-0.367265
H	1.595933	1.810384	-1.568960

XI

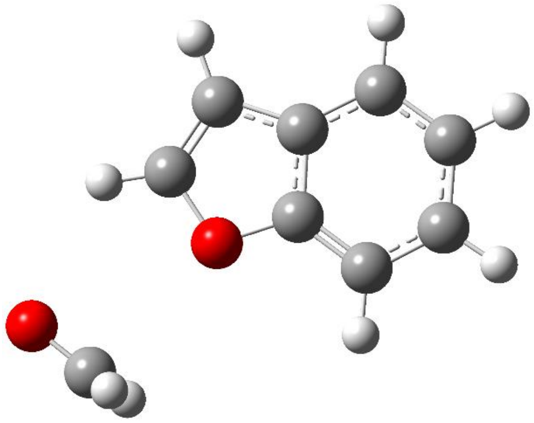

O	-0.875867	-0.112638	0.000000
C	-1.062532	1.250764	0.000000
C	0.111608	1.921125	0.000000
C	1.148232	0.921335	0.000000
C	0.479103	-0.310978	0.000000
C	1.122605	-1.536929	0.000000
C	2.512151	-1.504445	0.000000
C	3.212700	-0.288400	0.000000
C	2.546742	0.928673	0.000000
H	-2.089210	1.567800	0.000000
H	0.226831	2.990186	0.000000
H	0.571998	-2.465548	0.000000
H	3.063149	-2.433545	0.000000
H	4.292907	-0.304547	0.000000
H	3.096487	1.858899	0.000000
C	-3.604148	-1.076892	0.000000
O	-4.130518	0.009479	0.000000
H	-3.351122	-1.599367	0.935252
H	-3.351122	-1.599367	-0.935252

XII

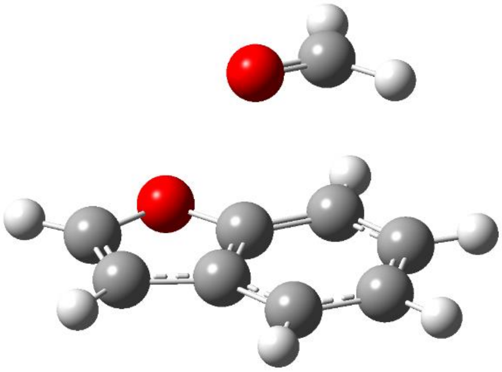

O	1.782223	-0.078733	-1.115983
C	2.476508	-0.580621	-0.042741
C	1.835609	-0.364782	1.128166
C	0.622878	0.334031	0.795167
C	0.647133	0.480730	-0.599533
C	-0.358311	1.097145	-1.325564
C	-1.435861	1.587456	-0.595020
C	-1.488390	1.458899	0.802419
C	-0.470233	0.835413	1.508317
H	3.404492	-1.064322	-0.285065
H	2.174118	-0.670911	2.101513
H	-0.301907	1.192589	-2.399507
H	-2.244788	2.082510	-1.112770
H	-2.341892	1.853581	1.334515
H	-0.525140	0.733572	2.582367
C	-2.023393	-1.732400	-0.547542
O	-1.351339	-2.337593	0.250550
H	-2.936831	-1.194132	-0.250238
H	-1.755878	-1.684171	-1.615086

XIII	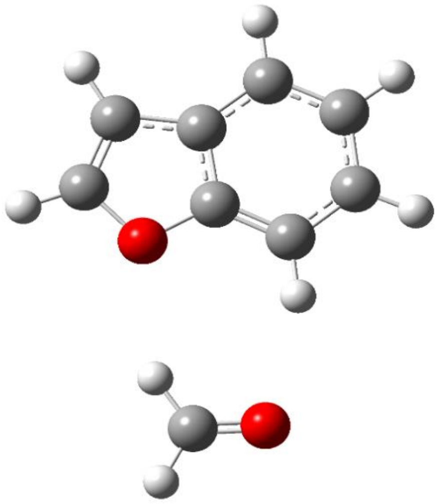	O 0.441276 1.328045 0.000000<br>C -0.479693 2.347349 0.000000<br>C -1.750956 1.886974 0.000000<br>C -1.656453 0.449842 0.000000<br>C -0.284249 0.162516 0.000000<br>C 0.240212 -1.119365 0.000000<br>C -0.686322 -2.155579 0.000000<br>C -2.067742 -1.905496 0.000000<br>C -2.567405 -0.611626 0.000000<br>H -0.066345 3.338981 0.000000<br>H -2.641393 2.489898 0.000000<br>H 1.305730 -1.298580 0.000000<br>H -0.333372 -3.176547 0.000000<br>H -2.753115 -2.740722 0.000000<br>H -3.632490 -0.429427 0.000000<br>C 3.796241 0.333695 0.000000<br>O 3.678145 -0.868878 0.000000<br>H 2.921574 1.000576 0.000000<br>H 4.789233 0.811777 0.000000
XIV	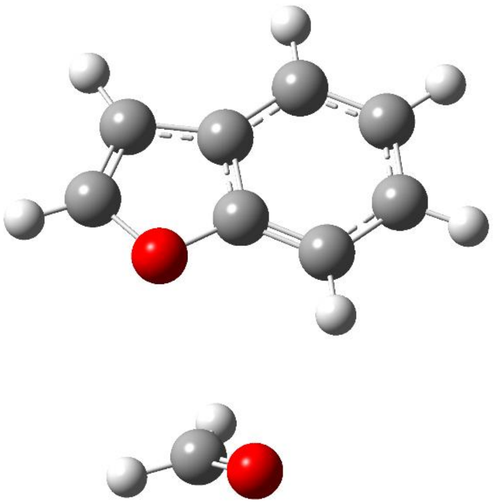	O 0.677946 1.143419 -0.386557<br>C -0.038865 2.293173 -0.159642<br>C -1.332857 2.037979 0.138293<br>C -1.476758 0.605336 0.103289<br>C -0.207834 0.107780 -0.226363<br>C 0.083682 -1.239956 -0.362298<br>C -0.973198 -2.117487 -0.150150<br>C -2.256303 -1.654000 0.180303<br>C -2.523407 -0.299113 0.310068<br>H 0.518978 3.206509 -0.253749<br>H -2.088931 2.772021 0.352479<br>H 1.075410 -1.583182 -0.615430<br>H -0.801932 -3.179959 -0.243324<br>H -3.050017 -2.370350 0.335449<br>H -3.514810 0.047595 0.563957<br>C 3.252809 -0.023132 0.654894<br>O 3.530012 -0.907313 -0.117999<br>H 2.453889 -0.134019 1.403710<br>H 3.786936 0.940297 0.649353

Tabelle S1.1.2 CP-jB2-Gleichgewichts-Rotationskonstanten (A_e, B_e , C_e; in MHz), Absolutwerte der Gleichgewichts-Dipolmomentkomponenten ($|\mu_{e,a}|$, $|\mu_{e,b}|$, $|\mu_{e,c}|$; in Debye), relative Gleichgewichtsenergien (ΔE; in kJ·mol^{-1}) auf den CP-jB2- und jun-ChS-Niveaus, harmonische ZPE-korrigierte Energien (ΔE_0; in kJ·mol^{-1}), die durch Addition der harmonischen CP-jB2-ZPE-Beiträge erhalten werden. Die Wechselwirkungsenergien (IE; in kJ·mol^{-1}) und niedrigsten Normalmodenfrequenzen (LNMF; in cm^{-1}) werden für alle Energieminima und Übergangszustände angegeben.

**MINIMA[a]**												
Isomer	A_e	B_e	C_e	$\|\mu_{e,a}\|$	$\|\mu_{e,b}\|$	$\|\mu_{e,c}\|$	ΔE		ΔE_0		IE[a]	LNMF
							CP-jB2	jun-ChS	CP-jB2[a]	jun-ChS[a]	jun-ChS	
I	1189.40	1123.68	798.64	2.0	0.4	0.3	0.00	0.00	0.00	0.00	-16.15	+23
II	1419.94	1044.06	878.27	1.8	0.2	0.2	0.24	0.15	0.35	0.26	-16.01	+23
III	1373.64	1065.63	879.58	1.8	0.5	1.8	0.24	0.34	0.43	0.53	-15.75	+31
IV	1211.55	1068.19	774.45	2.8	0.7	1.5	0.72	0.83	0.63	0.74	-15.32	+22
V	1463.31	900.34	625.78	0.1	0.1	1.8	1.86	1.59	1.05	0.78	-14.48	+11
VI	1381.13	1045.21	864.11	2.2	0.1	1.6	1.93	2.08	1.79	1.95	-14.07	+28
VII	2883.80	542.31	456.47	0.2	1.5	0.0	2.05	1.84	1.68	1.47	-14.47	+14
VIII	1429.24	866.93	720.62	1.8	2.1	1.4	2.15	2.59	1.69	2.12	-13.51	+4
IX	1691.92	805.34	698.20	2.5	0.2	0.3	2.40	2.19	1.83	1.62	-13.89	+22
X	1425.68	1044.97	894.59	0.7	0.9	2.1	2.64	3.19	2.27	2.82	-12.91	+24
XI	2667.46	606.90	496.12	1.3	1.3	0.0	2.87	2.45	2.36	1.94	-13.57	+16
XII	1394.38	1047.50	878.67	0.7	1.0	0.6	2.92	3.23	2.11	2.42	-12.86	+9
XIII	1625.79	760.93	518.33	0.3	2.6	0.0	3.23	3.15	3.01	2.93	-13.08	+15
XIV	1624.89	848.89	577.87	1.2	2.1	1.6	3.67	3.33	3.44	3.10	-12.67	+17
**ÜBERGANGSZUSTÄNDE**												
I-II	1282.25	1081.27	845.92				0.43	0.39	0.18	0.14		*i* 26
II-III	1487.51	988.75	853.95				1.45	1.67	0.85	1.07		*i* 55
III-IV	1184.64	1129.56	812.73				0.95	1.16	0.65	0.86		*i* 30
I-V	1427.83	948.95	650.72				2.23	2.33	1.41	1.51		*i* 24
IV-VI	1294.90	1061.17	830.66				2.07	2.24	1.52	1.69		*i* 33
VII-XI	2729.88	583.18	482.50				3.20	2.88	2.25	1.93		*i* 52
II-IX	1644.85	837.33	728.40				2.43	2.29	1.51	1.37		*i* 26
VI-X	1410.67	1012.88	875.82				2.97	3.38	2.34	2.75		*i* 26
IX-XI	2251.73	663.03	557.36				3.93	3.76	3.30	3.13		*i* 20

[a] Zur Definition siehe Gleichung (1) in Ref. [5]

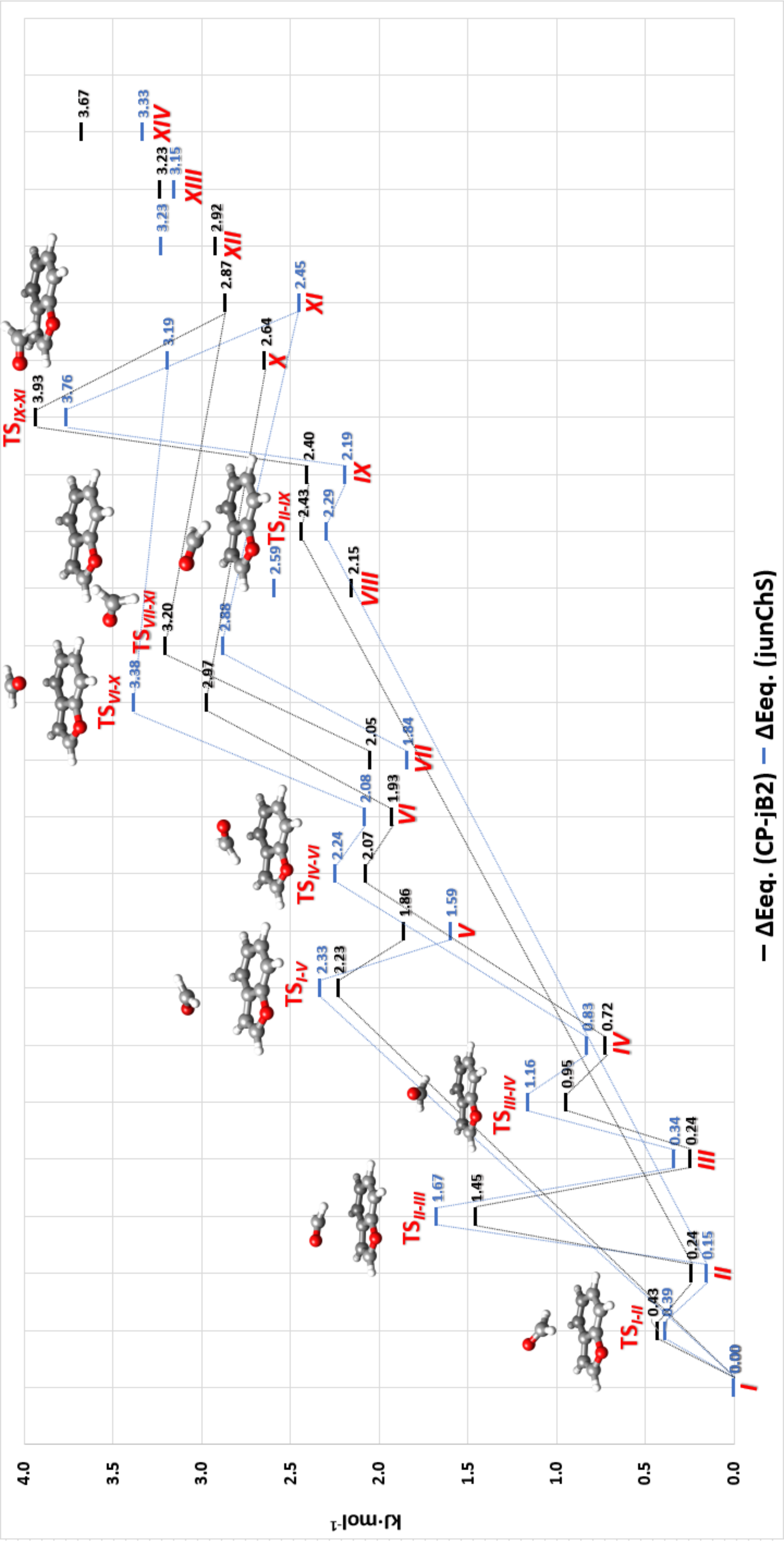

Abbildung SF1.1.1. Darstellung der relativen jun-ChS- und CP-jB2-Gleichgewichtsenergien (ΔE_{eq}; in kJ·mol^{-1}), wie in Tabelle S1.1.2 angegeben. und der Strukturen der Übergangszustände.

S1.3. Natural Bond Orbital (NBO) Analyse.

Für die vierzehn tiefliegenden Minima wurde die Natural Bond Orbital (NBO) Analyse mit dem Programm NBO7[11] über eine Schnittstelle zu Gaussian16[10] auf dem Theorieniveau B3LYP-D3(BJ)/maug-cc-pVTZ-dH[2,12,13] auf der Grundlage der entsprechenden CP-jB2-Geometrien durchgeführt. Für das Isomer ***I*** wurde die Analyse zu Vergleichszwecken auch unter Verwendung der semi-experimentellen Gleichgewichtsstruktur (r_e^{SE}; Abschnitt S3) als Referenzgeometrie durchgeführt. Die Ergebnisse sind in den Tabellen S1.3.1 bis S1.3.14 aufgeführt.

Tabelle S1.3.1. NBO-Ergebnisse für das Isomer ***I*** des BZF-FA-Komplexes.

NBO-Donor	NBO-Akzeptor	E(2) [kJ·mol^{-1}]	
		CP-jB2	r_e^{SE}
BD (2) C2- C3	BD*(1) C16- H18	0.2	0.2
BD (1) C4- C5	RY (3) C16	0.4	0.4
BD (2) C4- C5	BD*(1) C16- O17	4.3	4.0
BD (2) C4- C5	RY (4) C16	0.3	0.3
BD (1) C4- C9	RY (3) C16	0.3	0.2
BD (2) C6- C7	BD*(1) C16- H19	0.6	0.6
BD (2) C8- C9	BD*(1) C16- O17	0.3	0.2
BD (2) C8- C9	BD*(1) C16- H19	0.3	0.3
BD (1) C16- O17	BD*(2) C4- C5	0.3	0.3

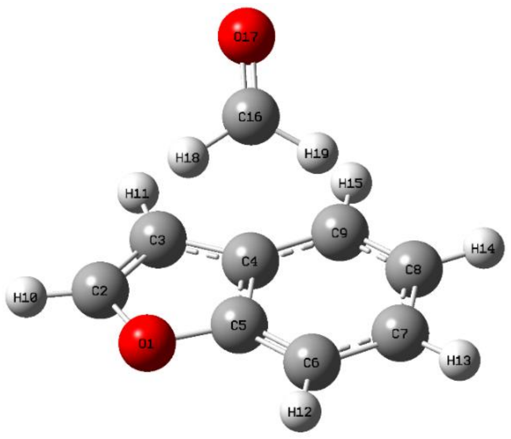

Tabelle S1.3.2. NBO-Ergebnisse für das Isomer ***II*** des BZF-FA-Komplexes.

**NBO-Donor**	**NBO-Akzeptor**	**E(2) [kJ·mol^{-1}]**
BD (2) C2- C3	BD*(1) C16- O17	0.4
BD (1) C4- C5	RY (3) C16	0.2
BD (2) C4- C5	BD*(1) C16- O17	4.1
BD (2) C4- C5	BD*(2) C16- O17	0.4
BD (2) C6- C7	BD*(1) C16- O17	0.5
BD (2) C6- C7	BD*(2) C16- O 17	0.2
BD (2) C8- C9	BD*(1) C16- H19	0.7
BD (2) C16- O17	RY (3) C4	0.5
BD (1) C16- H18	BD*(2) C4- C5	0.6
BD (1) C16- H18	BD*(2) C6- C7	0.3

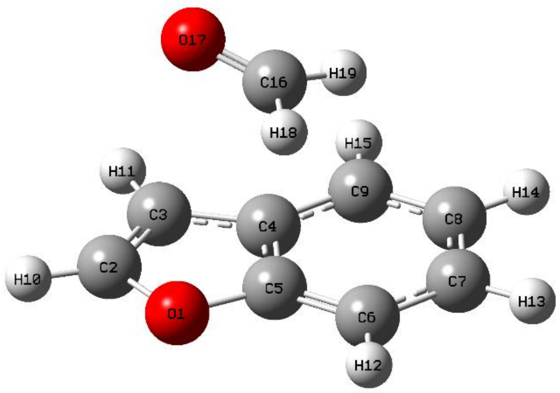

Tabelle S1.3.3. NBO-Ergebnisse für das Isomer ***III*** des BZF-FA-Komplexes.

**NBO-Donor**	**NBO-Akzeptor**	**E(2) [kJ·mol^{-1}]**
LP (2) O1	RY (3) O17	0.3
BD (1) C3- C4	RY (3) C16	0.2
BD (1) C4- C5	RY (3) C16	0.5
BD (2) C4- C5	BD*(1) C16- O17	4.9
BD (2) C4- C5	BD*(2) C16- O17	0.2
BD (1) C4- C9	RY (3) C16	0.3
BD (2) C6- C7	BD*(1) C16- H18	0.8
BD (2) C8- C9	BD*(1) C16- O17	0.2
BD (2) C16- O17	RY (4) C5	0.2
BD (1) C16- H19	BD*(2) C4- C5	0.7

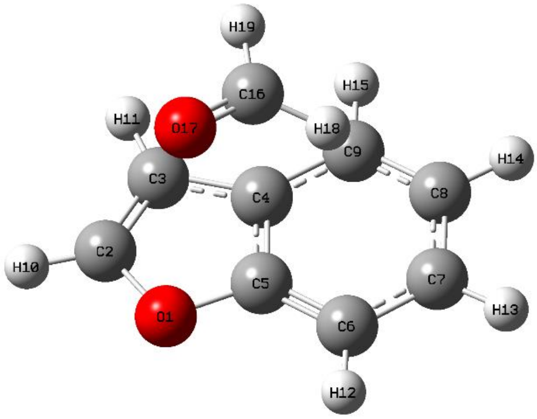

Tabelle S1.3.4. NBO-Ergebnisse für das Isomer ***IV*** des BZF-FA-Komplexes.

NBO-Donor	NBO-Akzeptor	E(2) [kJ·mol^{-1}]
BD (2) C2-C3	BD*(1) C16-H19	0.7
BD (2) C4-C5	BD*(1) C16-O17	2.3
BD (2) C4-C5	BD*(2) C16-O17	0.3
BD (2) C6-C7	BD*(1) C16-O17	0.9
BD (2) C8-C9	BD*(1) C16-H18	0.9
BD (1) C16-O17	BD*(2) C4-C5	0.3

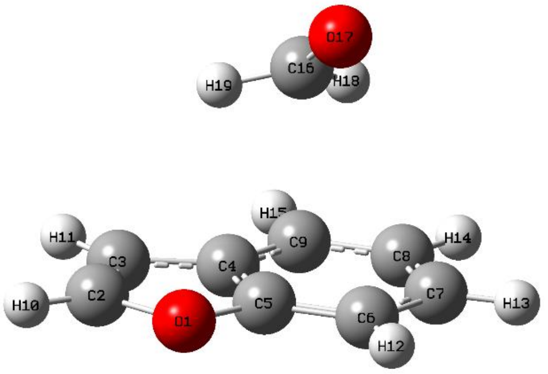

Tabelle S1.3.5. NBO-Ergebnisse für das Isomer ***V*** des BZF-FA-Komplexes.

NBO-Donor	NBO-Akzeptor	E(2) [kJ·mol^{-1}]
BD (2) C4-C5	BD*(1) C16- H19	0.4
LP (2) O17	BD*(1) C2-C3	0.3
LP (2) O17	BD*(2) C2-C3	0.4
BD (1) C16-O17	BD*(2) C8-C9	0.3
BD (2) C16-O17	RY (2) C3	0.3
BD (2) C16-O17	RY (1) H15	0.2
BD (1) C16-H19	BD*(2) C2-C3	0.5
BD (1) C16-H19	BD*(2) C4-C5	0.8
BD (1) C16-H19	BD*(2) C8-C9	0.4

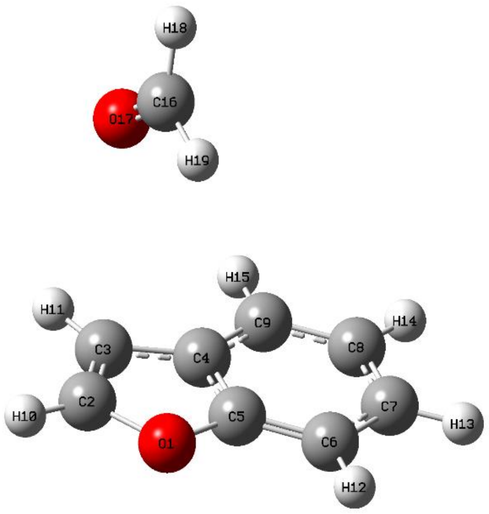

Tabelle S1.3.6. NBO-Ergebnisse für das Isomer ***VI*** des BZF-FA-Komplexes.

NBO-Donor	NBO-Akzeptor	E(2) [kJ·mol^{-1}]
BD (2) C2-C3	BD*(1) C16-H19	0.3
BD (1) C4-C5	RY (3) C16	0.3
BD (2) C4-C5	BD*(1) C16-O17	5.7
BD (2) C8-C9	BD*(1) C16-O17	0.3
BD (2) C16-O17	RY (3) C6	0.3
BD (1) C16-H18	BD*(2) C4-C5	0.8
BD (1) C16-H18	BD*(2) C8-C9	0.3

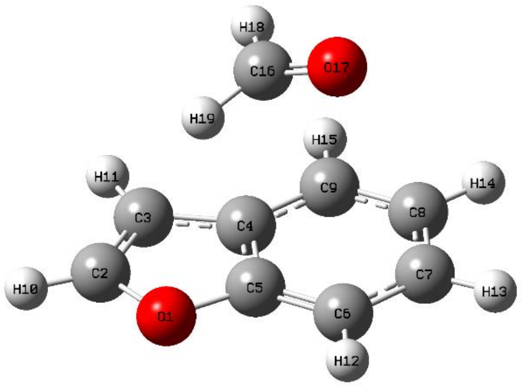

Tabelle S1.3.7. NBO-Ergebnisse für das Isomer ***VII*** des BZF-FA-Komplexes.

NBO-Donor	NBO-Akzeptor	E(2) [kJ·mol^{-1}]
LP (1) O1	BD*(1) C16-H18	2.1
LP (1) O1	RY (1) H18	0.3
BD (1) C2-H10	BD*(2) C16-O17	0.2
LP (1) O17	BD*(1) C2-H10	1.8
LP (2) O17	BD*(1) C2-C3	0.4
LP (2) O17	BD*(1) C2-H10	4.1
BD (2) C16-O17	RY (1) C2	0.4
BD (2) C16-O17	RY (3) C2	0.2

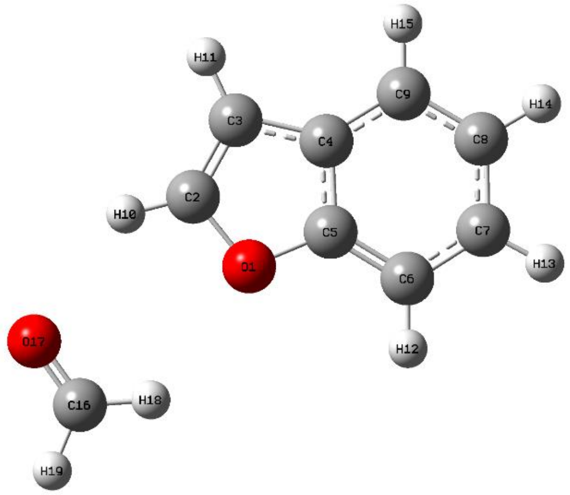

Tabelle S1.3.8. NBO-Ergebnisse für das Isomer ***VIII*** des BZF-FA-Komplexes.

NBO-Donor	NBO-Akzeptor	E(2) [kJ-mol^{-1}]
BD (2) C4-C5	BD*(1) C16-H19	0.8
BD (1) C6-C7	RY (3) C16	0.3
BD (2) C6-C7	BD*(1) C16-O17	3.8
BD (1) C7-C8	RY (3) C16	0.2
BD (2) C8-C9	BD*(2) C16-O17	0.3
BD (1) C16-H18	BD*(2) C6-C7	0.3
BD (1) C16-H18	BD*(2) C8-C9	0.3

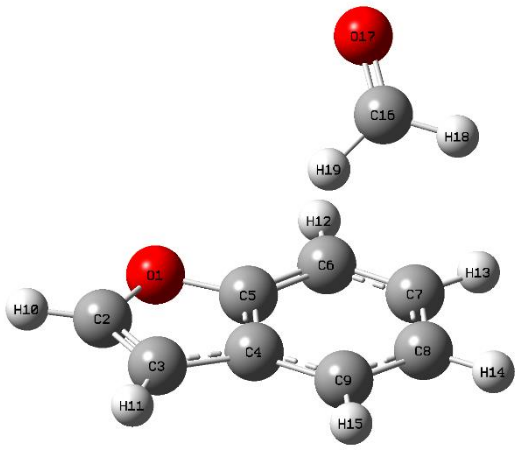

Tabelle S1.3.9. NBO-Ergebnisse für das Isomer ***IX*** des BZF-FA-Komplexes.

NBO-Donor	NBO-Akzeptor	E(2) [kJ·mol^{-1}]
LP (1) O1	BD*(1) C16-O17	0.4
LP (2) O1	BD*(1) C16-O17	2.5
BD (1) O1-C2	RY (3) C16	0.3
BD (1) O1-C5	RY (3) C16	0.2
BD (2) C2-C3	BD*(1) C16-O17	0.4
BD (2) C4-C5	BD*(1) C16-O17	0.4
BD (2) C4- C5	BD*(1) C16-H18	0.6
BD (1) C16-O17	BD*(2) C2-C3	0.4
BD (1) C16-H18	BD*(2) C4-C5	0.3
BD (1) C16-H19	BD*(2) C4-C5	0.4

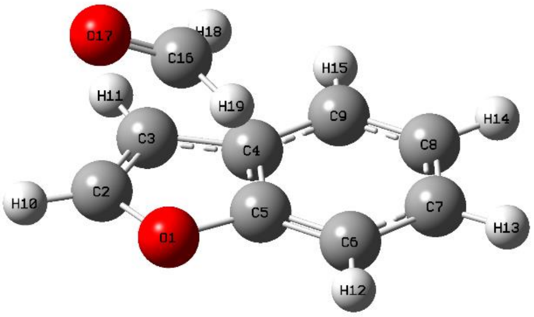

Tabelle S1.3.10. NBO-Ergebnisse für das Isomer ***X*** des BZF-FA-Komplexes.

NBO-Donor	NBO-Akzeptor	E(2) [kJ·mol^{-1}]
LP (2) O1	RY (3) O17	0.3
BD (1) C8-C9	RY (3) C16	0.2
BD (2) C8-C9	BD*(1) C16-O17	4.9
BD (2) C16-O17	RY (1) C4	0.3
BD (2) C16- O17	RY (3) C4	0.5
BD (2) C16-O17	RY (3) C6	0.3
BD (1) C16-H18	BD*(2) C8-C9	0.4
BD (1) C16-H19	BD*(2) C8-C9	0.5

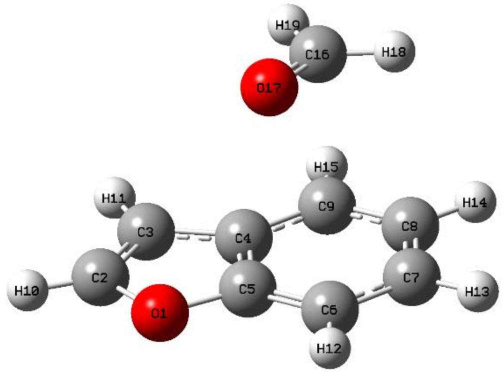

Tabelle S1.3.11. NBO-Ergebnisse für das Isomer ***XI*** des BZF-FA-Komplexes.

NBO-Donor	NBO-Akzeptor	E(2) [kJ·mol^{-1}]
LP (1) O1	BD*(1) C16-O17	0.7
LP (1) O1	BD*(2) C16-O17	3.5
LP (1) O1	RY (1) H18	0.3
LP (1) O1	RY (1) H19	0.3
BD (1) O1-C5	RY (3) C16	0.7
BD (1) C2-H10	BD*(2) C16-O17	0.4
LP (1) O17	BD*(1) C2-H10	0.8
BD (1) C16-O17	RY (1) C2	0.4
BD (1) C16-O17	RY (3) C2	0.3
BD (1) C16-O17	RY (1) H10	0.3
BD (2) C16-O17	BD*(1) O1-C2	0.3
BD (2) C16-O17	BD*(1) O1-C5	0.5
BD (2) C16-O17	BD*(1) C2-C3	0.5
BD (2) C16- O17	BD*(1) C2-H10	0.6

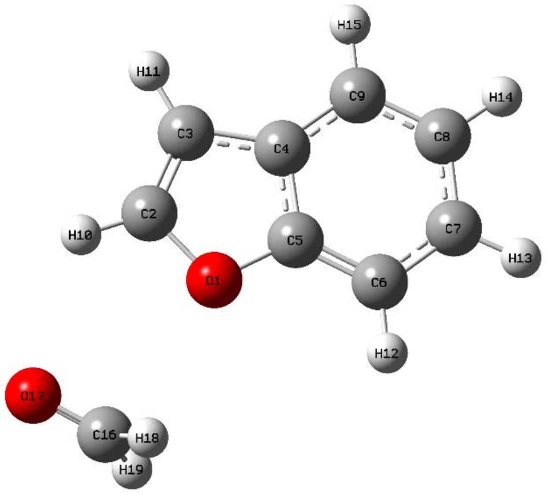

Tabelle S1.3.12. NBO-Ergebnisse für das Isomer ***XII*** des BZF-FA-Komplexes.

NBO-Donor	NBO-Akzeptor	E(2) [kJ·mol^{-1}]
BD (1) C6-C7	RY (3) C16	0.2
BD (2) C6-C7	BD*(1) C16-O17	3.8
BD (2) C16-O17	RY (1) C4	0.3
BD (2) C16-O17	RY (3) C4	0.5
BD (2) C16-O17	RY (4) C5	0.2
BD (1) C16-H18	BD*(2) C6-C7	0.4
BD (1) C16-H18	BD*(2) C8-C9	0.2
BD (1) C16-H19	BD*(2) C6-C7	0.3

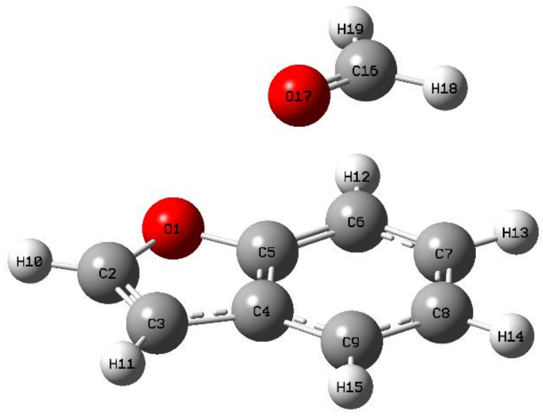

Tabelle S1.3.13. NBO-Ergebnisse für das Isomer ***XIII*** des BZF-FA-Komplexes.

NBO-Donor	NBO-Akzeptor	E(2) [kJ·mol^{-1}]
LP (1) O1	BD*(1) C16-H18	3.5
LP (1) O1	RY (1) H18	0.5
BD (1) C6-H12	BD*(2) C16-O17	0.2
LP (1) O17	BD*(1) C6-H12	1.8
LP (1) O17	RY (8) C6	0.3
LP (2) O17	BD*(1) C6-H12	5.1
LP (2) O17	RY (1) H12	0.2
LP (2) O17	RY (6) H12	0.3
BD (2) C16-O17	RY (2) C6	0.3
BD (2) C16-O17	RY (1) H12	0.4

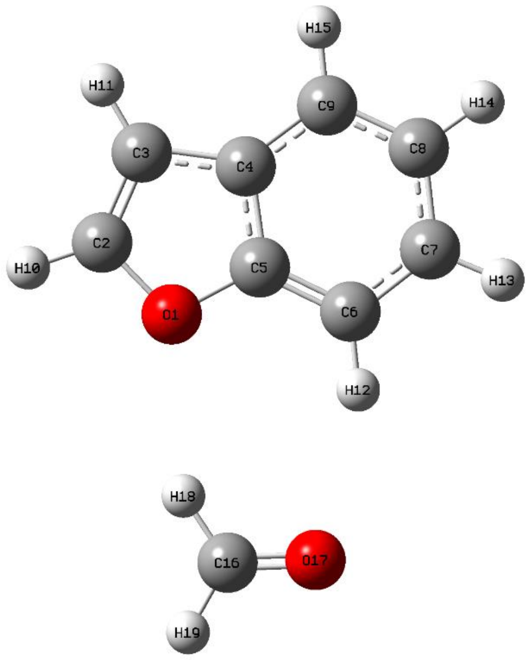

Tabelle S1.3.14. NBO-Ergebnisse für das Isomer ***XIV*** des BZF-FA-Komplexes.

NBO-Donor	NBO-Akzeptor	E(2) [kJ·mol^{-1}]
LP (1) O1	BD*(1) C16-O17	2.7
LP (1) O1	RY (3) C16	0.3
LP (2) O1	BD*(1) C16-O17	1.3
LP (2) O1	RY (2) H18	0.2
LP (1) O17	BD*(1) C6-H12	0.5
LP (2) O17	BD*(1) C6-H12	1.3
BD (1) C16-O17	BD*(1) C6-C7	0.2
BD (1) C16-O17	BD*(1) C6-H12	0.5
BD (2) C16-O17	RY (1) C6	0.2
BD (2) C16-O17	RY (2) C6	0.3
BD (2) C16-O17	RY (1) H12	0.5
BD (1) C16-H18	BD*(2) C6-C7	0.3

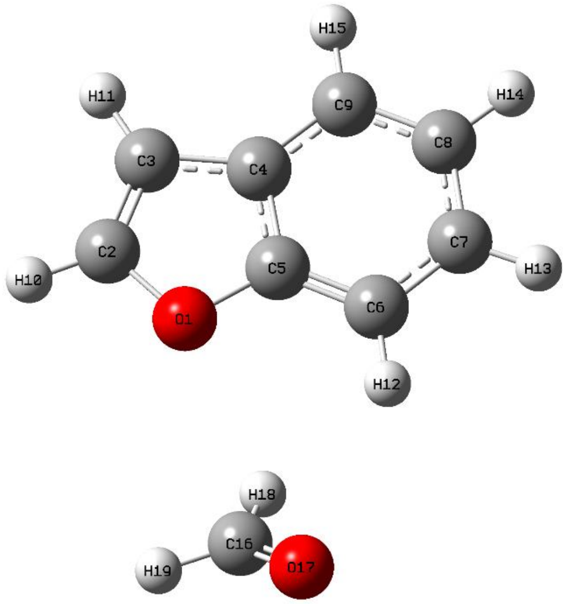

S1.4. Natural Energy Decomposition Analysis (NEDA).

Für die vierzehn tiefliegenden Minima wurde die Natural Energy Decomposition Analysis (NEDA) mit dem Programm NBO7[11] über eine Schnittstelle zu Gaussian16[10] auf dem Theorieniveau B3LYP-D3(BJ)/maug-cc-pVTZ-dH[2,12,13] zusätzlich zu den entsprechenden CP-jB2-Geometrien durchgeführt. Die Ergebnisse sind in Tabelle S1.4.1 aufgeführt und in Abbildung SF1.4.1 grafisch dargestellt. Für Isomer ***I*** sind die NEDA-Ergebnisse, beruhend auf den CP-jB2- und den semi-experimentellen Gleichgewichtsstrukturen ($r_{\mathrm{e}}^{\mathrm{SE}}$, Abschnitt S3), in Tabelle S1.4.2 zusammengestellt und in Abbildung SF1.4.2 zu Vergleichszwecken dargestellt.

Tabelle S1.4.1. NEDA-Ergebnisse (in kJ·mol^{-1}) auf den CP-jB2-Geometrien.

ISOMER	ELEKTRISCH	LADUNGSÜBERTRAGUNG	CORE	GESAMT [a]
I	-22.6	-18.3	24.2	-16.7
II	-25.1	-22.6	31.3	-16.4
III	-24.7	-21.9	30.3	-16.4
IV	-20.8	-17.9	22.9	-15.9
V	-22.8	-15.5	23.9	-14.3
VI	-22.0	-20.8	28.1	-14.6
VII	-24.8	-19.0	30.2	-13.6
VIII	-20.3	-15.7	21.6	-14.5
IX	-20.1	-18.5	25.0	-13.7
X	-24.5	-21.7	32.4	-13.8
XI	-28.7	-20.0	36.0	-12.7
XII	-22.0	-19.2	27.7	-13.5
XIII	-22.9	-22.4	32.8	-12.6
XIV	-24.2	-20.1	32.2	-12.2

[a] Jeder Beitrag (einschließlich des Gesamtenergiebeitrags) wurde auf den nächstliegenden Dezimalwert der erhaltenen Daten gerundet. Die Summe der gerundeten Terme der einzelnen Isomere kann daher von der angegebenen Gesamtenergie abweichen (maximale Abweichung 0,1 kJ·mol^{-1}).

Tabelle S1.4.2. NEDA-Ergebnisse (in kJ·mol^{-1}) für das Isomer ***I*** auf den CP-jB2- und semi-experimentellen Gleichgewichtsstrukturen ($r_{\mathrm{e}}^{\mathrm{SE}}$).

ISOMER *I*	ELEKTRISCH	LADUNGSTRANSFER	CORE	GESAMT
CP-jB2	-22.6	-18.3	24.2	-16.7
$r_{\mathrm{e}}^{\mathrm{SE}}$	-21.3	-17.6	22.3	-16.6

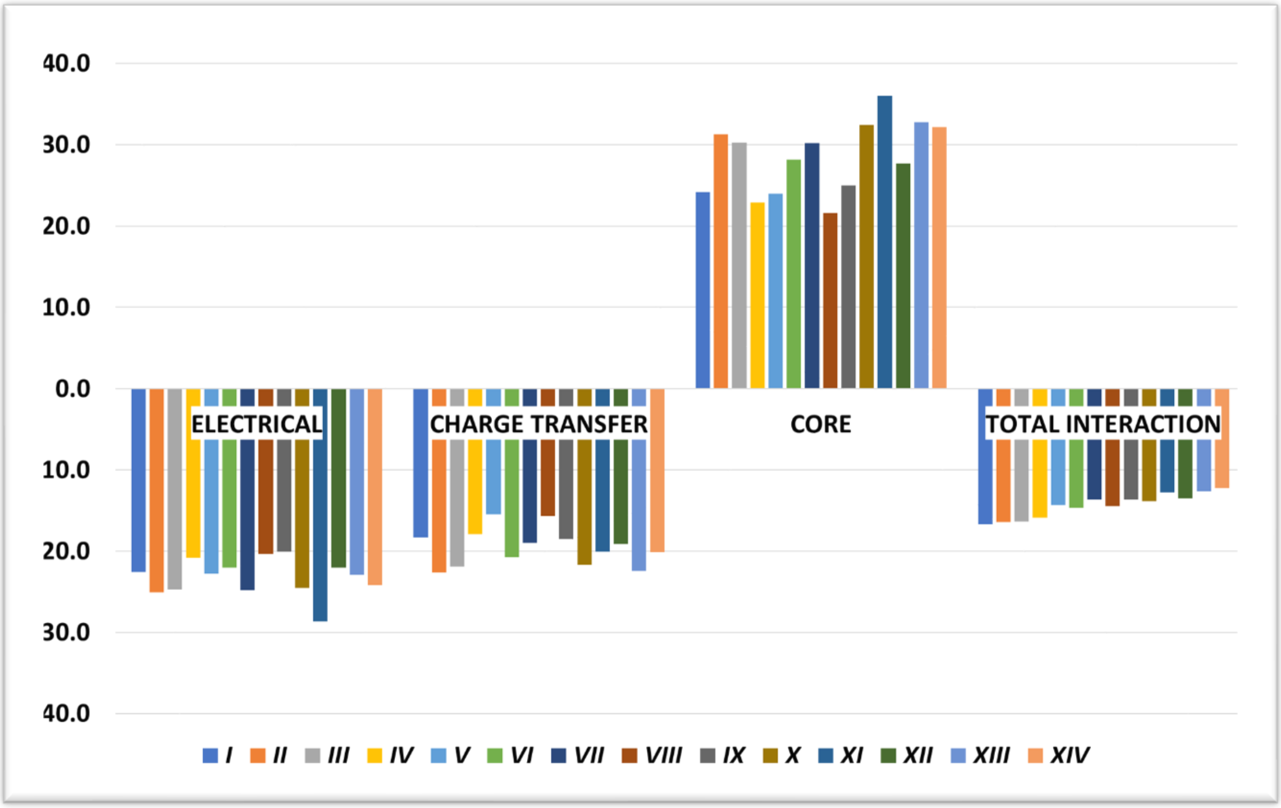

Abbildung SF1.4.1. Darstellung der NEDA-Ergebnisse auf den CP-jB2-Geometrien. Die Farblegende für die verschiedenen Isomere ist darunter angegeben.

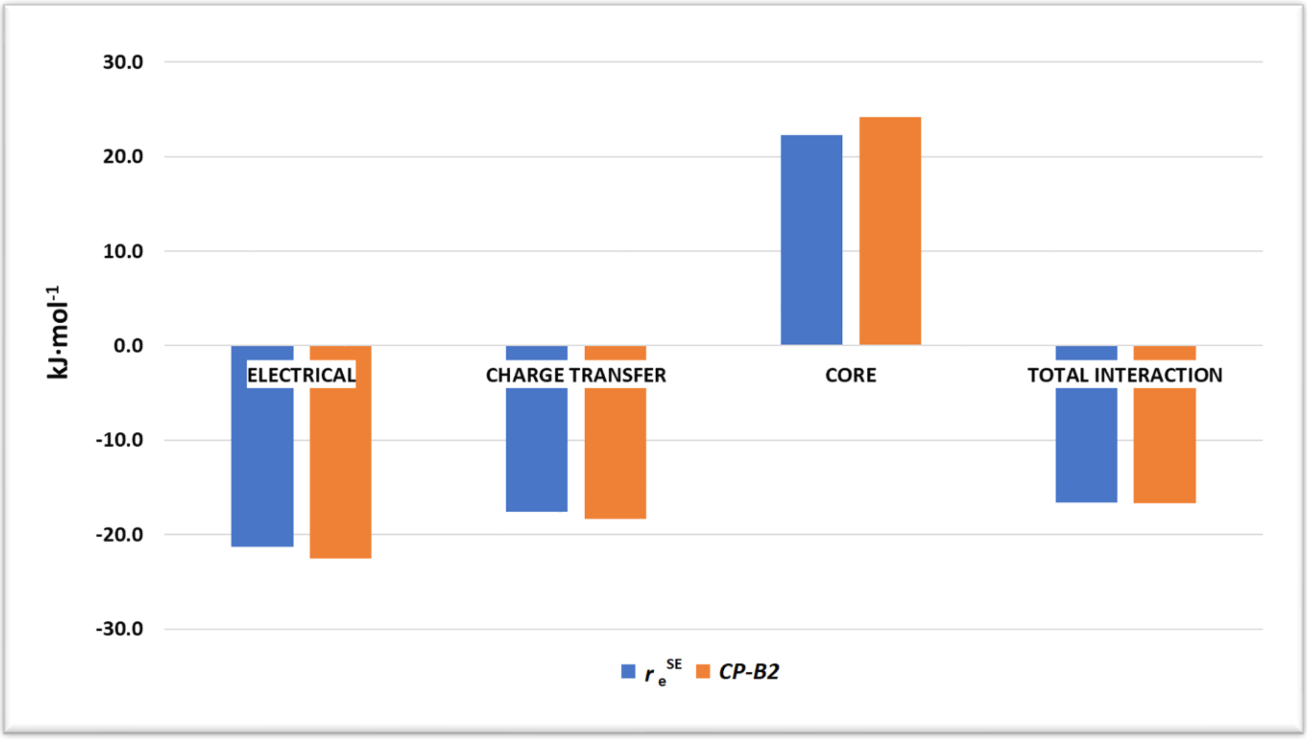

Abbildung SF1.4.2. Darstellung der NEDA-Ergebnisse für das Isomer ***I*** auf den CP-jB2- und semi-experimentellen Gleichgewichtsstrukturen (r_e^{SE}).

S1.5. Symmetry Adapted Perturbation Theory (SAPT) Analyse.

Unter Verwendung der CP-jB2 optimierten Geometrien der vierzehn tiefliegenden Minima wurde die Zerlegungsanalyse der Wechselwirkungsenergie mit dem Ansatz der symmetry adapted perturbation theory (SAPT) [14] durchgeführt. Im Einzelnen wurde die Analyse auf dem "Goldstandard" SAPT2+(3)δMP2/aug-cc-pVTZ Theorieniveau (siehe ref.14b für Details) unter Verwendung des PSI4 Programmpakets durchgeführt.[15] Die gesamte Wechselwirkungsenergie (E_{tot}) und ihre Zerlegung in die Terme Elektrostatik (E_{elec}), Dispersion (E_{disp}), Induktion (E_{ind}) und Austausch-Abstoßung (E_{ex}) sind in Tabelle S1.5.1 aufgeführt.

Tabelle S1.5.1. Ergebnisse der SAPT2+(3)δmp2/aug-cc-pVTZ-Berechnungen für die BZF-FA-Cluster. Werte in kJ·mol⁻¹.

Isomer	E_{elec}	E_{ex}	E_{ind}	E_{disp}	E_{tot}[a]
I	−11.8(33%)[b]	19.7	−3.4(9%)	−20.9(58%)	−16.3
II	−11.4(29%)	23.0	−4.8(12%)	−23.1(59%)	−16.3
III	−11.4(29%)	22.9	−4.5(12%)	−23.0(59%)	−16.0
IV	−10.9(32%)	18.9	−3.2(9%)	−20.2(59%)	−15.5
V	−12.2(41%)	15.7	−2.1(7%)	−15.7(52%)	−14.3
VI	−8.8(25%)	21.0	−4.3(12%)	−22.2(63%)	−14.3
VII	−15.8(51%)	16.4	−3.0(10%)	−11.9(39%)	−14.3
VIII	−10.5(34%)	17.7	−3.1(10%)	−17.5(56%)	−13.5
IX	−9.5(32%)	15.4	−2.4(8%)	−17.6(60%)	−14.1
X	−9.0(25%)	22.5	−4.7(13%)	−22.0(62%)	−13.2
XI	−14.4(46%)	17.7	−3.4(11%)	−13.4(43%)	−13.6
XII	−8.3(25%)	19.8	−3.9(12%)	−20.6(63%)	−13.1
XIII	−14.7(48%)	18.0	−3.3(11%)	−12.9(42%)	−12.9
XIV	−12.3(41%)	17.4	−2.7(9%)	−15.0(50%)	−12.6

[a] Jeder Beitrag (einschließlich des Gesamtenergiebeitrags) wurde auf den nächstliegenden Dezimalwert der erhaltenen Daten gerundet. Die Summe der gerundeten Terme der einzelnen Isomere kann daher von der angegebenen Gesamtenergie abweichen (maximale Abweichung 0,1 kJ·mol⁻¹).

[b] Die Werte in Klammern geben den prozentualen Anteil an der gesamten Wechselwirkungsenergie an.

S1.6. Analyse der nicht-kovalenten Wechselwirkungen im PDB:3HPT YET 2.D Ligandenmodell.

Wir haben die Rolle der π-π*-Wechselwirkungen bei der Stabilisierung von intermolekularen Komplexen in der Gasphase und im festen Zustand untersucht, indem wir die NEDA- und SAPT-Ergebnisse für den BZF-FA-Komplex und das BZF-Derivat des YET-2.D-Liganden (PDB:3HPT) vergleichen.[16,17] Im letzteren Fall wurde der repräsentativste Teil des Kristalls ausgewählt (Abbildung SF1.6.1). Das Fragment 2MeBZF-ACET (Tabelle S1.6.1) besteht aus dem BZF-Derivat 2-Methylbenzofuran und der Acetamidgruppe der Peptidkette (Peptidbindung zwischen Fragment TRP 215.D und GLY 216.D). In Tabelle S1.6.1 sind die kartesischen Koordinaten aus der PDB[16,17] sowie die der Wasserstoffatome aufgeführt, die zur Vervollständigung der Wertigkeit der terminalen Atome von 2MeBZF-ACET hinzugefügt wurden. Für 2MeBZF-ACET wurden die NBO-, NEDA- und SAPT-Analysen auf dem Theorieniveau wie für den BZF-FA-Komplex durchgeführt (Tabellen S1.6.2 und S1.6.3).

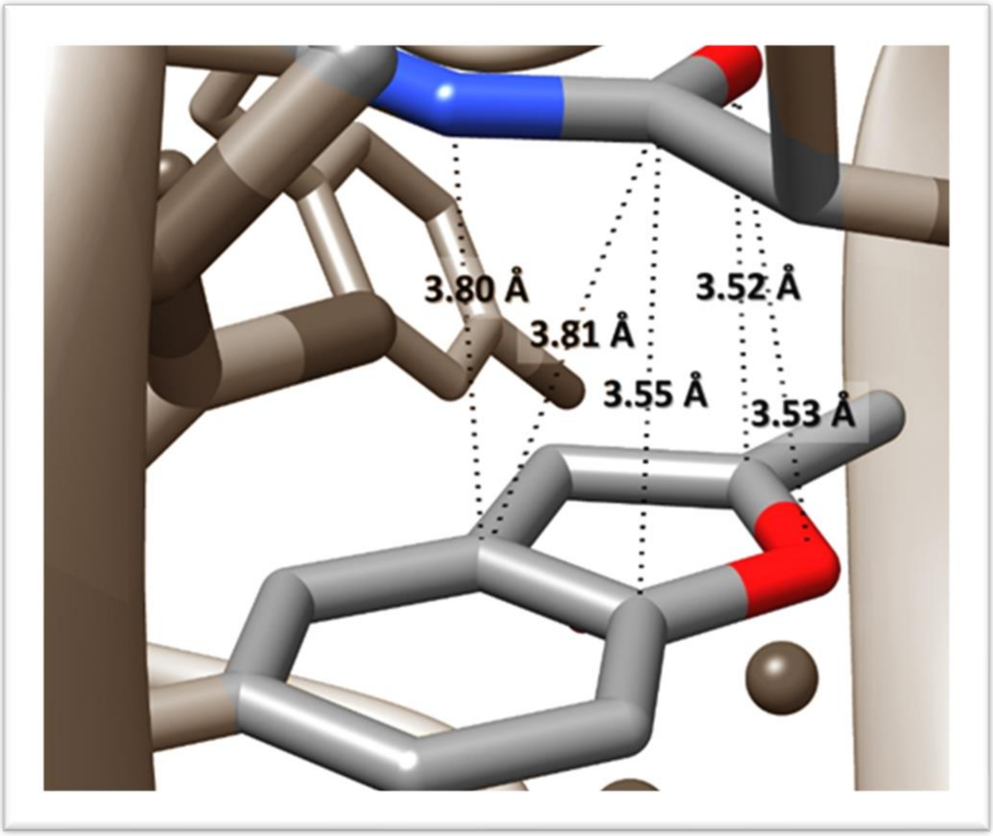

Abbildung SF1.6.1. Teil des PDB:3HPT, der bei den Bindungsanalysen berücksichtigt wurde. Für die grafische Darstellung wurden die Programme Chimera 1.14[18] und GaussView verwendet.

Tabelle S1.6.1. Kartesische Koordinaten (Å) und schematische Darstellung von 2MeBZF-ACET im Kristall.

ATOM	X	Y	Z
C	2.282685	-1.742834	1.153684
C	1.728642	-1.765113	-0.267936
O	0.758406	-2.472842	-0.564997
H	2.309041	-0.701675	1.474579
N	2.359124	-0.967650	-1.130771
H	3.168432	-0.454064	-0.811629
H	1.636739	-2.320655	1.815402
H	3.290119	-2.158886	1.164367
H	2.034528	-0.879038	-2.083373
C	0.910236	2.743514	-0.536161
C	1.125124	2.439011	0.821211
C	-0.071450	2.052992	-1.261589
C	-0.812573	1.055738	-0.618609
C	-0.552137	0.775552	0.705774
C	0.402516	1.435230	1.462571
O	-1.400704	-0.259137	1.139680
C	-2.146964	-0.578452	0.041427
C	-3.188401	-1.651838	0.125977
C	-1.867652	0.186331	-1.111550
H	1.865459	2.995476	1.376744
H	-0.252733	2.287885	-2.300271
H	0.578182	1.183532	2.498600
H	-2.315752	0.138927	-2.093000
H	-3.657180	-1.627509	1.109583
H	-3.945136	-1.484622	-0.641351
H	-2.721803	-2.624142	-0.030108
H	1.500969	3.507787	-1.018477

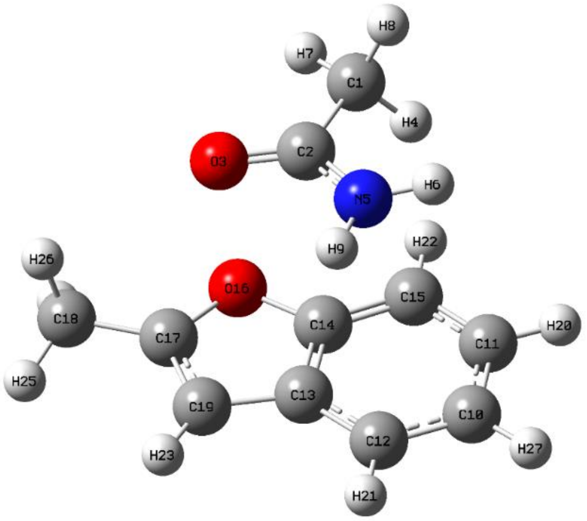

Tabelle S1.6.2. NBO-Ergebnisse für die 2MeBZF-ACET-Struktur.

NBO-Donor	NBO-Akzeptor	E(2) [$kJ\cdot mol^{-1}$]
BD (1) C1- H4	BD*(2) C11-C15	0.8
BD (2) C11- C15	BD*(1) C1-H4	1.2
BD (2) C13-C14	BD*(2) C2-O3	0.8

Tabelle S1.6.3. Vergleich der NBO-, NEDA- und SAPT-Ergebnisse für die Strukturen von Isomer ***I***, Isomer ***II*** und 2MeBZF-ACET. Werte in kJ·mol^{-1}.

	NBO (C=C···C=O)	NEDA				SAPT				
	**E(2)**	**EL[a]**	**CT[a]**	**CO[a]**	**IE[a]**	**ELST[a]**	**IND[a]**	**DISP[a]**	**EXC[a]**	**TI[a]**
**Isomer *I***	4.3	-22.6	-18.3	24.2	-16.7	−11.8	−3.4	−20.9	19.7	−16.3
**Isomer *II***	4.1	-25.1	-22.6	31.3	-16.4	−11.4	−4.8	−23.1	23.0	−16.3
**Kristall-Modell**	0.8	-21.5	-18.9	21.4	-19.1	−6.5	−3.1	−25.4	15.4	−19.5

[a]Die Bezeichnungen EL, CT, CO, IE, ELST, IND, DISP, EXC und TI stehen für "elektrisch", "Ladungstransfer", "Kern", "Elektrostatik", "Induktion", "Dispersion", "Austausch" und "Gesamtwechselwirkung", wie in den entsprechenden Analysemethoden definiert. Jeder Beitrag (einschließlich der Gesamtwechselwirkungsenergie) wurde auf den nächsthöheren Dezimalwert der erhaltenen Daten gerundet. Die Summe der gerundeten Terme der einzelnen Isomere kann daher von der angegebenen Gesamtenergie abweichen (maximale Abweichung 0,1 kJ·mol^{-1}).

S2. Experimentelle Einzelheiten

S2.1. Beschreibung des Versuchsaufbaus und des Zuordnungsverfahrens.

Die Rotationsspektren wurden mit einem Jet-Fourier-Transform-Mikrowellen-Spektrometer (FTMW) des Typs COBRA (Coaxially oriented beam-resonator)[19] an der Universität Chongqing[20] gemessen. Es deckt den Bereich von 2-20 GHz ab und wird mit der FTMW++-Programmumgebung betrieben.[21] Die abgeschätzte Unsicherheit der vorliegenden Frequenzmessungen beträgt 3 kHz.
BZF und Polyformaldehyd (PFA) wurden von Sigma-Aldrich bezogen und ohne weitere Aufbereitung mit Helium als Trägergas bei einem Druck von 3 bar verwendet. PFA und BZF befanden sich in zwei verschiedenen in die Gaszuleitung eingefügten Behältern. Während BZF bei Raumtemperatur gehalten wurde, wurde PFA auf 355 K erhitzt, um das Gleichgewicht von PFA-Oligomeren mit 8-100 Einheiten zu den Formaldehyd (FA)-Monomeren zu verschieben. Das Gasgemisch wurde durch ein Magnetventil (General Valve, Serie 9, Düsenöffnung 0,5 mm) in den Resonator expandiert, um die Molekülkomplexe zu erzeugen.
Durch den instrumentellen Dopplereffekts des sich koaxial zum TEM_{00q}-Modus des Fabry-Perot-Resonators ausbreitenden Strahls erscheint jeder Rotationsübergang als Duplett dessen Frequenz sich als arithmetisches Mittel der Komponenten ergibt.
Die erste spektrale Untersuchung wurde im Frequenzbereich von 8-10 GHz durchgeführt, um die μ_a-Typ Übergänge für die ersten vier niederenergetischen Isomere zu erfassen. Jedoch wurde nur eine Spezies entdeckt. Nach der anfänglichen Zuordnung der Übergänge $5_{0,5}\leftarrow4_{0,4}$, $5_{1,5}\leftarrow4_{1,4}$, $6_{0,6}\leftarrow5_{0,5}$ und $6_{1,6}\leftarrow 5_{1,5}$ vom Typ μ_a wurden insgesamt 204 Rotationsübergänge mit dem SPFIT-Programm von Pickett[22] unter Verwendung des *S-reduzierten* halbstarren Hamiltonians von Watson in seiner III^l-Darstellung erfolgreich angepasst.[23]
Die meisten Übergänge weisen ein schmales Dublett mit einem Intensitätsverhältnis von 1:3 auf, wobei die entsprechenden Zustände als $\upsilon = 0$ bzw. $\upsilon = 1$ bezeichnet werden. Dieses Intensitätsverhältnis ist auf den Austausch der Wasserstoffatome (Kernspin I=1/2) von FA aufgrund der Rotation um seine C_2-Achse zurückzuführen. Eine Reihe von Rotationsparametern (Rotationskonstanten und alle fünf quartären Zentrifugalverzerrungskonstanten) wurde für jeden Zustand gut bestimmt, was in einem RMS der Mikrowellenmessungen von 2,7 kHz resultierte.
Der Vergleich der experimentell bestimmten Rotationsparameter (Tabelle S2.2.1a) mit den berechneten Gleichgewichtswerten (Tabelle S1.1.2) erlaubt keine eindeutige Zuordnung des Spektrums zu einem bestimmten Isomer, reduziert aber die Kandidaten auf die Isomere ***I*** und ***IV***, die ähnliche berechnete Gleichgewichts-Rotationskonstanten aufweisen und energetisch nahe beieinander liegen, wobei Isomer ***I*** etwas stabiler ist. Obwohl die Berücksichtigung der Schwingungsbeiträge stark darauf hindeutet, dass Isomer ***I*** die beobachtete Spezies ist, wurde die endgültige, unvoreingenommene Zuordnung mit Erfassung und Anpassung der Rotationsspektren der 9 monosubstituierten ^{13}C-Isotopologe und der (^{18}O)FA-Spezies erreicht. Die Rotationsdaten für mehrere Isotopologe ermöglichten auch die quantitative Strukturbestimmung (siehe Abschnitt S3).

Die ^{13}C-Isotopologe wurden in natürlicher Häufigkeit beobachtet. Das Menge der (^{18}O)FA-Spezies wurde durch Zugabe eines Tropfens $H_2{}^{18}O$ in die Trägergasleitung angehoben, da die Einbringung eine in-situ-Isotopenaustauschreaktion zur Bildung von (^{18}O)FA bewirkt.[21] Das Verhältnis von BZF-(^{18}O)FA und BZF-(^{16}O)FA im Überschallstrahl beträgt etwa 1:6, wie aus ihren Linienintensitäten ersichtlich ist. Im Fall der BZF-(^{18}O)FA-Spezies wurden die Rotationsübergänge beider Zustände, ʋ = 0 und ʋ = 1, angepasst, um präzise Rotationsparameter zu erhalten, während für die ^{13}C-Isotopologe die ausschließliche Beobachtung der stärksten Tunnelkomponente (*ʋ* = 1-Zustand) es lediglich ermöglichte, die spektroskopischen Parameter nur für diesen Zustand zu bestimmen. Bei der Anpassung wurden D_{JK}, D_K, d_1 und d_2 auf die entsprechenden Werte der Hauptspezies für den Zustand *ʋ* = 1 festgelegt. Wie bei der Hauptspezies wurden die Spektren aller Isotopologe mit Hilfe des SPFIT-Programms von Pickett[22] unter Verwendung des *S-reduzierten* halbstarren Hamiltonians von Watson in III^l-Darstellung angepasst. [23]

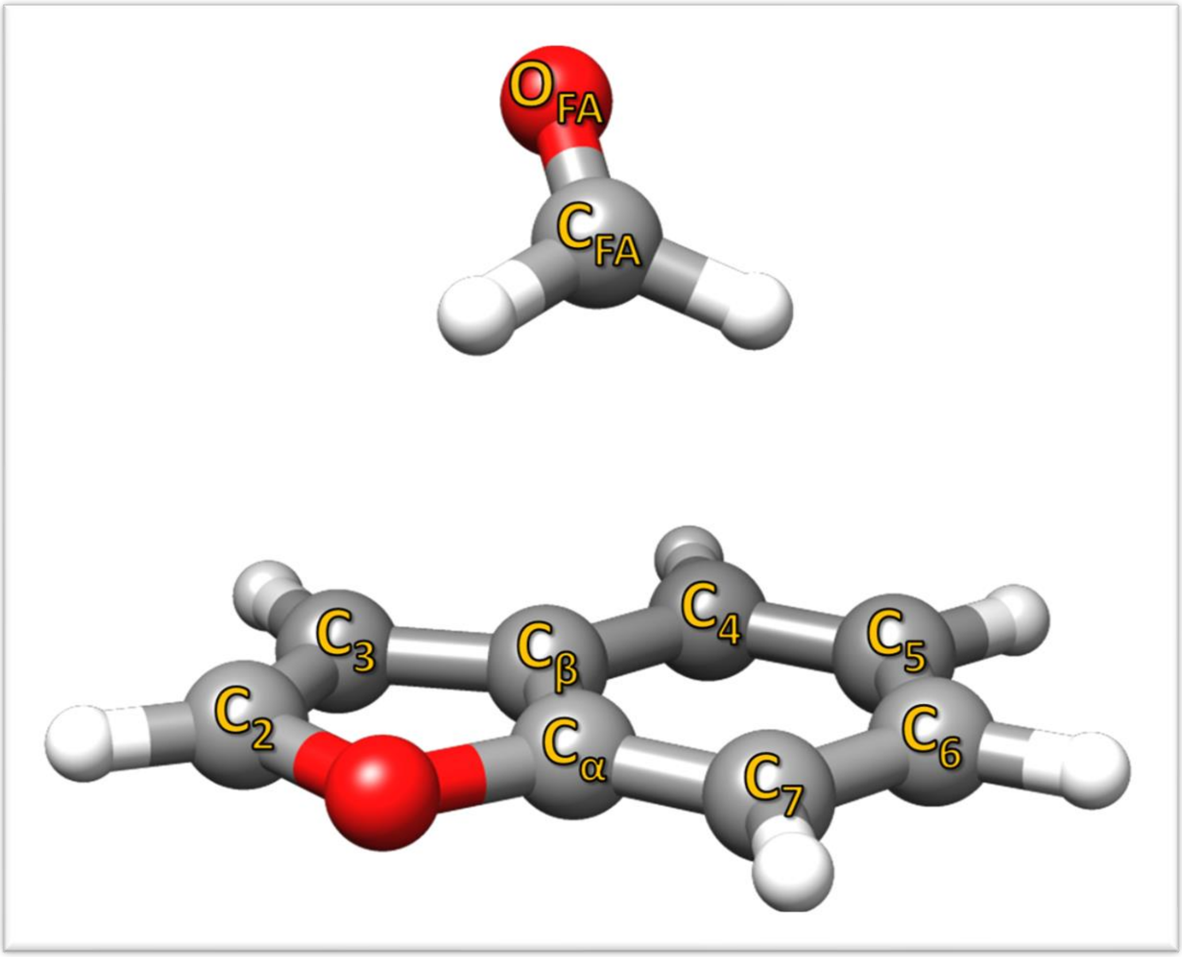

Abbildung SF2.1.1. Atombenennungen für das Isomer ***I*** des BZ-FA-Addukts

Die sorfältige Suche nach anderen Isomeren (***II*** bis ***XIV***) deckt den Frequenzbereich von 8,1-11,25 GHz ab (Integration von 384 Zyklen im Bereich von 8,1-9,8 GHz und Integration von 512 Zyklen im Bereich von 9,0-11,25 GHz). Es konnten jedoch keine Rotationsübergänge, die zu diesen Isomeren gehören, zugeordnet werden. Dies

stimmt mit den rechnerisch vorhergesagten sehr niedrigen Energiebarrieren überein, die zur Relaxation in die stabilste (und einzig beobachteten) Struktur (Isomer ***I***) führen. Für das wasserstoffgebundene Isomer ***VII*** wurde zusätzliche im Bereich von 14,9-16,5 MHz mit 0,4 MHz-Schritten gesucht, wobei jeweils bis zu 1024 Zyklen gemittelt wurde, so dass die Nichtentdeckung dieses Isomers auf die Konformationsrelaxation oder sehr schwache Signale zurückzuführen ist. [25]
Alle Anpassungsergebnisse sind in den nachstehenden Tabellen aufgeführt, gefolgt von den gemessenen Übergängen. Die Atombenennung des Isomers ***I*** des BZF-FA-Komplexes (zur Kennzeichnung der Isotopologe) ist in Abbildung SF2.1.1 dargestellt.

S2.2. Experimentelle Rotationsübergänge und Anpassungs-Ergebnisse von Isomer *I* des Benzofuran-Formaldehyd-Komplexes

S2.2.1. Die Hauptspezies.

Tabelle S2.2.1a. Experimentell bestimmte spektroskopische Parameter (Watson's *S-Reduktion*, III^l-Darstellung) für die Hauptspezies des Isomers ***I*** von BZF-FA.

Parameter	Experiment	
	$\upsilon = 0$	$\upsilon = 1$
A_0 [MHz]	1180.9038(2)[a]	1180.9045(2)
B_0 [MHz]	1096.1994(2)	1096.2033(2)
C_0 [MHz]	788.2780(1)	788.2808(1)
D_J [kHz]	3.902(2)	3.903(2)
D_{JK} [kHz]	-4.826(6)	-4.822(6)
D_K [kHz]	1.829(4)	1.828(4)
d_1 [kHz]	-0.448(2)	-0.448(2)
d_2 [kHz]	0.9641(8)	0.9639(8)
N[b]	204	
σ[c] [kHz]	2.7	

[a] Standardfehler in Einheiten der letzten Ziffer. [b] Anzahl der angepassten Linien; [c] RMS Fehler der Anpassung.

Tabelle S2.2.1b. Nummerierte (*N*) experimentelle Rotationsübergänge für die Hauptspezies des Isomers ***I*** von BZF-FA. Die Tabelle enthält die experimentellen Frequenzen (ν_{EXP}) und die Residuen (Δv: beobachtet - berechnet).

N	J	K_a	K_c	v	J'	K_a'	K_c'	v'	ν_{EXP} [MHz]	Δv [MHz]
1	3	1	3	0	2	1	2	0	5071.0350	-0.0021
2	3	1	3	1	2	1	2	1	5071.0534	-0.0001

3	3	0	3	0	2	0	2	0	5084.4732	-0.0020
4	3	0	3	1	2	0	2	1	5084.4951	0.0044
5	3	2	2	0	2	2	1	0	5653.0496	-0.0047
6	3	2	2	1	2	2	1	1	5653.0763	0.0022
7	3	1	2	0	2	1	1	0	5834.3696	-0.0023
8	3	1	2	1	2	1	1	1	5834.3896	0.0023
9	3	2	1	0	2	2	0	0	6221.7983	-0.0031
10	3	2	1	1	2	2	0	1	6221.8232	-0.0023
11	4	1	4	0	3	1	3	0	6653.0625	-0.0044
12	4	1	4	1	3	1	3	1	6653.0888	0.0010
13	4	0	4	0	3	0	3	0	6654.6198	-0.0058
14	4	0	4	1	3	0	3	1	6654.6463	0.0000
15	3	3	1	0	2	2	1	0	6966.8154	0.0006
16	3	3	1	1	2	2	1	1	6966.8221	-0.0010
17	3	3	0	0	2	2	1	0	7127.1212	0.0003
18	3	3	0	1	2	2	1	1	7127.1384	0.0045
19	4	2	3	0	3	2	2	0	7321.9193	-0.0046
20	4	2	3	1	3	2	2	1	7321.9461	-0.0002
21	4	1	3	0	3	1	2	0	7382.9407	-0.0019
22	4	1	3	1	3	1	2	1	7382.9613	0.0001
23	4	3	2	0	3	3	1	0	7805.5017	-0.0026
24	4	3	2	1	3	3	1	1	7805.5327	-0.0004
25	4	2	2	0	3	2	1	0	8110.3147	-0.0019
26	4	2	2	1	3	2	1	1	8110.3420	0.0023
27	5	0	5	0	4	1	4	0	8229.9541	0.0069
28	5	1	5	0	4	1	4	0	8229.9541	-0.0063
29	5	0	5	1	4	1	4	1	8229.9695	-0.0031
30	5	1	5	1	4	1	4	1	8229.9879	0.0021
31	5	0	5	0	4	0	4	0	8230.1076	0.0008
32	5	1	5	0	4	0	4	0	8230.1192	-0.0009
33	5	0	5	1	4	0	4	1	8230.1361	0.0039
34	5	1	5	1	4	0	4	1	8230.1361	-0.0093
35	4	3	1	0	3	3	0	0	8307.0390	-0.0007
36	4	3	1	1	3	3	0	1	8307.0746	-0.0013
37	5	1	4	0	4	2	3	0	8920.4381	-0.0019
38	5	1	4	1	4	2	3	1	8920.4645	-0.0014
39	5	2	4	0	4	2	3	0	8921.8628	0.0020
40	5	2	4	1	4	2	3	1	8921.8868	0.0003
41	5	1	4	0	4	1	3	0	8932.2507	0.0017
42	5	1	4	1	4	1	3	1	8932.2726	-0.0011
43	5	2	4	0	4	1	3	0	8933.6677	-0.0021
44	5	2	4	1	4	1	3	1	8933.6934	-0.0008
45	4	3	1	0	3	2	1	0	8973.9764	-0.0063
46	4	3	1	1	3	2	1	1	8974.0042	-0.0003
47	4	2	2	0	3	1	2	0	8990.2679	0.0000
48	4	2	2	1	3	1	2	1	8990.2931	-0.0004
49	4	1	3	0	3	0	3	0	9071.7132	0.0058
50	4	1	3	1	3	0	3	1	9071.7278	-0.0005
51	4	2	3	0	3	1	3	0	9081.7904	-0.0077
52	4	2	3	1	3	1	3	1	9081.8184	0.0005
53	4	3	2	0	3	2	2	0	9119.2561	-0.0087
54	4	3	2	1	3	2	2	1	9119.2832	0.0011
55	4	4	1	0	3	3	0	0	9142.6364	0.0036
56	4	4	1	1	3	3	0	1	9142.6364	-0.0018
57	4	4	0	0	3	3	0	0	9237.0325	-0.0006
58	4	4	0	1	3	3	0	1	9237.0428	-0.0001
59	4	4	1	0	3	3	1	0	9302.9372	-0.0018
60	4	4	1	1	3	3	1	1	9302.9485	-0.0004

61	4	4	0	0	3	3	1	0	9397.3367	-0.0025
62	4	4	0	1	3	3	1	1	9397.3518	-0.0019
63	5	3	3	0	4	3	2	0	9549.7913	0.0051
64	5	3	3	1	4	3	2	1	9549.8150	-0.0010
65	5	2	3	0	4	2	2	0	9706.1295	-0.0082
66	5	2	3	1	4	2	2	1	9706.1607	0.0019
67	4	3	1	0	3	2	2	0	9781.1076	0.0013
68	4	3	1	1	3	2	2	1	9781.1340	-0.0017
69	6	1	6	0	5	1	5	0	9806.1654	-0.0003
70	6	0	6	0	5	1	5	0	9806.1654	0.0008
71	6	0	6	0	5	0	5	0	9806.1786	0.0007
72	6	1	6	0	5	0	5	0	9806.1786	-0.0003
73	6	1	6	1	5	1	5	1	9806.1949	-0.0001
74	6	0	6	1	5	1	5	1	9806.1949	0.0009
75	6	1	6	1	5	0	5	1	9806.2090	0.0007
76	6	0	6	1	5	0	5	1	9806.2090	0.0018
77	5	4	2	0	4	4	1	0	9919.6061	0.0006
78	5	4	2	1	4	4	1	1	9919.6449	0.0005
79	5	4	1	0	4	4	0	0	10326.6519	-0.0034
80	5	4	1	1	4	4	0	1	10326.7054	-0.0001
81	5	3	2	0	4	3	1	0	10336.5866	0.0011
82	5	3	2	1	4	3	1	1	10336.6186	-0.0007
83	6	1	5	0	5	2	4	0	10501.3308	0.0043
84	6	1	5	1	5	2	4	1	10501.3559	-0.0002
85	6	2	5	0	5	2	4	0	10501.4746	0.0036
86	6	2	5	1	5	2	4	1	10501.4962	-0.0044
87	6	1	5	0	5	1	4	0	10502.7488	0.0015
88	6	1	5	1	5	1	4	1	10502.7764	-0.0003
89	5	4	2	0	4	3	1	0	10755.1983	-0.0004
90	5	4	2	1	4	3	1	1	10755.2044	-0.0022
91	6	2	4	0	5	3	3	0	11177.8370	0.0012
92	6	2	4	1	5	3	3	1	11177.8673	-0.0005
93	6	3	4	0	5	3	3	0	11184.7950	0.0011
94	6	3	4	1	5	3	3	1	11184.8246	-0.0003
95	5	3	2	0	4	2	2	0	11200.2538	0.0022
96	5	3	2	1	4	2	2	1	11200.2843	0.0002
97	6	2	4	0	5	2	3	0	11223.3117	0.0028
98	6	2	4	1	5	2	3	1	11223.3359	-0.0002
99	6	3	4	0	5	2	3	0	11230.2689	0.0018
100	6	3	4	1	5	2	3	1	11230.2945	0.0013
101	5	4	1	0	4	3	1	0	11256.6479	-0.0007
102	5	4	1	1	4	3	1	1	11256.6719	-0.0006
103	5	2	3	0	4	1	3	0	11313.4676	0.0046
104	5	2	3	1	4	1	3	1	11313.4910	-0.0002
105	5	3	3	0	4	2	3	0	11347.1330	0.0059
106	5	3	3	1	4	2	3	1	11347.1538	0.0021
107	5	1	4	0	4	0	4	0	11349.3360	0.0051
108	5	1	4	1	4	0	4	1	11349.3561	0.0005
109	5	2	4	0	4	1	4	0	11350.5909	-0.0011
110	5	2	4	1	4	1	4	1	11350.6161	-0.0005
111	7	0	7	0	6	0	6	0	11382.1938	-0.0008
112	7	1	7	0	6	1	6	0	11382.1938	0.0002
113	7	0	7	1	6	0	6	1	11382.2270	-0.0003
114	7	1	7	1	6	1	6	1	11382.2270	0.0006
115	5	4	2	0	4	3	2	0	11417.0375	-0.0027
116	5	4	2	1	4	3	2	1	11417.0595	-0.0008
117	5	5	1	0	4	4	0	0	11552.5365	-0.0045
118	5	5	1	1	4	4	0	1	11552.5365	-0.0110

119	5	5	0	0	4	4	0	0	11602.5426	0.0026
120	5	5	0	1	4	4	0	1	11602.5495	-0.0005
121	5	5	1	0	4	4	1	0	11646.9417	0.0005
122	5	5	1	1	4	4	1	1	11646.9509	-0.0014
123	5	5	0	0	4	4	1	0	11696.9377	-0.0025
124	5	5	0	1	4	4	1	1	11696.9531	-0.0017
125	6	4	3	0	5	4	2	0	11747.6048	0.0022
126	6	4	3	1	5	4	2	1	11747.6420	0.0008
127	6	3	3	0	5	3	2	0	12038.8989	-0.0013
128	6	3	3	1	5	3	2	1	12038.9264	0.0004
129	7	2	6	0	6	2	5	0	12077.5658	0.0078
130	7	2	6	1	6	2	5	1	12077.5865	-0.0046
131	7	1	6	0	6	1	5	0	12077.6961	0.0068
132	7	1	6	1	6	1	5	1	12077.7274	0.0049
133	6	4	3	0	5	3	2	0	12166.2162	0.0004
134	6	4	3	1	5	3	2	1	12166.2324	0.0039
135	6	4	2	0	5	4	1	0	12500.2449	-0.0020
136	6	4	2	1	5	4	1	1	12500.2958	0.0016
137	7	3	5	0	6	3	4	0	12772.7848	0.0004
138	7	3	5	1	6	3	4	1	12772.8173	-0.0007
139	7	2	5	0	6	2	4	0	12778.8901	0.0028
140	7	2	5	1	6	2	4	1	12778.9215	0.0014
141	8	0	8	0	7	0	7	0	12958.0730	0.0068
142	8	1	8	0	7	1	7	0	12958.0730	0.0069
143	8	0	8	1	7	0	7	1	12958.1016	0.0000
144	8	1	8	1	7	1	7	1	12958.1016	0.0001
145	6	5	2	0	5	4	1	0	13224.3273	-0.0011
146	6	5	2	1	5	4	1	1	13224.3344	-0.0001
147	6	4	2	0	5	3	2	0	13420.3106	0.0006
148	6	4	2	1	5	3	2	1	13420.3477	0.0003
149	7	4	4	0	6	4	3	0	13435.9927	0.0042
150	7	4	4	1	6	4	3	1	13436.0255	-0.0003
151	6	3	3	0	5	2	3	0	13533.0144	0.0002
152	6	3	3	1	5	2	3	1	13533.0522	0.0009
153	7	3	4	0	6	3	3	0	13538.6641	0.0003
154	7	3	4	1	6	3	3	1	13538.6916	0.0000
155	6	5	1	0	5	4	1	0	13570.2909	0.0066
156	6	5	1	1	5	4	1	1	13570.3087	0.0010
157	6	2	4	0	5	1	4	0	13604.5259	0.0030
158	6	2	4	1	5	1	4	1	13604.5554	0.0018
159	6	4	3	0	5	3	3	0	13614.8616	0.0050
160	6	4	3	1	5	3	3	1	13614.8857	0.0002
161	8	2	7	0	7	2	6	0	13653.1597	-0.0032
162	8	1	7	0	7	1	6	0	13653.1796	0.0047
163	8	2	7	1	7	2	6	1	13653.1967	-0.0023
164	8	1	7	1	7	1	6	1	13653.2126	0.0016
165	6	5	2	0	5	4	2	0	13725.7767	-0.0016
166	6	5	2	1	5	4	2	1	13725.7988	-0.0015
167	6	6	1	0	5	5	0	0	13947.3200	0.0078
168	6	6	1	1	5	5	0	1	13947.3200	-0.0005
169	6	6	0	0	5	5	0	0	13971.8722	-0.0019
170	6	6	0	1	5	5	0	1	13971.8855	0.0008
171	6	6	1	0	5	5	1	0	13997.3120	0.0008
172	6	6	1	1	5	5	1	1	13997.3246	0.0016
173	6	6	0	0	5	5	1	0	14021.8742	0.0011
174	6	6	0	1	5	5	1	1	14021.8879	0.0007
175	6	5	1	0	5	4	2	0	14071.7328	-0.0015
176	6	5	1	1	5	4	2	1	14071.7724	-0.0012

177	8	2	5	0	7	2	4	0	14349.3641	-0.0028
178	8	2	5	1	7	2	4	1	14349.4037	0.0001
179	7	4	3	0	6	4	2	0	14349.6260	-0.0018
180	7	4	3	1	6	4	2	1	14349.6608	-0.0019
181	8	2	6	0	7	2	5	0	14350.1289	-0.0023
182	8	2	6	1	7	2	5	1	14350.1682	0.0005
183	9	0	9	0	8	0	8	0	14533.7584	-0.0032
184	9	1	9	0	8	1	8	0	14533.7584	-0.0032
185	9	0	9	1	8	0	8	1	14533.7991	0.0003
186	9	1	9	1	8	1	8	1	14533.7991	0.0003
187	8	3	4	0	7	3	3	0	15042.6470	-0.0010
188	8	3	4	1	7	3	3	1	15042.6859	0.0000
189	8	3	5	0	7	3	4	0	15063.6630	0.0014
190	8	3	5	1	7	3	4	1	15063.6970	0.0004
191	9	1	8	0	8	1	7	0	15228.5970	-0.0070
192	9	2	8	0	8	2	7	0	15228.5970	-0.0060
193	9	1	8	1	8	1	7	1	15228.6416	-0.0006
194	9	2	8	1	8	2	7	1	15228.6416	0.0004
195	8	4	3	0	7	4	2	0	15667.9232	-0.0020
196	8	4	3	1	7	4	2	1	15667.9718	0.0016
197	8	4	4	0	7	4	3	0	15882.9506	-0.0008
198	8	4	4	1	7	4	3	1	15882.9776	-0.0016
199	9	3	7	0	8	3	6	0	15924.2669	-0.0029
200	9	3	7	1	8	3	6	1	15924.3092	0.0002
201	9	2	7	0	8	2	6	0	15924.3544	0.0023
202	9	2	7	1	8	2	6	1	15924.3935	0.0023
203	9	3	6	0	8	3	5	0	16625.1407	-0.0050
204	9	3	6	1	8	3	5	1	16625.1820	-0.0030

S2.2.2. Das BZF-(^{18}O)FA-Isotopologe.

Tabelle S2.2.2a. Experimentell ermittelte spektroskopische Parameter (Watson's *S-Reduktion*, in III^{l}-Darstellung) für das (^{18}O)FA-Isotopologe des Isomers ***I*** von BZF-FA.

Parameter	Experiment	
	$\upsilon = 0$	$\upsilon = 1$
A_0 [MHz]	1179.608(2)[a]	1179.6120(7)
B_0 [MHz]	1047.2712(6)	1047.2738(4)
C_0 [MHz]	762.0013(3)	762.0043(2)
D_J [kHz]	3.95(4)	3.865(9)
D_{JK} [kHz]	-5.11(8)	-4.84(2)
D_K [kHz]	2.09(4)	1.90(1)
d_1 [kHz]	-0.55(3)	-0.571(7)
d_2 [kHz]	1.072(7)	1.067(3)
N[b]	77	
σ[c] [kHz]	3.1	

[a] Standardfehler in Einheiten der letzten Ziffer. [b] Anzahl der angepassten Linien; [c] RMS Fehler der Anpassung.

Tabelle S2.2.2b. Nummerierte (*N*) experimentelle Rotationsübergänge für das (^{18}O)FA-Isotopologe des Isomers ***I*** von BZF-FA. Die Tabelle enthält die experimentellen Frequenzen (ν_{EXP}) und die Residuen ($\Delta\nu$: beobachtet - berechnet).

N	J	K_a	K_c	v	J'	K_a'	K_c'	v'	ν_{EXP} [MHz]	$\Delta\nu$ [MHz]
1	3	2	2	0	2	2	1	0	5427.4200	-0.0062
2	3	2	2	1	2	2	1	1	5427.4411	-0.0019
3	3	1	2	0	2	1	1	0	5657.6112	-0.0035
4	3	1	2	1	2	1	1	1	5657.6356	0.0043
5	3	2	1	0	2	2	0	0	5922.1979	0.0012
6	3	2	1	1	2	2	0	1	5922.2150	0.0000
7	4	1	4	0	3	1	3	0	6438.6171	-0.0061
8	4	1	4	1	3	1	3	1	6438.6433	-0.0001
9	4	0	4	0	3	0	3	0	6444.1917	0.0004
10	4	0	4	1	3	0	3	1	6444.2134	0.0016
11	4	2	3	0	3	2	2	0	7072.1501	0.0056
12	4	2	3	1	3	2	2	1	7072.1635	-0.0004
13	4	1	3	0	3	1	2	0	7195.4277	-0.0025
14	4	1	3	1	3	1	2	1	7195.4528	0.0014
15	4	3	2	0	3	3	1	0	7463.5674	0.0026
16	4	3	2	1	3	3	1	1	7463.5895	-0.0014
17	4	2	2	0	3	2	1	0	7819.2579	0.0039
18	4	2	2	1	3	2	1	1	7819.2797	0.0015
19	4	3	1	0	3	3	0	0	7847.5513	-0.0076
20	4	3	1	1	3	3	0	1	7847.5850	-0.0021
21	5	1	5	0	4	1	4	0	7964.4546	-0.0029
22	5	1	5	1	4	1	4	1	7964.4814	0.0000
23	5	0	5	0	4	0	4	0	7965.2904	-0.0014

24	5	0	5	1	4	0	4	1	7965.3154	-0.0004
25	5	2	4	0	4	2	3	0	8641.9112	0.0035
26	5	2	4	1	4	2	3	1	8641.9317	0.0035
27	5	1	4	0	4	1	3	0	8677.0918	0.0011
28	5	1	4	1	4	1	3	1	8677.1107	-0.0022
29	4	4	0	1	3	3	0	1	9173.1371	0.0004
30	5	3	3	0	4	3	2	0	9197.0212	0.0067
31	5	3	3	1	4	3	2	1	9197.0424	0.0010
32	4	4	1	1	3	3	1	1	9228.8906	0.0009
33	5	4	2	0	4	4	1	0	9447.4012	0.0049
34	5	4	2	1	4	4	1	1	9447.4399	0.0028
35	5	2	3	0	4	2	2	0	9470.9599	0.0022
36	5	2	3	1	4	2	2	1	9470.9869	-0.0006
37	6	1	6	0	5	1	5	0	9488.3340	0.0028
38	6	1	6	1	5	1	5	1	9488.3570	-0.0012
39	6	0	6	0	5	0	5	0	9488.4483	0.0057
40	6	0	6	1	5	0	5	1	9488.4681	-0.0016
41	5	4	1	0	4	4	0	0	9698.4451	-0.0031
42	5	4	1	1	4	4	0	1	9698.4903	-0.0007
43	5	3	2	0	4	3	1	0	9892.1052	0.0035
44	5	3	2	1	4	3	1	1	9892.1389	0.0025
45	6	2	5	0	5	2	4	0	10177.1587	-0.0056
46	6	2	5	1	5	2	4	1	10177.1830	-0.0010
47	6	1	5	0	5	1	4	0	10184.1937	0.0014
48	6	1	5	1	5	1	4	1	10184.2109	-0.0019
49	6	3	4	0	5	3	3	0	10825.7797	-0.0027
50	6	3	4	1	5	3	3	1	10825.8077	-0.0001
51	6	2	4	0	5	2	3	0	10944.1038	-0.0038
52	6	2	4	1	5	2	3	1	10944.1389	0.0006
53	7	1	7	0	6	1	6	0	11011.8106	0.0024
54	7	1	7	1	6	1	6	1	11011.8319	-0.0059
55	7	0	7	0	6	0	6	0	11011.8319	0.0099
56	7	0	7	1	6	0	6	1	11011.8546	0.0030
57	5	1	4	1	4	0	4	1	11045.2766	-0.0020
58	5	5	0	1	4	4	0	1	11544.5408	0.0026
59	5	5	1	1	4	4	1	1	11571.7225	-0.0028
60	7	2	6	0	6	2	5	0	11702.4026	-0.0002
61	7	2	6	1	6	2	5	1	11702.4142	-0.0053
62	7	1	6	0	6	1	5	0	11703.5633	-0.0059
63	6	3	3	0	5	3	2	0	11703.5836	-0.0048
64	7	1	6	1	6	1	5	1	11703.5836	-0.0026
65	6	3	3	1	5	3	2	1	11703.6283	-0.0020
66	7	2	5	1	6	2	4	1	12417.5720	-0.0014
67	8	0	8	0	7	0	7	0	12535.1020	-0.0029
68	8	1	8	0	7	1	7	0	12535.1020	-0.0013
69	8	0	8	1	7	0	7	1	12535.1343	-0.0023
70	8	1	8	1	7	1	7	1	12535.1343	-0.0007
71	8	2	7	1	7	2	6	1	13225.5915	0.0041
72	8	1	7	0	7	1	6	0	13225.7536	0.0052
73	8	1	7	1	7	1	6	1	13225.7641	0.0040
74	9	0	9	0	8	0	8	0	14058.2180	-0.0017
75	9	1	9	0	8	1	8	0	14058.2180	-0.0015
76	9	0	9	1	8	0	8	1	14058.2517	-0.0014
77	9	1	9	1	8	1	8	1	14058.2517	-0.0012

S2.2.3. Das ($^{13}C_2$)BZF-FA-Isotopologe.

Tabelle S2.2.3a. Experimentell ermittelte spektroskopische Parameter (Watson's *S-Reduktion*, III^{l}-Darstellung) für den υ = 1 Zustand des ($^{13}C_2$)BZF-Isotopologen des Isomers ***I*** von BZF-FA.

Parameter	**Experiment**
	υ = 1
A_0 [MHz]	1165.146(6)[a]
B_0 [MHz]	1095.96(1)
C_0 [MHz]	781.1750(8)
D_J [kHz]	3.939(8)
D_{JK} [kHz]	[-4.822(6)][b]
D_K [kHz]	[1.828(4)]
d_1 [kHz]	[-0.448(2)]
d_2 [kHz]	[0.9639(8)]
N[c]	10
σ[d] [kHz]	4.0

[a] Standardfehler in Einheiten der letzten Ziffer. [b] Die Werte in Klammern sind auf die entsprechenden Werte des υ = 1 Zustand der Hauptspezies fixiert. [c] Anzahl der angepassten Linien; d] RMS Fehler der Anpassug.

Tabelle S2.2.3b. Nummerierte (*N*) experimentelle Rotationsübergänge für den υ = 1 Zustand des ($^{13}C_2$)BZF-Isotopologen des Isomers ***I*** von BZF-FA. Die Tabelle enthält die experimentellen Frequenzen (ν_{EXP}) und die Residuen (Δv: beobachtet - berechnet).

N	J	K_a	K_c	J'	K_a'	K_c'	ν_{EXP} [MHz]	Δv [MHz]
1	4	1	4	3	1	3	6596.4662	0.0042
2	4	0	4	3	0	3	6597.3255	-0.0025
3	4	1	3	3	1	2	7316.2028	0.0004
4	5	0	5	4	0	4	8158.9421	0.0048
5	5	1	5	4	1	4	8158.8726	0.0011
6	5	1	4	4	1	3	8858.1212	-0.0007
7	6	1	6	5	1	5	9720.8273	-0.0077
8	6	0	6	5	0	5	9720.8358	-0.0036
9	7	1	7	6	1	6	11282.6457	0.0050
10	7	0	7	6	0	6	11282.6457	0.0047

S2.2.4. Das ($^{13}C_3$)BZF-FA-Isotopologe.

Tabelle S2.2.4a. Experimentell ermittelte spektroskopische Parameter (Watson's *S-Reduktion*, III^l-Darstellung) für den υ = 1-Zustand des ($^{13}C_3$)BZF-Isotopologen des Isomers ***I*** von BZF-FA.

Parameter	**Experiment**
	υ = 1
A_0 [MHz]	1169.799(4)
B_0 [MHz]	1092.838(6)
C_0 [MHz]	785.1122(5)
D_J [kHz]	3.893(5)
D_{JK} [kHz]	[-4.822(6)][b]
D_K [kHz]	[1.828(4)]
d_1 [kHz]	[-0.448(2)]
d_2 [kHz]	[0.9639(8)]
N[c]	10
σ[d] [kHz]	2.3

[a] Standardfehler in Einheiten der letzten Ziffer. [b] Die Werte in Klammern sind auf die entsprechenden Werte des υ = 1 Zustand der Hauptspezies festgelegt. [c] Anzahl der angepassten Linien; d] RMS Fehler der Anpassung.

Tabelle S2.2.4b. Nummerierte (*N*) experimentelle Rotationsübergänge für den υ = 1 Zustand des ($^{13}C_3$)BZF-Isotopologen des Isomers ***I*** von BZF-FA. Die Tabelle enthält die experimentellen Frequenzen (v_{EXP}) und die Residuen (Δv: beobachtet - berechnet).

N	J	K_a	K_c	J'	K_a'	K_c'	v_{EXP} [MHz]	Δv [MHz]
1	4	1	4	3	1	3	6624.2394	-0.0002
2	4	0	4	3	0	3	6625.4425	-0.0019
3	4	1	3	3	1	2	7341.9319	0.0000
4	5	1	5	4	1	4	8194.6659	-0.0003
5	5	0	5	4	0	4	8194.7706	0.0009
6	5	1	4	4	1	3	8888.1746	0.0002
7	6	1	6	5	1	5	9764.5268	-0.0032
8	6	0	6	5	0	5	9764.5433	0.0054
9	7	1	7	6	1	6	11334.2299	-0.0010
10	7	0	7	6	0	6	11334.2299	-0.0016

S2.2.5. Das ($^{13}C_{\alpha}$)BZF-FA-Isotopologe.

Tabelle S2.2.5a. Experimentell ermittelte spektroskopische Parameter (Watson's *S-Reduktion*, III^l-Darstellung) für den ʋ = 1 Zustand des ($^{13}C_{\alpha}$)BZF-Isotopologen des Isomers ***I*** von BZF-FA.

Parameter	**Experiment**
	$\upsilon = 1$
A_0 [MHz]	1179.780(5)
B_0 [MHz]	1093.874(8)
C_0 [MHz]	787.1308(6)
D_J [kHz]	3.890(4)
D_{JK} [kHz]	[-4.822(6)][b]
D_K [kHz]	[1.828(4)]
d_1 [kHz]	[-0.448(2)]
d_2 [kHz]	[0.9639(8)]
N[c]	10
σ[d] [kHz]	2.9

[a] Standardfehler in Einheiten der letzten Ziffer. [b] Die Werte in Klammern sind auf die entsprechenden Werte des ʋ = 1 Zustand der Hauptspezies festgelegt. [c] Anzahl der angepassten Linien; d] RMS Fehler der Anpassung.

Tabelle S2.2.5b. Nummerierte (*N*) experimentelle Rotationsübergänge für den Zustand ʋ = 1 des ($^{13}C_{\alpha}$)BZF-Isotopologen des Isomers ***I*** von BZF-FA. Die Tabelle zeigt die experimentellen Frequenzen (ν_{EXP}) und die Residuen (Δv: beobachtet - berechnet).

N	J	K_a	K_c	J'	K_a'	K_c'	ν_{EXP} [MHz]	Δv [MHz]
1	4	1	4	3	1	3	6643.2054	-0.0019
2	4	0	4	3	0	3	6644.8304	-0.0060
3	4	1	3	3	1	2	7372.6209	0.0001
4	5	1	5	4	1	4	8217.8400	0.0044
5	5	0	5	4	0	4	8217.9938	0.0026
6	5	1	4	4	1	3	8919.1053	0.0005
7	7	1	7	6	1	6	11365.4910	0.0011
8	7	0	7	6	0	6	11365.4910	0.0001
9	8	1	8	7	1	7	12939.0723	-0.0012
10	8	0	8	7	0	7	12939.0723	-0.0013

S2.2.6. Das ($^{13}C_β$)BZF-FA-Isotopologe.

Tabelle S2.2.6a. Experimentell ermittelte spektroskopische Parameter (Watson's *S-Reduktion*, III^l-Darstellung) für den υ = 1 Zustand des ($^{13}C_β$)BZF-Isotopologen des Isomers ***I*** von BZF-FA.

Parameter	**Experiment**
	$\upsilon = 1$
A_0 [MHz]	1178.786(7)
B_0 [MHz]	1094.24(1)
C_0 [MHz]	788.0239(9)
D_J [kHz]	3.915(9)
D_{JK} [kHz]	[-4.822(6)][b]
D_K [kHz]	[1.828(4)]
d_1 [kHz]	[-0.448(2)]
d_2 [kHz]	[0.9639(8)]
N[c]	10
σ[d] [kHz]	4.1

[a] Standardfehler in Einheiten der letzten Ziffer. [b] Die Werte in Klammern sind auf die entsprechenden Werte des υ = 1 Zustand der Hauptspezies fixiert. [c] Anzahl der angepassten Linien; d] RMS Fehler der Anpassung.

Tabelle S2.2.6b. Nummerierte (*N*) experimentelle Rotationsübergänge für den υ = 1 Zustand des ($^{13}C_β$)BZF-Isotopologen des Isomers ***I*** von BZF-FA. Die Tabelle enthält die experimentellen Frequenzen (v_{EXP}) und die Residuen (Δv: beobachtet - berechnet).

N	J	K_a	K_c	J'	K_a'	K_c'	v_{EXP} [MHz]	Δv [MHz]
1	4	1	4	3	1	3	6649.2459	0.0044
2	4	0	4	3	0	3	6650.8083	0.0017
3	4	1	3	3	1	2	7375.5507	0.0005
4	5	1	5	4	1	4	8225.6308	0.0055
5	5	0	5	4	0	4	8225.7658	-0.0070
6	5	1	4	4	1	3	8924.3651	-0.0011
7	6	1	6	5	1	5	9801.3129	-0.0039
8	6	0	6	5	0	5	9801.3258	-0.0034
9	7	1	7	6	1	6	11376.8330	0.0048
10	7	0	7	6	0	6	11376.8330	0.0038

S2.2.7. Das ($^{13}C_4$)BZF-FA-Isotopologe.

Tabelle S2.2.7a. Experimentell ermittelte spektroskopische Parameter (Watson's *S-Reduktion*, III^l-Darstellung) für den υ = 1 Zustand des ($^{13}C_4$)BZF-Isotopologen des Isomers ***I*** von BZF-FA.

Parameter	**Experiment**
	υ = 1
A_0 [MHz]	1172.73(1)
B_0 [MHz]	1090.81(1)
C_0 [MHz]	786.9611(4)
D_J [kHz]	3.890 (3)
D_{JK} [kHz]	[-4.822(6)][b]
D_K [kHz]	[1.828(4)]
d_1 [kHz]	[-0.448(2)]
d_2 [kHz]	[0.9639(8)]
N[c]	12
σ[d] [kHz]	2.7

[a] Standardfehler in Einheiten der letzten Ziffer. [b] Die Werte in Klammern sind auf die entsprechenden Werte des υ = 1 Zustand der Hauptspezies fixiert. [c] Anzahl der angepassten Linien; d] RMS Fehler der Anpassung.

Tabelle S2.2.7b. Nummerierte (*N*) experimentelle Rotationsübergänge für den υ = 1 Zustand des ($^{13}C_4$)BZF-Isotopologen des Isomers ***I*** von BZF-FA. Die Tabelle enthält die experimentellen Frequenzen (v_{EXP}) und die Residuen (Δv: beobachtet - berechnet).

N	J	K_a	K_c	J'	K_a'	K_c'	v_{EXP} [MHz]	Δv [MHz]
1	4	1	4	3	1	3	6637.2371	0.0024
2	4	0	4	3	0	3	6638.6916	-0.0002
3	5	1	5	4	1	4	8211.4568	-0.0005
4	5	0	5	4	0	4	8211.5868	-0.0049
5	5	1	4	4	1	3	8902.6359	0.0005
6	6	1	6	5	1	5	9785.0257	-0.0029
7	6	0	6	5	0	5	9785.0452	0.0056
8	6	1	5	5	1	4	10470.8335	-0.0005
9	7	1	7	6	1	6	11358.4296	0.0020
10	7	0	7	6	0	6	11358.4296	0.0012
11	8	1	8	7	1	7	12931.6715	-0.0011
12	8	0	8	7	0	7	12931.6715	-0.0011

S2.2.8. Das ($^{13}C_5$)BZF-FA-Isotopologe.

Tabelle S2.2.8a. Experimentell ermittelte spektroskopische Parameter (Watson's *S-Reduktion*, III^l-Darstellung) für den υ = 1-Zustand des ($^{13}C_5$)BZF-Isotopologen des Isomers ***I*** von BZF-FA.

Parameter	**Experiment**
	υ = 1
A_0 [MHz]	1168.897(3)
B_0 [MHz]	1092.213(5)
C_0 [MHz]	782.1475(4)
D_J [kHz]	3.909(4)
D_{JK} [kHz]	[-4.822(6)][b]
D_K [kHz]	[1.828(4)]
d_1 [kHz]	[-0.448(2)]
d_2 [kHz]	[0.9639(8)]
N[c]	10
σ[d] [kHz]	1.9

[a] Standardfehler in Einheiten der letzten Ziffer. [b] Die Werte in Klammern sind auf die entsprechenden Werte des υ = 1 Zustand der Hauptspezies fixiert. [c] Anzahl der angepassten Linien; d] RMS Fehler der Anpassung.

Tabelle S2.2.8b. Nummerierte (*N*) experimentelle Rotationsübergänge für den υ = 1 Zustand des ($^{13}C_5$)BZF-Isotopologen des Isomers ***I*** von BZF-FA. Die Tabelle enthält die experimentellen Frequenzen ($ν_{EXP}$) und die Residuen (Δv: beobachtet - berechnet).

N	J	K_a	K_c	J'	K_a'	K_c'	$ν_{EXP}$ [MHz]	Δv [MHz]
1	4	1	4	3	1	3	6602.7633	0.0036
2	4	0	4	3	0	3	6603.9389	0.0015
3	4	1	3	3	1	2	7324.6011	0.0002
4	5	1	5	4	1	4	8167.2402	-0.0025
5	5	0	5	4	0	4	8167.3408	-0.0020
6	5	1	4	4	1	3	8865.0997	-0.0006
7	6	1	6	5	1	5	9731.1732	0.0026
8	6	0	6	5	0	5	9731.1732	-0.0050
9	7	1	7	6	1	6	11294.9365	0.0019
10	7	0	7	6	0	6	11294.9365	0.0013

S2.2.9. Das ($^{13}C_6$)BZF-FA-Isotopologe.

Tabelle S2.2.9a. Experimentell ermittelte spektroskopische Parameter (Watson's *S-Reduktion*, III^{l}-Darstellung) für den υ = 1-Zustand des ($^{13}C_6$)BZF-Isotopologen des Isomers ***I*** von BZF-FA.

Parameter	**Experiment**
	υ = 1
A_0 [MHz]	1171.869(8)
B_0 [MHz]	1089.31(1)
C_0 [MHz]	781.581(1)
D_J [kHz]	3.900(1)
D_{JK} [kHz]	[-4.822(6)][b]
D_K [kHz]	[1.828(4)]
d_1 [kHz]	[-0.448(2)]
d_2 [kHz]	[0.9639(8)]
N[c]	10
σ[d] [kHz]	5.0

[a] Standardfehler in Einheiten der letzten Ziffer. [b] Die Werte in Klammern sind auf die entsprechenden Werte des υ = 1-Zustand der Hauptspezies fixiert. [c] Anzahl der angepassten Linien; d] RMS Fehler der Anpassung.

Tabelle S2.2.9b. Nummerierte (*N*) experimentelle Rotationsübergänge für den υ = 1 Zustand des ($^{13}C_6$)BZF-Isotopologen des Isomers ***I*** von BZF-FA. Die Tabelle enthält die experimentellen Frequenzen (v_{EXP}) und die Residuen (Δv: beobachtet - berechnet).

N	J	K_a	K_c	J'	K_a'	K_c'	v_{EXP} [MHz]	Δv [MHz]
1	4	1	4	3	1	3	6598.3775	-0.0089
2	4	0	4	3	0	3	6599.8381	-0.0048
3	4	1	3	3	1	2	7324.6091	-0.0006
4	5	1	5	4	1	4	8161.8552	0.0085
5	5	0	5	4	0	4	8161.9857	0.0053
6	5	1	4	4	1	3	8861.4152	0.0014
7	6	1	6	5	1	5	9724.6530	-0.0014
8	6	0	6	5	0	5	9724.6675	0.0022
9	7	1	7	6	1	6	11287.2858	-0.0030
10	7	0	7	6	0	6	11287.2858	-0.0038

S2.2.10. Das ($^{13}C_7$)BZF-FA-Isotopologe.

Tabelle S2.2.10a. Experimentell ermittelte spektroskopische Parameter (Watson's *S-Reduktion*, III^l-Darstellung) für den ʋ = 1 Zustand des ($^{13}C_7$)BZF-Isotopologen des Isomers ***I*** von BZF-FA.

Parameter	**Experiment**
	$\upsilon = 1$
A_0 [MHz]	1175.922(4)
B_0 [MHz]	1088.025(7)
C_0 [MHz]	785.4454 (5)
D_J [kHz]	3.884(5)
D_{JK} [kHz]	[-4.822(6)][b]
D_K [kHz]	[1.828(4)]
d_1 [kHz]	[-0.448(2)]
d_2 [kHz]	[0.9639(8)]
N[c]	10
σ[d] [kHz]	2.4

[a] Standardfehler in Einheiten der letzten Ziffer. [b] Die Werte in Klammern sind auf die entsprechenden Werte des ʋ = 1 Zustand der Hauptspezies festgelegt. [c] Anzahl der angepassten Linien; d] RMS Fehler der Anpassung.

Tabelle S2.2.10b. Nummerierte (*N*) experimentelle Rotationsübergänge für den Zustand ʋ = 1 des ($^{13}C_7$)BZF-Isotopologen des Isomers ***I*** von BZF-FA. Die Tabelle zeigt die experimentellen Frequenzen (ν_{EXP}) und die Residuen (Δν: beobachtet - berechnet).

N	J	K_a	K_c	J'	K_a'	K_c'	ν_{EXP} [MHz]	Δν [MHz]
1	4	1	4	3	1	3	6626.3452	-0.0041
2	4	0	4	3	0	3	6628.1198	-0.0011
3	4	1	3	3	1	2	7350.6855	-0.0004
4	5	1	5	4	1	4	8197.6637	0.0007
5	5	0	5	4	0	4	8197.8435	0.0055
6	5	1	4	4	1	3	8892.8782	0.0007
7	6	1	6	5	1	5	9768.2152	-0.0015
8	6	0	6	5	0	5	9768.2325	0.0004
9	7	1	7	6	1	6	11338.5857	-0.0002
10	7	0	7	6	0	6	11338.5857	-0.0014

S2.2.11. Das BZF-(^{13}C)FA-Isotopologe.

Tabelle S2.2.11a. Experimentell ermittelte spektroskopische Parameter (Watson's *S-Reduktion*, III^{l}-Darstellung) für den υ = 1 Zustand des (^{13}C)FA-Isotopologs des Isomers ***I*** von BZF-FA.

Parameter	**Experiment**
	υ = 1
A_0 [MHz]	1178.526(4)
B_0 [MHz]	1082.367(8)
C_0 [MHz]	781.4010(6)
D_J [kHz]	3.886(6)
D_{JK} [kHz]	[-4.822(6)][b]
D_K [kHz]	[1.828(4)]
d_1 [kHz]	[-0.448(2)]
d_2 [kHz]	[0.9639(8)]
N[c]	10
σ[d] [kHz]	2.6

[a] Standardfehler in Einheiten der letzten Ziffer. [b] Die Werte in Klammern sind auf die entsprechenden Werte des υ = 1 Zustand der Hauptspezies fixiert. [c] Anzahl der angepassten Linien; d] RMS Fehler der Anpassung.

Tabelle S2.2.11b. Nummerierte (*N*) experimentelle Rotationsübergänge für den Zustand υ = 1 des (^{13}C)FA-Isotopologen des Isomers ***I*** von BZF-FA. Die Tabelle enthält die experimentellen Frequenzen ($ν_{EXP}$) und die Residuen (Δν: beobachtet - berechnet).

N	J	K_a	K_c	J'	K_a'	K_c'	$ν_{EXP}$ [MHz]	Δν [MHz]
1	4	1	4	3	1	3	6595.7776	-0.0023
2	4	0	4	3	0	3	6598.0496	0.0029
3	4	1	3	3	1	2	7329.5968	-0.0004
4	5	1	5	4	1	4	8159.1956	0.0020
5	5	0	5	4	0	4	8159.4402	0.0023
6	5	1	4	4	1	3	8860.4043	0.0002
7	6	1	6	5	1	5	9721.6752	-0.0035
8	6	0	6	5	0	5	9721.6983	-0.0039
9	7	1	7	6	1	6	11283.9607	0.0040
10	7	0	7	6	0	6	11283.9607	0.0019

S3. Semi-experimentelle Gleichgewichtsstrukturen

S3.1. Theoretische Grundlagen.

Die Suche nach genauen Strukturen ist von grundlegender Bedeutung bei nicht-kovalenten Wechselwirkungen, die in der Natur eine große Rolle spielen. Der semi-experimentelle Ansatz der Gleichgewichtsstruktur r_e^{SE} bietet eine Möglichkeit, die Parameter mit guter Genauigkeit zu bestimmen.[26,27] Diese Methode erfordert die experimentellen Rotationskonstanten der Schwingungsgrundzustände $B^i_{\mathrm{exp},0}$ für einen (möglicherweise) großen Satz von Isotopologen, die mit quantenchemisch berechneten Schwingungsbeiträgen ΔB^i_{vib} kombiniert werden.
Zur Bewertung aller oder ausgewählter geometrischer Parameter wird eine Fehlerquadratminimierung semi-experimenteller Gleichgewichtsrotationskonstanten $B^i_{\mathrm{SE},e}$ durchgeführt, wozu Grundzustandsrotationskonstanten $B^i_{\mathrm{exp},0}$ um berechnete Schwingungsbeiträge $\Delta B^i_{\mathrm{calc},vib}$ korrigiert werden. Die Auswertung der Beiträge nach Gleichung (3) im Rahmen der Schwingungsstörungstheorie zweiter Ordnung (VPT2)[28] erfordert die Berechnung der Schwingungs-Rotations-Wechselwirkungskonstanten α^i_r und damit anharmonische Kraftfeldberechnungen (auf B3-Niveau für isoliertes BZF und CP-B3 für das Isomer ***I*** von BZF-FA):

$$B^i_{\mathrm{SE},e} = B^i_{\mathrm{exp},0} - \Delta B^i_{\mathrm{calc,vib}} = B^i_{\mathrm{exp},0} + \frac{1}{2}\sum_r \alpha^i_r \qquad (3)$$

In der obigen Gleichung läuft die Summe über die *r* Schwingungsnormalmoden und *i* bezieht sich auf die Trägheitsachsen (*a*, *b*, *c*).
In der vorliegenden Untersuchung wurden zwei semi-experimentelle Strukturen bestimmt: die von Benzofuran (BZF) und die des Isomers ***I*** des BZF-FA-Addukts. Diese werden in den Abschnitten S3.2 bzw. S3.3 ausführlich beschrieben.
Trotz der großen Anzahl von Isotopensubstitutionen kann für das BZF-FA-Addukt keine vollständige r_e^{SE}-Struktur ermittelt werden. Mit dem "Template-Modell"-Ansatz (TM)[27] können jedoch die intramolekularen Parameter der beiden Molekülfragmente (BZF und FA) genau bestimmt und in der Fehlerquadratminimierung für das molekulare Addukt fixiert werden. Nach dem TM-Ansatz werden die intramolekularen Parameter von BZF und FA innerhalb des Komplexes wie folgt bestimmt:

$$r_e^{\mathrm{SE}}(\mathrm{intramolecular, adduct}) = r_e^{QM}(\mathrm{adduct}) + \Delta TM \qquad (4)$$

wobei

$$\Delta\mathrm{TM} = r_e^{\mathrm{SE}}(\mathrm{monomer}) - r_e^{\mathrm{QM}}(\mathrm{monomer}) \qquad (5)$$

Hier ist $r_e^{\mathrm{QM}}(\mathrm{adduct})$ ein generischer intramolekularer Molekülkomplexesparameter, der auf einem bestimmten Theorieniveau optimiert wurde (im vorliegenden Fall jB2), und ΔTM die Differenz zwischen dem semi-experimentellen Parameter für das isolierte Monomer und seinem quantenchemischen Wert ist (auf demselben Niveau

wie das für Addukt, hier jB2). Die Anwendung des TM-Modells wie oben beschrieben setzt voraus, dass die r_e^{SE}-Strukturen beider Fragmente bekannt sind. Während die r_e^{SE} von FA in der Literatur verfügbar ist (aus der SMART-Lab-Datenbank [27]), wurde die r_e^{SE} von BZF für diese Arbeit abgeschätzt (siehe Abschnitt S3.2).
Für einen gegebenen Parameter, dessen $r_e^{SE}(\mathrm{monomer})$ nicht verfügbar ist, wird der r_e^{QM} Wert (in dieser Studie auf jB2 Niveau) unkorrigiert verwendet.

S3.2. Benzofuran.

Eine genaue r_e^{SE}-Struktur des isolierten Benzofurans wurde auf der Grundlage der in der Literatur verfügbaren Rotationskonstanten von 10 Isotopologen (Hauptspezies, alle mono-substituierten ^{13}C- und ^{18}O-Spezies) bestimmt. [29]

Tabelle S3.2.1. Die r_e^{SE}-Struktur von Benzofuran.

C						
C	1	R1				
C	2	R2	1	A1		
C	1	R3	2	A2	3	D0
H	1	R4	2	A3	3	D180
H	2	R5	1	A4	5	D0
H	3	R6	2	A5	6	D0
H	4	R7	1	A6	5	D0
C	3	R8	2	A7	1	D0
C	4	R9	1	A8	2	D0
C	9	R10	3	A9	2	D180
O	10	R11	4	A10	1	D180
H	11	R12	9	A11	3	D0
C	11	R13	9	A12	3	D180
H	14	R14	12	A13	10	D180
**angepasste PARAMETER**				**auf B2-Niveau festgelegte PARAMETER**		
R1		1.4036(4)		R4		1.08023
R2		1.3861(3)		R5		1.08036
A1		121.29(1)		R6		1.08057
R3		1.3875(3)		R7		1.07940
A2		121.41(1)		R12		1.07528
R8		1.3995(6)		R14		1.07428
A7		118.29(2)		A3		119.35051
R9		1.3848(6)		A4		119.09253
A8		116.25(2)		A5		120.74438
R10		1.4399(5)		A6		122.14555
A9		135.61(2)		A11		127.88372
R11		1.3651(4)		A13		115.20154
A10		125.72(3)		D0		0.0
R13		1.3515(4)		D180		180.0
A12		105.77(2)				

Gemäß Gleichung (3) wurden die semi-experimentellen Gleichgewichts-Rotationskonstanten der einzelnen Isotopologe durch Korrektur der experimentellen Grundzustands-Rotationskonstanten um die B3-Schwingungsbeiträge gewonnen. In der Fehlerquadratminimierung wurden die Parameter, die die Wasserstoffatome betreffen, auf ihre jB2-Werte festgelegt. Benzofuran ist ein planares Molekül (der Trägheitsdefekt Δ_c sollte daher Null sein), wobei alle betrachteten Haupt- und isotopensubstituierten Spezies im Schwingungsgrundzustand ein $\Delta_c \approx$-0,06 uÅ^2 aufweisen. Bei einer Strukturbestimmung, die Planarität voraussetzt, sind nur zwei Rotationskonstanten jedes Isotopologen linear unabhängig und im Anpassungsverfahren verwendbar. Die ermittelte Struktur des isolierten Benzofurans ist in Tabelle S3.2.1 mit den 15 angepassten Parameter (σ^2=2·10^{-6}) aufgeführt. In Tabelle S3.2.2 sind die verwendeten experimentellen Rotationskonstanten, zusammen mit den entsprechenden Schwingungskorrekturen aufgeführt

Tabelle S3.2.2. Grundzustands-Rotationskonstanten[a] und entsprechende B3-Schwingungsbeiträge für die verschiedenen Isotopologe von Benzofuran. Werte in MHz. Die Nummerierung der Isotopologe folgt derjenigen in Tabelle S3.2.1.

Isotopologes	A_0	B_0	C_0	A_{vib}	B_{vib}	C_{vib}
PARENT	3916.565	1660.795	1166.418	-29.202	-10.321	-7.571
$^{13}C_1$	3900.582	1636.727	1153.101	-28.940	-10.159	-7.464
$^{13}C_2$	3903.641	1635.481	1152.750	-29.000	-10.139	-7.459
$^{13}C_3$	3855.715	1655.198	1158.221	-28.630	-10.238	-7.490
$^{13}C_4$	3857.094	1656.390	1158.930	-28.650	-10.241	-7.492
$^{13}C_9$	3898.733	1660.587	1164.730	-28.956	-10.257	-7.518
$^{13}C_{10}$	3904.842	1660.539	1165.251	-28.910	-10.263	-7.518
$^{13}C_{11}$	3875.783	1646.803	1155.897	-28.810	-10.186	-7.473
$^{18}O_{12}$	3848.624	1635.757	1148.039	-28.625	-10.081	-7.407
$^{13}C_{14}$	3916.629	1631.908	1152.101	-29.120	-10.129	-7.460

[a] Aus Ref. [29] auf die dritte Dezimalstelle gerundet.

S3.3. Das Isomer ***I*** des Benzofuran-Formaldehyd-Komplexes.

Die isolierten Benzofuran- und Formaldehydverbindungen wurden als Templatmoleküle verwendet. Ihre r_e^{SE}-Strukturen (BZF: Abschnitt S3.2; FA: CCse-Struktur[27], verfügbar unter smart.sns.it/molecules/) liefern somit die Korrekturen der intramolekularen geometrischen Parameter (ΔTM), die auf die entsprechenden CP-jB2-Berechnungswerte des Isomers ***I*** des BZF-FA-Addukts anzuwenden sind:

$$r_e^{SE}(\text{intramolecular}) = r_e^{CP-jB2} + \Delta\text{TM} \qquad (6)$$

wobei

$$\Delta\text{TM} = r_e^{SE}(\text{BZF/FA}) - r_e^{jB2}(\text{BZF/FA}) \qquad (7)$$

Auf der intramolekular korrigierten CP-jB2-Struktur wurden drei intermolekulare Parameter (der $C_{FA}\cdots C_{\beta}$ Abstand: 3,2257±0,0006 Å; der $C_{FA}\cdots C_{\beta}$-C_4 Winkel: 90,18±0,04°; der $C_{FA}\cdots C_{\beta}$-C_4-C_3 Winkel: -98,31±0,03°) mit Hilfe einer Fehlerquadratminimierung (σ^2 = 0,2) angepasst, um die elf Sätze von semi-experimentellen Gleichgewichtsrotationskonstanten zu reproduzieren.
Die angepassten Daten (Rotationskonstanten und Schwingungsbeiträge) sind in Tabelle S3.3.1 zusammengestellt. Abbildung SF3.3.1 zeigt eine grafische Darstellung der r_e^{SE} -Struktur des BZF-FZ-Komplexes (Isomer ***I***), ausgewählte intermolekulare Parameter finden sich in Tabelle S3.3., wobei die semi-experimentelle Struktur in kartesischen Koordinaten in Tabelle S3.3.3 angegeben ist.

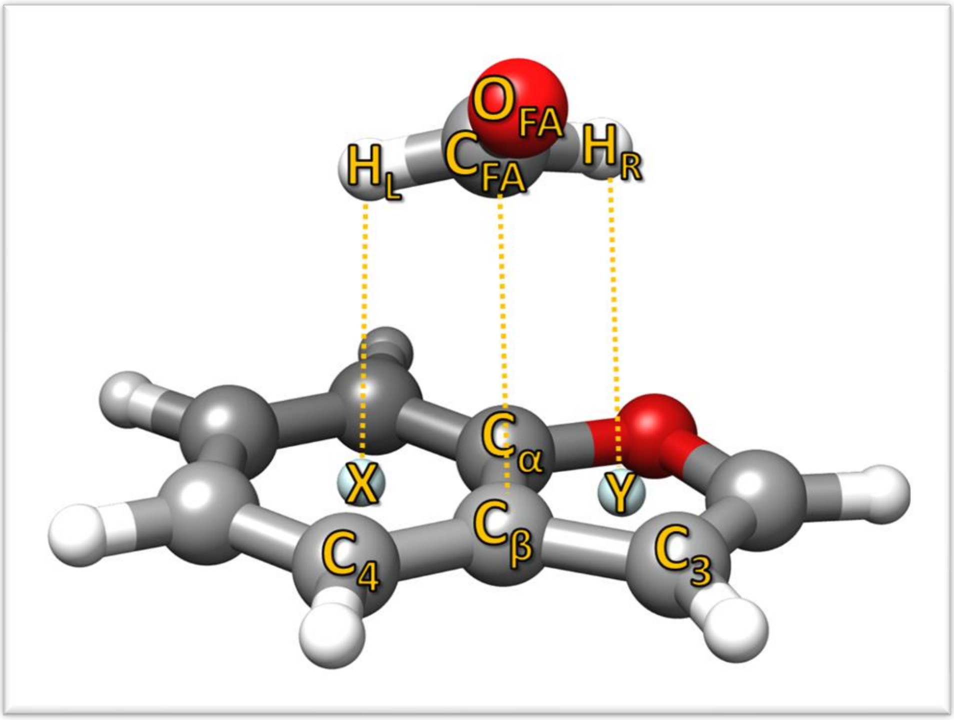

Abbildung SF3.3.1. Signifikante intermolekulare Kontakte im Isomer ***I*** des BZF-FA-Addukts. Die Dummy-Atome X und Y sind die Massenschwerpunkte der Benzol- bzw. Furanringe.

Tabelle S3.3.1. Grundzustands-Rotationskonstanten und die entsprechenden CP-B3-Schwingungsbeiträge. Werte in MHz.

Isotopologes	A_0	B_0	C_0	A_0-A_e	B_0-B_e	C_0-C_e
PARENT	1180.904	1096.203	788.281	-9.224	-20.572	-9.628
^{13}C(FA)	1178.526	1082.367	781.401	-8.736	-16.717	-8.269
^{18}O(FA)	1179.612	1047.274	762.004	-10.032	-20.691	-10.023
$^{13}C_2$	1165.146	1095.958	781.175	-10.074	-18.838	-8.669
$^{13}C_3$	1169.799	1092.838	785.112	-9.194	-21.380	-9.969
$^{13}C_4$	1172.729	1090.807	786.961	-9.292	-21.356	-10.185
$^{13}C_5$	1168.897	1092.213	782.147	-10.363	-21.303	-10.371
$^{13}C_6$	1171.869	1089.308	781.581	-10.697	-20.589	-10.086
$^{13}C_7$	1175.922	1088.025	785.445	-9.909	-21.479	-10.075
$^{13}C_\alpha$	1179.780	1093.874	787.131	-8.366	-18.725	-9.128
$^{13}C_\beta$	1178.786	1094.244	788.024	-7.914	-18.958	-9.166

Tabelle S3.3.2. Signifikante r_e^{SE} intermolekulare Parameter des Isomers ***I*** des BZF-FA-Addukts.

**Intermolekularer Parameter**	r_e^{SE}
$C_{FA}\cdots C_\beta$ [Å]	3.2257
$H_L\cdots X$ [Å]	2.7861
$H_R\cdots Y$ [Å]	2.9619
O_{FA}-$C_{FA}\cdots C_\beta$ [°]	114.99
$C_{FA}\cdots C_\beta$-C_α [°]	81.77
$C_{FA}\cdots C_\beta$-C_4 [°]	90.18
$C_{FA}\cdots C_\beta$-C_4-C_3 [°]	-98.31

Tabelle S3.3.3. Die $r_{\mathrm{e}}^{\mathrm{SE}}$-Struktur des Isomers ***I*** des BZF-FA-Addukts in kartesischen Koordinaten (Å).

C	0.00000000	0.00000000	0.00000000
C	0.00000000	0.00000000	1.40399000
C	1.18425116	0.00000000	2.12409082
C	1.18441514	-0.00091494	-0.72380817
H	-0.94156131	0.00010386	-0.52942830
H	-0.94388522	0.00343746	1.92925271
H	1.17460835	0.00935617	3.20424726
H	1.19787142	-0.00108682	-1.80318428
C	2.39134925	-0.00083046	1.41502558
C	2.35457410	-0.00250545	0.01727197
C	3.78809016	0.01723913	1.76387679
O	3.62074807	0.01354914	-0.49192283
H	4.22173796	0.03047788	2.74757254
C	4.45951980	0.02477999	0.59128354
H	5.50787255	0.03848583	0.35732041
C	2.13568032	3.18179590	0.95604476
O	2.30409688	3.85424805	1.94319161
H	2.96813270	2.93287765	0.28051602
H	1.14727709	2.78504551	0.68539483

S4. Referenzen

[1] P. Pracht, F. Bohle, S. Grimme, *Phys. Chem. Chem. Phys.* **2020**, *22*, 7169-7192.

[2] a) A. D. Becke, *J. Chem. Phys*. **1993**, *98*, 5648-5652; b) P.J. Stephens, F.J. Devlin, C.F. Chabalowski, M.J. Frisch, *J. Phys. Chem.* **1994**, *98*, 11623-11627; c) V. Barone, P. Cimino, E. Stendardo, *J. Chem. Theory Comput.* **2008**, *4*, 751-764; d) SNSD-Basissatz verfügbar unter: https://smart.sns.it/; e) S. Grimme, J. Antony, S. Ehrlich, H. Krieg *J. Chem. Phys*. **2010**, *132*, 154104; f) S. Grimme, S. Ehrlich, L. Goerigk, *J. Comp. Chem*. **2011**, *32*, 1456-1465;

[3] a) S. Grimme, *J. Chem. Phys*. **2006**, *124*, 034108; b) M. Biczysko, G. Scalmani, J. Bloino, V. Barone, *J. Chem. Theory Comput.* 2010, **6**, 2115-2125; c) E. Papajak, D. G. Truhlar, *J. Chem. Theory Comput.* **2011**, *7*, 10-18; d) E. Papajak, J. Zheng, X. Xu, H. R. Leverentz, D. G. Truhlar, J. *Chem. Theory Comput.* **2011**, 7, 3027–3034.

[4] S. F. Boys, F. Bernardi, *Mol. Phys*. **1970**, *19*, 553-566.

[5] S. Alessandrini, V. Barone, C. Puzzarini, *J. Chem. Theory Comput*. **2020**, *16*, 988−1006.

[6] K. Raghavachari, G. W. Trucks, J. A. Pople, M. Head-Gordon, *Chem. Phys. Lett.* **1989**, *157*, 479–483.

[7] C. Møller, M. S. Plesset, *Phys. Rev.* **1934**, *46*, 618-622.

[8] T. Helgaker, W. Klopper, H. Koch, J. Noga, *J. Chem. Phys.* **1997**, *106*, 9639-9646.

[9] K. A. Peterson, T. H. Dunning, *J. Chem. Phys.* **2002**, *117*, 10548-10560.

[10] Gaussian 16, Revision C.01, M. J. Frisch, G. W. Trucks, H. B. Schlegel, G. E. Scuseria, M. A. Robb, J. R. Cheeseman, G. Scalmani, V. Barone, G. A. Petersson, H. Nakatsuji, X. Li, M. Caricato, A. V. Marenich, J. Bloino, B. G. Janesko, R. Gomperts, B. Mennucci, H. P. Hratchian, J. V. Ortiz, A. F. Izmaylov, J. L. Sonnenberg, D. Williams-Young, F. Ding, F. Lipparini, F. Egidi, J. Goings, B. Peng, A. Petrone, T. Henderson, D. Ranasinghe, V. G. Zakrzewski, J. Gao, N. Rega, G. Zheng, W. Liang, M. Hada, M. Ehara, K. Toyota, R. Fukuda, J. Hasegawa, M. Ishida, T. Nakajima, Y. Honda, O. Kitao, H. Nakai, T. Vreven, K. Throssell, J. A. Montgomery, Jr, J. E. Peralta, F. Ogliaro, M. J. Bearpark, J. J. Heyd, E. N. Brothers, K. N. Kudin, V. N. Staroverov, T. A. Keith, R. Kobayashi, J. Normand, K. Raghavachari, A. P. Rendell, J. C. Burant, S. S. Iyengar, J. Tomasi, M. Cossi, J. M. Millam, M. Klene, C. Adamo, R. Cammi, J. W. Ochterski, R. L. Martin, K. Morokuma, O. Farkas, J. B. Foresman, and D. J. Fox, Gaussian, Inc, Wallingford CT, **2016**.

[11] E. D. Glendening, J, K. Badenhoop, A. E. Reed, J. E. Carpenter, J. A. Bohmann, C. M. Morales, P. Karafiloglou, C. R. Landis, F. Weinhold, Theoretical Chemistry Institute, University of Wisconsin, Madison, **2018**; b) E. D. Glendening, C. R. Landis, and F. Weinhold, *WIREs Comput. Mol. Sci*. **2012**, *2*, 1-42.

[12] a) A. D. Becke, *J. Chem. Phys*. **1993**, *98*, 5648-5652; b) P.J. Stephens, F.J. Devlin, C.F. Chabalowski, M.J. Frisch, *J. Phys. Chem.* **1994**, *98*, 11623-11627.

[13] a) T. Fornaro, M. Biczysko, J. Bloino, V. Barone, *Phys. Chem. Chem. Phys.* **2016**, *18*, 8479-8490; b) E. Papajak, H. R. Leverentz, J. Zheng, D. G. Truhlar, *J. Chem. Theory Comput.* **2009**, *5*, 1197–1202.

[14] a) B. Jeziorski, R. Moszynski, K. Szalewicz, *Chem. Rev*. **1994**, *94*, 1887 - 1930; b) T. M. Parker, L. A. Burns, R. M. Parrish, A. G. Ryno, C. D. Sherrill, *J. Chem. Phys*. **2014**, *140*, 094106.

[15] R. M. Parrish, L. A. Burns, D. G. A. Smith, A. C. Simmonett, A. E. DePrince III, E. G. Hohenstein, U. Bozkaya, A. Y. Sokolov, R. Di Remigio, R. M. Richard, J. F. Gonthier, A. M. James, H. R. McAlexander, A. Kumar, M. Saitow, X. Wang, B. P. Pritchard, P. Verma, H. F. Schaefer III, K. Patkowski, R. A. King, E. F. Valeev, F. A. Evangelista, J. M. Turney, T. Daniel Crawford, C. D. Sherril, *J. Chem. Theory Comput*. **2017**, *13*, 3185 – 3197.

[16] Y. Shi, J. Zhang, M. Shi, S. P. O'Connor, S. N. Bisaha, C. Li, D. Sitkoff, A. T. Pudzianowski, S. Chong, H. E. Klei, K. Kish, J. Yanchunas, E. C.-K. Liu, K. S. Hartl, S. M. Seiler, T. E. Steinbacher, W. A. Schumacher, K. S. Atwal, P. D. Stein, *Bioorganic Med. Chem. Lett.* **2009**, *19*, 4034-4041.

[17] H.M. Berman, J. Westbrook, Z. Feng, G. Gilliland, T.N. Bhat, H. Weissig, I.N. Shindyalov, P.E. Bourne, *Nucleic Acids Res*.**2000**, *28*,235-242.

[18] E.F. Pettersen, T.D. Goddard, C.C. Huang, G.S. Couch, D.M. Greenblatt, E.C. Meng, T.E. Ferrin, *J*. *Comput*. *Chem*. **2004**, 25,1605-1612.[19] J.-U. Grabow, W. Stahl, H. Dreizler, *Rev*. *Sci*. *Instrum*. **1996**, *67*, 4072–4084.
[20] J.-U. Grabow, Q. Gou, G. Feng, 72nd International Symposium on Molecular Spectroscopy, **2017**, TH03.
[21] FTMW++ program: J.-U. Grabow, Habilitationsschrift, Universität Hannover, Hannover, **2004**. verfügbar unter: https://seafile.projekt.uni-hannover.de/d/b05d0b38d02c4bc79969/
[22] H. M. Pickett, *J*. *Mol*. *Spectrosc*. **1991**, *148*, 371–377.
[23] J. K. G. Watson, in Vibrational Spectra and Structure, herausgegeben von J. R. Durig (Elsevier, New York, Amsterdam) **1977**, Bd. 6, S. 1-89.
[24] W. Li, A. Maris, C. Calabrese, I. Usabiaga, W. D. Geppert, L. Evangelisti, S. Melandri, *Phys*. *Chem*. *Chem*. *Phys*. **2019**, *21*, 23559-23566.
[25] R. S. Ruoff, T. D. Klots, T. Emilsson, H. S. Gutowsky, *J. Chem*. *Phys*. **1990**, *93*, 3142-3150.
[26] a) P. Pulay, W. Meyer, J. E. Boggs, *J. Chem. Phys.* **1978**, *68*, 5077-5085; b) F. Pawłowski, P. Jørgensen, J. Olsen, F. Hegelund, T. Helgaker, J. Gauss, K. L. Bak, J. F. Stanton, *J. Chem. Phys.* **2002**, *116*, 6482-6496.
[27] M. Piccardo, E. Penocchio, C. Puzzarini, M. Biczysko, V. Barone, *J. Phys. Chem. A* **2015**, *119*, 2058−2082.
[28] a) I. M. Mills, Vibration-Rotation Structure in Asymmetric- and Symmetric-Top Molecules. In *Molecular Spectroscopy: Modern Research*; Rao, K.N., Mathews, C.W., Eds.; Academic Press: New York, NY, USA, **1972**. b) V. Barone, *J. Chem*. *Phys*. **2005**, *122*, 014108.
[29] A. Maris, B. M.Giuliano, S. Melandri, P. Ottaviani, W. Caminati, L. B. Favero, B. Velino, *Phys. Chem. Chem. Phys.* **2005**,*7*, 3317-3322.
